# Unravelling the Role of Rumen Microbial Communities, Genes, and Activities on Milk Fatty Acid Profile Using a Combination of Omics Approaches

**DOI:** 10.3389/fmicb.2020.590441

**Published:** 2021-01-21

**Authors:** Sokratis Stergiadis, Irene Cabeza-Luna, Marina Mora-Ortiz, Robert D. Stewart, Richard J. Dewhurst, David J. Humphries, Mick Watson, Rainer Roehe, Marc D. Auffret

**Affiliations:** ^1^School of Agriculture, Policy and Development, Department of Animal Sciences, University of Reading, Animal, Dairy and Food Chain Sciences, Reading, United Kingdom; ^2^Beef and Sheep Research Centre, Scotland's Rural College (SRUC), Roslin Institute Building, Edinburgh, United Kingdom; ^3^The Roslin Institute, University of Edinburgh, Edinburgh, United Kingdom; ^4^Dairy Research and Innovation Centre, Scotland's Rural College (SRUC), Dumfries, United Kingdom; ^5^Centre for Dairy Research, University of Reading, Reading, United Kingdom

**Keywords:** rumen microbiome, microbial genes, cow, milk fatty acids, metagenomics, metabolomics, rumen stress

## Abstract

Milk products are an important component of human diets, with beneficial effects for human health, but also one of the major sources of nutritionally undesirable saturated fatty acids (SFA). Recent discoveries showing the importance of the rumen microbiome on dairy cattle health, metabolism and performance highlight that milk composition, and potentially milk SFA content, may also be associated with microorganisms, their genes and their activities. Understanding these mechanisms can be used for the development of cost-effective strategies for the production of milk with less SFA. This work aimed to compare the rumen microbiome between cows producing milk with contrasting FA profile and identify potentially responsible metabolic-related microbial mechanisms. Forty eight Holstein dairy cows were fed the same total mixed ration under the same housing conditions. Milk and rumen fluid samples were collected from all cows for the analysis of fatty acid profiles (by gas chromatography), the abundances of rumen microbiome communities and genes (by whole-genome-shotgun metagenomics), and rumen metabolome (using 500 MHz nuclear magnetic resonance). The following groups: (i) 24 High-SFA (66.9–74.4% total FA) vs. 24 Low-SFA (60.2–66.6%% total FA) cows, and (ii) 8 extreme High-SFA (69.9–74.4% total FA) vs. 8 extreme Low-SFA (60.2–64.0% total FA) were compared. Rumen of cows producing milk with more SFA were characterized by higher abundances of the lactic acid bacteria *Lactobacillus, Leuconostoc*, and *Weissella*, the acetogenic Proteobacteria *Acetobacter* and *Kozakia, Mycobacterium*, two fungi (*Cutaneotrichosporon* and *Cyphellophora*), and at a lesser extent *Methanobrevibacter* and the protist *Nannochloropsis*. Cows carrying genes correlated with milk FA also had higher concentrations of butyrate, propionate and tyrosine and lower concentrations of xanthine and hypoxanthine in the rumen. Abundances of rumen microbial genes were able to explain between 76 and 94% on the variation of the most abundant milk FA. Metagenomics and metabolomics analyses highlighted that cows producing milk with contrasting FA profile under the same diet, also differ in their rumen metabolic activities in relation to adaptation to reduced rumen pH, carbohydrate fermentation, and protein synthesis and metabolism.

## Introduction

In Western industrialized countries, human diets are generally characterized by saturated fatty acid (SFA) intakes which exceed nutritional recommendations, with milk and dairy products providing from 27 to 57% of all SFA intake in the UK (Givens, 2010). Excessive consumption of SFA has been associated with increased risk of cardiovascular diseases (CVD) in humans (Givens, 2010) whilst the source of fatty acids (FA) also has an effect on CVD risk [World Health Organization (WHO), 2003; Givens, 2010]. However, in addition to SFA, milk and dairy products provide a range of monounsatured FA (MUFA) and polyunsaturated FA (PUFA) that are beneficial for human health (Haug et al., 2007; Barcelo-Goblijn and Murphy, 2009; Swanson et al., 2012). Therefore, health organizations (FAO, 2008; EFSA Panel on Dietetic Products, 2010; Bates et al., 2014; SACN, 2018) and recent studies (Kliem and Shingfield, 2016; Nettleton et al., 2017) have urgently recommended the substitution of SFA in human diets, with *cis-*monounsaturated FA (*cis-*MUFA) and *cis-*polyunsaturated FA (*cis-*PUFA), in order to reduce the mortality from, and risk of, CVD (Givens, 2008). Given that milk fat is the largest single contributor of SFA in the UK (Bates et al., 2014), producing milk (a product widely consumed by all age groups) where part of its SFA has been replaced with *cis-*MUFA and/or *cis-*PUFA might be an effective means of contributing to the dietary targets, without requiring changes in consumer eating habits. Cow diets influence milk FA profile and provide opportunities to reduce milk SFA content, but proposed dietary interventions for the reduction of milk SFA content increase production costs [e.g., oil or oilseed supplementation (Stergiadis et al., 2014; Kliem and Shingfield, 2016)] or risk productivity of high-yielding cows [e.g., increased pasture intake (Chilliard et al., 2007; Ferris, 2007; Glasser et al., 2008; Stergiadis et al., 2012, 2015)]. As a result, these strategies are scarcely used in UK dairy systems and the % SFA in total FA in UK retail milk has changed little since 2006 [68.9% in 2016–2017 (Stergiadis et al., 2019); 70.7% in 2006–2007 (Butler et al., 2011)].

Milk fatty acid profiles generally present much higher concentrations of SFA and lower concentrations of PUFA than the FA profile in ruminant diets. This is partly due to the fat synthesis in the mammary gland, which produces a substantial amount of SFA with 4–16 atoms of carbon (Harfoot and Hazlewood, 1988). In addition, the presence of specific microbial species within the rumen microbiome perform lipolysis and subsequent biohydrogenation of the FA in the rumen (Lourenco et al., 2010); a process that result in C18:0 as the finite end product whilst producing a large amount of new unsaturated FA as intermediates (Jenkins et al., 2008; Ferlay et al., 2017). The rumen microbiome is a complex ecosystem, mainly composed of anaerobic bacteria and completed by archaea, ciliate protozoa and anaerobic fungi (Huws et al., 2013). These communities play different, but also interacting roles, which are essential for their maintenance and cooperative metabolic activities such as plant fiber degradation, carbohydrate fermentation, protein digestion, and synthesis of volatile fatty acids, amino acids, microbial protein and ammonia (Huws et al., 2013; Jami et al., 2014). The rumen microbiome is recognized for having an impact on animal performance traits such as health, methane emissions and feed efficiency (Russell, 1998; Jami et al., 2014; Auffret et al., 2017). In addition, there is large variation in phenotypic responses within ruminant breed and diet groups (Henderson et al., 2015; Roehe et al., 2016) but information explaining variation in milk FA profile, as well as the microbial communities and mechanisms behind such variation, are still, to our knowledge, limited. Buitenhuis et al. (2019) demonstrated the beneficial effect of adding rumen bacterial microbiome information to host genetic data for breeding purposes, and the impact on milk FA composition, but discussion on the potential mechanistic functions was not developed.

The overall aim of our work was to (i) compare the rumen microbiome between Holstein cows which, under the same diet, produce milk with contrasting FA profile, (ii) identify the rumen microbial taxa and genes impacting on individual FA and FA groups which are associated to human health, and (iii) identify metabolic-related microbial mechanisms controlling milk FA content. We hypothesized that differing microbial communities in the rumen of cows fed the same diet will influence the extent of rumen biohydrogenation and so potentially affect the milk FA profile. Given the overarching importance of diet in shaping rumen microbiome composition and its activity (Karisa et al., 2013; Petri et al., 2013; Rooke et al., 2014), we considered that combining metagenomics and metabolomics for studying the rumen fluid ecosystem provides an opportunity to identify microbial mechanisms associated with animals producing extreme contents of milk SFA (e.g., representing the lowest and the highest range). Both omic methodologies are considered complementary to better understand biological interaction networks and biological mechanisms associated with the variation in important traits in livestock production (Karisa et al., 2013; Pitta et al., 2016).

## Materials and Methods

### Ethics Approval

The animal experiment was conducted at the Centre for Dairy Research (CEDAR), University of Reading, UK; all experimental procedures used were licensed, regulated, and inspected by the UK Home Office under the Animals (Scientific Procedures) Act, 1996.

### Animals, Experimental Design and, Samples Collection

Four hundred and fifty two Holstein lactating cows were housed in a purpose built cubicle yard with sand as bedding, and water supplied throughout the day via self-filling drinking troughs. Cows were in mid-lactation between days 90 and 210 in milk. They received the same total mixed ratio (at forage:concentrate ratio of 63:37 on a dry matter basis), throughout the study. Forage was comprised of 71% maize silage and 29% grass silage (on a dry matter basis). Concentrate feed was made of commercial pellet (blend), sodagrain (sodium hydroxide-treated wheat), Megalac (rumen-protected fat supplement; Volac Wilmar Feed Ingredients, Hertfordshire, UK) and salt. Nutrient composition, digestibility, metabolisable energy (ME) content, volatile compounds and fatty acid profiles are presented in Supplementary Table 1. Dry matter intake (DMI) was back-calculated from ME requirements (Agricultural Food Research Council, 1993; Thomas, 2004) and ME content of the diet. Milk yield was automatically recorded in the milking parlor. Initially, the whole herd was screened for milk composition and FA profiles by Fourier Transform InfraRed spectroscopy (FTIR; MilkoScanTM 7RM; FOSS, Denmark), at the National Milk Laboratories (Wolverhampton, UK); using a composite morning and afternoon milk sample per cow. Based on the FTIR results, two groups of cows were created to represent the lowest range (low-SFA, *n* = 24 cows, 60.2–66.6% total FA) and highest range (high-SFA, *n* = 24 cows, 66.9–74.4% total FA) of milk SFA contents within this population. After 5 days, milk samples were collected from these cows (*n* = 48) and sent for immediate analysis of fat and protein content by FTIR, whilst an aliquot was stored in −20°C until FA analysis by gas chromatography (GC). Simultaneously, ~50 mL of rumen liquid were taken from the same 48 cows by inserting a stomach tube (16 × 2,700 mm Equivet Stomach Tube, Jørgen Kruuse A/S, Langeskov, Denmark) nasally and aspirating manually. This liquid was filtered through two layers of muslin and 5 mL strained rumen fluid still containing small particles were mixed with 10 ml phosphate buffered saline containing glycerol (30% v/v). These samples were stored at −80°C between collection and analysis. This technique is a practical means to sample a large number of cows for subsequent microbial community profiling, gene abundance analysis, and metabolite profiling; as well as previously presented sample preparation and storage techniques (Claus et al., 2008; Wallace et al., 2014; Firkins and Yu, 2015; Snelling et al., 2019). The technique has shown high microbiome similarity between rumen fluid samples collected using stomach tube and rumen digesta collected at the abattoir (Wallace et al., 2014; Snelling et al., 2019). Results from the FA analysis by GC were used to classify animals in two ways for subsequent data analysis (i) 24 cows with the lowest SFA concentrations vs. 24 cows with the highest SFA concentrations (Supplementary Table 2) and (ii) 8 cows with the extreme-low SFA concentrations (60.2–64.0% total FA) vs. 8 cows with the extreme-high SFA concentrations (69.9–74.4% total FA) (Supplementary Table 3).

### Milk Fatty Acid Profiles Analysis

Milk FA profiling was performed using GC coupled with flame ionization detection (Bruker 350 GC, Bruker, Germany) according to previously described methods of esterification and methylation (Viant, 2007), and techniques of peak identification and quantification (Chilliard et al., 2009; Kliem et al., 2013). A combined correction factor, to account for carbon deficiency in the response of flame ionization detector for FA methyl esters with 4–10 atoms of carbon was used (Ulberth et al., 1999). Milk FA were expressed as proportion of individual FA or FA group in total FA. In order to confirm that both experimental groups (24 vs. 24 and 8 vs. 8 separately) had significant differences for FA profile and potentially for other milk characteristics, one-way ANOVA with adjustment for multiple comparisons using the Bonferroni correction (SPSS Statistics 22, IBM, USA) applied.

### Genomic Analysis

DNA was extracted from the rumen fluid samples; all 48 samples were prepared for sequencing. Illumina TruSeq libraries were prepared from genomic DNA and sequenced on a NovaSeq 6000 instrument by Edinburgh Genomics (Edinburgh, UK). Paired-end reads (2 × 150 bp) were generated, resulting in between 10 and 24 GB per sample (between 33 and 80 million paired reads). A metadata file indicating the correspondence between animal and metagenomics IDs as well as grouping information based on milk SFA is summarized in Supplementary Table 4. Analysis of the functional content of the metagenomic data by comparing to the KEGG database (http://www.kegg.jp) followed the same procedure as previously described in Wallace et al. (2015) and Roehe et al. (2016). Statistical analysis of the metagenomics samples was based on the complete sample profiles, as expressed by the pattern of metagenomic reads classified within KEGG ortholog groups with >90% similarity and belonging to a single KEGG ortholog (KO) groups and the relative abundance (percentage) of individual KO group in each profile to normalize the data between animals. The alignment of the reads generated by whole metagenomic sequencing to the KEGG genes database resulted in identification of 4,660 microbial genes for each animal. Microbial genes with a relative abundance >0.001% (*n* = 1,630) were carried forward for downstream analysis.

For taxonomic annotation, we constructed a custom database using Kraken (v.0.10.5) (Wood and Salzberg, 2014). The database consisted of 7,318 complete bacterial genomes, 229 fungal genomes, 585 archaeal genomes and 75 protozoan genomes (all from RefSeq; 09/2018) augmented with 410 genomes from the Hungate collection and recently published 913 RUG genomes (Stewart et al., 2018). This database was used to assign reads to entries in the NCBI taxonomy database at the level of Kingdom, Phylum, Family, and Genus. Results with zero counts in 3 or more of the 48 animals were removed from the analysis to avoid statistical limitations due to interferences in the study of correlations. Following this step, 1,630 genes, 42 phyla, and 849 genera were selected for the statistical analysis.

Statistical analysis of the metagenomic data was based on the complete sample profiles as expressed by the pattern of metagenomic variables (taxonomic level or genes) and the relative abundance (percentage) of individual variable in each profile. Unless otherwise stated, all parameters used were the default.

Taxonomic and gene abundances were compared individually or within a functional group (e.g., all genes associated with low SFA) using a general linear model with adjustment for multiple comparisons using the Bonferroni correction (SPSS Statistics 22, IBM, USA).

In order to identify the rumen microbial species and genes on individual FA and FA groups which are associated to human health (and in particular SFA), we used partial least squares (PLS) analysis (Version 9.1 for Windows, SAS Institute Inc., Cary, NC, USA) to identify the most important genes associated with the milk SFA content. For the metagenomics analysis, the PLS analysis accounts for multiple testing and the correlation between taxonomic levels or microbial genes as variables. The model selections were based on the variable importance for projection (VIP) criterion (Wold, 1995), whereby variables with a VIP <0.8 contribute little to the prediction. Importance of a particular taxon or gene identified by PLS analysis within 8 extreme-low or 8 extreme-high animals to explain variation in a specific milk fatty acid was also determined by heatmaps. In order to identify the microbial origin of the genes that influence the concentrations of milk SFA, we used partial least squares (PLS) analysis (Version 9.1 for Windows, SAS Institute Inc., Cary, NC, USA) to identify microbial genera mostly correlated with each specific gene.

### Metabolomics Analysis of Rumen Fluids

A Bruker Avance HD 700 MHz (Bruker Biopsin, Rheinstetten, Germany) was used to acquire the NMR spectra from the ruminal biofluids (Saleem et al., 2013). The NMR was equipped with a TCI CryoProbes from the same manufacturer and was operating at 7,000 Hz. 1H NMR spectra was obtained using a standard 1D noesypr1d pulse sequences with a total acquisition time of 3.34 s with noesypr1d 90° pulse length of 7.7 μs (Meiboom and Gill, 1958; Aue et al., 1975). Water pre-saturation was applied for 3 s. The mixing time was set up at 50 or 100 ms. In total 64 scans per sample were accumulated into 64 K data points.

For the metabolomics analysis, all spectra were pre-processed in MestReNova version 11.0.2–18,153 (Mestrelab Research S.L., Spain) where it was phased and baseline corrected. The algorithm used for the baseline correction was Whittaker smoother; multipoint baseline correction was used if necessary (Dieterle et al., 2006). TSP (3-(trimethylsilyl)-propionic acid-d4; δ 0.00) was used for the calibration of all the samples. Residual water (δ 4.70–5.10) and noise regions (spectra before δ 0.5 and after δ 9.5) were removed in MestReNova before importing the data into MATLAB. Version R2015b of MATLAB (Mathworks, UK) equipped with Korrigan Toolbox version 0.1 (Korrigan Sciences Ltd., U.K.) was used to carry out the data analysis. Relationships between milk FA in the rumen and milk were evaluated from the loading plots of Principal Component Analysis (PCA) using Gen-Stat 16th edition (VSN International Ltd., UK). In order to correlate microbial genes with specific rumen metabolites and explain their metabolic mechanisms that have a positive effect on milk FA profile, the statistical approach started with a preliminary unsupervised Principal Component Analysis to evaluate the variability of the data. This was followed by a supervised pairwise Orthogonal Projection to Latent Structures Discriminant Analysis (O-PLS DA) (Cloarec et al., 2005; Bylesjö et al., 2006). This algorithm allowed the identification of specific modulations produced by metabolic changes associated with the relative abundance of specific genes previously identified as important to explain a trait. Models were evaluated for coefficient of determination (R^2^Y value) and goodness of prediction (Q^2^Y value, calculated using 7-fold cross-validation). The best models (R^2^ close to 1, Q^2^ positive and small overfit between R^2^ and Q^2^) were included in a second step of the pipeline where a selection of the samples with the lowest and highest concentration of the gene (*n* = 16) were used to identify metabolomics changes associated with the gene, used as predictor in each case. Metabolomic annotation was carried out using publicly available literature (Saleem et al., 2013), Chenomx NMR Suite 8.2 from Chenomx Inc (Edmonton, Canada) and online publicly available databases such as the Biological Magnetic Resonance data bank (BMRB, http://www.bmrb.wisc.edu) and the Bovine Metabolome Database (http://www.cowmetdb.ca/cgi-bin/browse.cgi).

## Results

### Variation in Animal Performance and Milk Fatty Acids

Dry matter intake was slightly higher (ANOVA, *P* = 0.042) for High-SFA cows than Low-SFA cows (Supplementary Figure 1A; 24 vs. 24 cows). This result was more pronounced with the 8 vs. 8 extreme cows (*P* = 0.013; results not shown).

**Figure 1:**
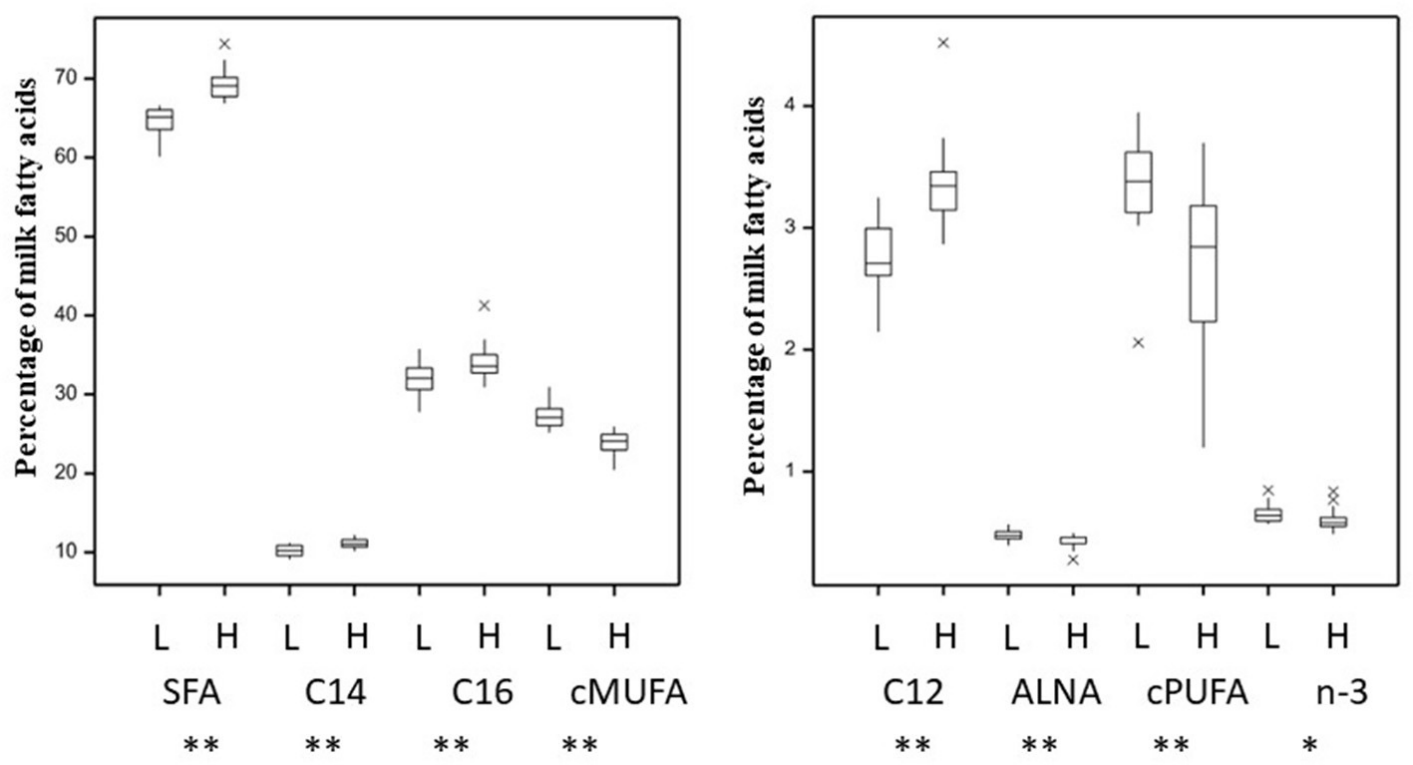
Variation in milk saturated (SFA, C12, C14, C16) and unsaturated (*cis-*MUFA, *cis-*PUFA, ALNA, and *n-*3) fatty acids (as a percentage of total fatty acids) for 48 animals grouped between Low-(L) and High-(H) milk SFA. *P < 0.05, **P < 0.01.

The total SFA content in milk, produced animals in the 24 v 24 dataset, was 66.9 ± 3.06% whilst *cis-*MUFA and *cis-*PUFA represented 25.5 ± 2.30% and 3.0 ± 0.64%, respectively (Supplementary Table 2). The total SFA content in milk, produced by the 16 animals in the 8 vs. 8 dataset, was 66.9 ± 4.92% whilst *cis-*MUFA and *cis-*PUFA represented 25.5 ± 3.64% and 2.8 ± 0.87%, respectively (Supplementary Table 3). The *cis n-*3, which were represented by 72% α-linoleic acid (ALNA, C18:3 c9, c12, c15) across the data, were 0.63 ± 0.08% of total FA for the 48 animals dataset and 0.62 ± 0.09% of total FA for the extremes 16 animals dataset (Supplementary Tables 2, 3, respectively). A principal component analysis to elucidate metabolic relationships between rumen and milk fatty acids (Supplementary Figure 2, Supplementary Tables 2, 5) showed in general a similar grouping of each fatty acid groups except for *cis-*MUFA. This difference was mainly explained by the first axis explaining >99% of variability. The most abundant individual SFA (C12:0, C14:0, and C16:0) were found in higher concentrations (*P* < 0.01) in milk from the High-SFA than from Low-SFA cows (Figure 1). Unsaturated fatty acids (UFA) were more abundant for Low-SFA than High-SFA cows (*P* < 0.01). ALNA and *n-*3 were both higher for Low-SFA milk whilst the relative difference in *n-*3 was smaller (*P* < 0.05). The transfer of linoleic acids (LA, C18:2 c9, c12), but not ALNA, from diet to milk was 1.19-fold higher in Low-SFA compared with High-SFA cows (Supplementary Figure 1B). This transfer difference was stronger in extreme animals with the transfer of dietary LA 1.49-fold higher in Low-SFA compared with High-SFA cows. There was no significant difference in parity or milk yield between the Low-SFA and High-SFA groups (Supplementary Figures 1A,C) or when comparing the extreme cows. Furthermore, there was no significant difference in stage of lactation between the Low-SFA (166 ± 37 days) and High-SFA (153 ± 34 days) groups.

### Variation in the Abundance of Rumen Microbial Communities in Cows With Contrasting Milk SFA Content

The rumen microbial communities were mainly composed of *Firmicutes, Bacteroidetes* and *Proteobacteria* corresponding on average to 61, 22, and 4%, respectively. *Euryarchaeota*, mostly composed of methanogens, represented about 3% of the total community. At the phylum level, no significant differences were observed between Low-SFA and High-SFA groups for the 48 animals (Supplementary Figure 3A) even when the comparison was performed in the extreme SFA groups (Supplementary Figure 3B). The Firmicutes-to-Bacteroidetes ratio was not correlated (*P* > 0.1) with milk FA content.

Comparison of microbial population relative abundances differed (ANOVA, *P* < 0.05) between SFA groups using the 48 (*n* = 37) and extreme animals with 37 and 18 genera, respectively, and showing a limited number of shared genera (*n* = 5) mostly within *Actinobacteria* (4/5), *Denitrobacterium* and the *Firmicutes Jeotgalibacillus* sp. (Table 1). Genera that were more abundant (*P* < 0.05) in High-SFA animals, within the 48 animals, included low abundant *Actinobacteria* (<0.01%; *n* = 12) and other bacteria and archaea. In addition, three methanogens (*Methanobrevibacter, Methanobacterium* and *Methanothermus* sp.), and two highly abundant genera (>0.01%), the protist *Nannochloropsis* and the *Proteobacteria Burkholderia* showed higher abundance (*P* < 0.05) in High-SFA animals (Table 1). Furthermore, 13 genera were more abundant (*P* < 0.05) in High-SFA animals, but only when the 16 extreme cows were compared, and were composed of 3 *Actinobacteria* including *Mycobacterium*, 3 lactic acid bacteria (LAB) within *Firmicutes* (*Lactobacillus, Leuconostoc* and *Weissella*), 3 *Proteobacteria* (*Kozakia, Hafnia* and *Komagataeibacter*) and fungi (5/13) such as *Cutaneotrichosporon* and *Cyphellophora* (Table 1). *Megasphaera* and *Selenomas* genera known to contain lactic acid-degrading species were not significantly different in any of the comparisons.

**Table 1:** ANOVA (Bonferroni corrected) results for genera significantly different between Low- compared to High-SFA groups (based on all 48 animals).

**Domain**	**Phylum**	**Genus**	**Mean LOW**	**Mean HIGH**	***P*-value**
**Comparison between Low- compared to High-SFA groups for the 48 animals**					
Bacteria	*Actinobacteria*	*Actinoplanes*	0.0046	0.0060	0.0497
Bacteria	*Actinobacteria*	*Aeromicrobium*	0.0013	0.0017	0.0380
Archaea	*Stramenopiles*	*Aureococcus*	0.0034	0.0045	0.0399
Bacteria	*Proteobacteria*	*Burkholderia*	0.0317	0.0415	0.0383
Bacteria	*Chloroflexi*	*Caldilinea*	0.0014	0.0018	0.0349
Bacteria	*Actinobacteria*	*Catenulispora*	0.0013	0.0018	0.0421
Bacteria	*Cyanobacteria*	*Chamaesiphon*	0.0035	0.0046	0.0380
Bacteria	*Proteobacteria*	*Collimonas*	0.0033	0.0041	0.0477
Bacteria	*Actinobacteria*	*Conexibacter*	0.0017	0.0023	0.0472
Bacteria	*Actinobacteria*	*Cryptobacterium*	0.0014	0.0018	0.0157
Bacteria	*Firmicutes*	*Dehalobacter*	0.0039	0.0047	0.0363
Bacteria	*Actinobacteria*	*Denitrobacterium*	0.0283	0.0388	0.0234
Bacteria	*Actinobacteria*	*Eggerthella*	0.0228	0.0275	0.0423
Bacteria	*Actinobacteria*	*Frankia*	0.0045	0.0058	0.0472
Bacteria	*Firmicutes*	*Geobacillus*	0.0039	0.0049	0.0234
Bacteria	*Actinobacteria*	*Gordonibacter*	0.0200	0.0238	0.0487
Archaea	*Euryarchaeota*	*Halococcus*	0.0013	0.0017	0.0478
Archaea	*Euryarchaeota*	*Haloferax*	0.0016	0.0022	0.0331
Archaea	*Euryarchaeota*	*Halorubrum*	0.0026	0.0034	0.0310
Bacteria	*Proteobacteria*	*Hoeflea*	0.0012	0.0016	0.0351
Bacteria	*Actinobacteria*	*Isoptericola*	0.0016	0.0022	0.0320
Bacteria	*Firmicutes*	*Jeotgalibacillus*	0.0010	0.0012	0.0132
Bacteria	*Actinobacteria*	*Kineococcus*	0.0010	0.0013	0.0406
Bacteria	*Kiritimatiellaeota*	*Kiritimatiella*	0.0034	0.0052	0.0312
Bacteria	*Actinobacteria*	*Kutzneria*	0.0009	0.0012	0.0443
Archaea	*Euryarchaeota*	*Methanobacterium*	0.0052	0.0072	0.0180
Archaea	*Euryarchaeota*	*Methanobrevibacter*	5.4253	7.2033	0.0459
Archaea	*Euryarchaeota*	*Methanothermus*	0.0008	0.0012	0.0138
Bacteria	*Cyanobacteria*	*Microcystis*	0.0035	0.0048	0.0402
Protist	*Ochrophyta*	*Nannochloropsis*	0.0168	0.0317	0.0249
Archaea	*Halobacteria*	*Natrialba*	0.0011	0.0015	0.0494
Bacteria	*Proteobacteria*	*Nitrosomonas*	0.0022	0.0027	0.0475
Bacteria	*Actinobacteria*	*Rubrobacter*	0.0019	0.0025	0.0432
Bacteria	*Actinobacteria*	*Saccharothrix*	0.0010	0.0013	0.0296
Bacteria	*Proteobacteria*	*Sorangium*	0.0067	0.0091	0.0462
Bacteria	*Actinobacteria*	*Streptosporangium*	0.0013	0.0018	0.0292
Bacteria	*Actinobacteria*	*Verrucosispora*	0.0010	0.0013	0.0332
Comparison between Low- compared to High-SFA groups for the 8*8 extreme animals					
Bacteria	*Actinobacteria*	*Adlercreutzia*	0.0132	0.0193	0.0428
Fungi	*Basidiomycota*	*Anthracocystis*	0.0027	0.0038	0.0346
Bacteria	*Actinobacteria*	*Cryptobacterium*	0.0014	0.0021	0.0379
Fungi	*Basidiomycota*	*Cutaneotrichosporon*	0.0663	0.0944	0.0345
Fungi	*Ascomycota*	*Cyphellophora*	0.0021	0.0031	0.0330
Bacteria	*Actinobacteria*	*Denitrobacterium*	0.0234	0.0470	0.0013
Bacteria	*Actinobacteria*	*Eggerthella*	0.0231	0.0321	0.0489
Bacteria	*Actinobacteria*	*Gordonibacter*	0.0199	0.0276	0.0425
Bacteria	*Proteobacteria*	*Hafnia*	0.0007	0.0015	0.0295
Bacteria	*Firmicutes*	*Jeotgalibacillus*	0.0010	0.0012	0.0438
Bacteria	*Proteobacteria*	*Komagataeibacter*	0.0020	0.0040	0.0451
Bacteria	*Proteobacteria*	*Kozakia*	0.0007	0.0016	0.0316
Bacteria	*Firmicutes*	*Lactobacillus*	0.1162	0.2687	0.0328
Bacteria	*Firmicutes*	*Leuconostoc*	0.0111	0.0330	0.0273
Bacteria	*Actinobacteria*	*Mycobacterium*	0.0881	0.1305	0.0257
Fungi	*Basidiomycota*	*Punctularia*	0.0066	0.0094	0.0247
Fungi	*Basidiomycota*	*Rhodotorula*	0.0043	0.0065	0.0256
Bacteria	*Firmicutes*	*Weissella*	0.0038	0.0145	0.0286

### Correlations Between Microbial Communities and Milk Fatty Acid Composition

**Figure 2:**
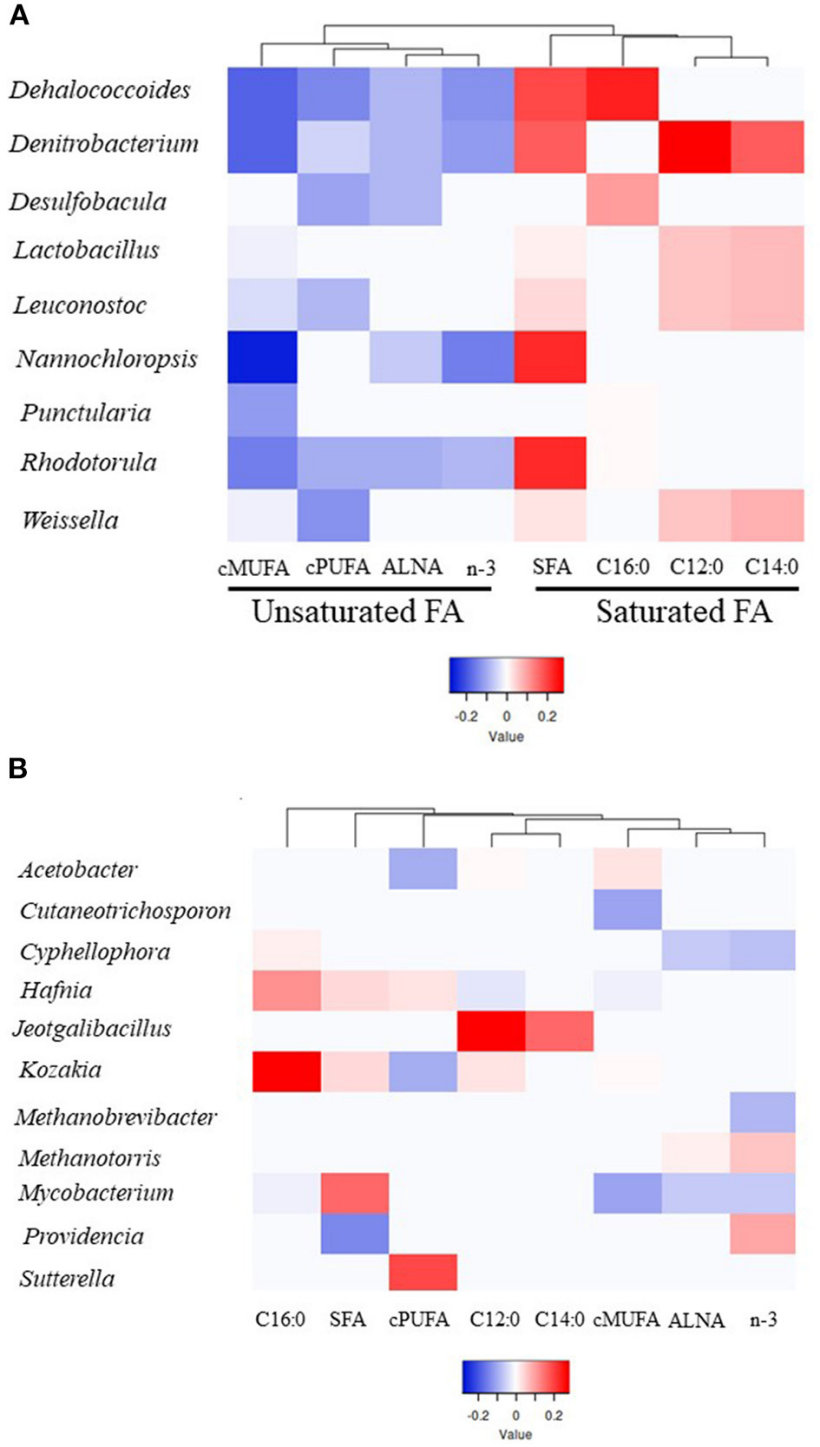
Heatmap of the microbial genera indicating their partial least square coefficient for each fatty acid. **(A)** Unsaturated or saturated FA groups correlated with a particular genus. **(B)** Individual FA groups correlated with a particular genus. Blue color indicates a negative PLS coefficient between the genus and the fatty acid, whilst red indicates the opposite.

Microbial genera explaining most of the variability (between 64 and 86%) for a particular group of FA was determined using PLS analysis (VIP > 0.8). Different profiles of genera were identified within SFA (total SFA, C12:0, C14:0 and C16:0) and UFA groups (ALNA, *cis-*MUFA, *cis-*PUFA and *n-*3) based on PLS coefficients with one main branch grouping saturated FA and the second branch unsaturated FA together (Figure 2A, Supplementary Figure 4, Supplementary Table 6). Within 69 genera identified as being important to explain the variability observed for FA groups, 9 genera including *Dehalococcoides, Denitrobacterium, Desulfobacula, Lactobacillus, Leuconostoc, Nannochloropsis, Punctularia, Rhodotorula*, and *Weissella* were all significantly positively correlated (positive PLS coefficient) with SFA groups and negatively correlated (negative PLS coefficient) with unsaturated FA groups (Figure 2A, Supplementary Figure 4, Supplementary Table 6). *Dehalococcoides, Desulfobacula, Punctularia*, and *Rhodotorula* were strongly positively correlated with C16:0 and in some cases with SFA whilst *Denitrobacterium*, and the LAB *Lactobacillus, Leuconostoc*, and *Weissella* were strongly positively correlated with C12:0, C14:0 and total SFA. *Dehalococcoides, Denitrobacterium*, and *Rhodotorula* were all negatively correlated with the unsaturated FA studied. The LAB *Leuconostoc* and *Weissella* were all negatively correlated with *cis-*MUFA and *cis-*PUFA whilst *Lactobacillus* only showed a negative correlation with *cis*-MUFA. The protist *Nannochloropsis* showed negative correlations with *cis-*MUFA and *n-*3 including ALNA, but a strong positive correlation with SFA (Figure 2A, Supplementary Figure 4, Supplementary Table 6).

Individual FA groups were correlated with a particular genus (Figure 2B, Supplementary Figure 4, Supplementary Table 6), such as *Providencia* (positive with *n-*3 and negative with SFA), *Jeotgalibacillus* (positive with C12:0 and C14:0), *Kozakia* (positive with C16:0 and negative with *cis-*PUFA), *Cutaneotrichosphoron* (negative with *cis-*MUFA), *Cyphellophora* (negative with ALNA and *n-*3 and positive with C16:0), and *Sutterella* (positive with *cis-*PUFA). *Hafnia* and *Acetobacter* genera showed contrasting correlation results within SFA or unsaturated FA. For example, *Hafnia* was positively correlated with C16:0 and *cis-*PUFA and negatively correlated with *cis-*MUFA. The two methanogen genera *Methanobrevibacter* and *Methanotorris* were negatively and positively correlated with *n-*3, respectively. Although *Mycobacterium* showed a positive correlation with SFA, this genus was negatively correlated with C16:0, *n-*3, ALNA, and *cis-*MUFA.

### Variation in the Abundance of Rumen Microbial Genes in Cows With Contrasting Milk SFA Content

From the 1,630 microbial genes with a relative abundance above 0.001%, 273 and 40 genes were significantly different (*P* < 0.05) between Low-SFA and High-SFA animals (48 animals) and also between extreme–low SFA and extreme-high SFA animals (16 animals), respectively (Supplementary Table 7). Within these two sets of genes, only 19 genes were shared between the 48 or the extreme animals with a majority of genes involved in translation, ribosomal structure and biogenesis (*n* = 6) or energy production and conversion (*n* = 4) but none directly related to FA biohydrogenation. Genes involved in methanogenesis, and encoding for formylmethanofuran dehydrogenase (K00200-204), methyl-coenzyme M reductase (K00399-402) and tetrahydromethanopterin S-methyltransferase (K00577, K00580, K00581, and K00584) were only significantly different when using the 48 animals.

In term of general functions (COG database), the enriched functions related to higher relative abundance of genes in extremes–low SFA compared to extreme-high SFA (Supplementary Figure 5), were carbohydrate transport and metabolism, intracellular trafficking, secretion, and vesicular transport and nucleotide transport and metabolism; mostly because of the genes involved in starch degradation (K00688), preprotein translocase synthesis (K03070), and genes encoding for adenylosuccinate synthase (K01939) and associated with purine metabolism. Although the general function group translation, ribosomal structure and biogenesis was enriched in extreme-low SFA animals, contrasting results were obtained depending on the genes constituting this group. For example, genes encoding for seryl-tRNA synthetase and GTP-binding protein LepA were significantly more abundant in extreme-low SFA animals and had a relative abundance above 0.1% whilst less abundant genes (<0.05%) were significantly more abundant in extreme-high-SFA animals and encoding for ribosomal subunit (K02956).

**Figure 3:**
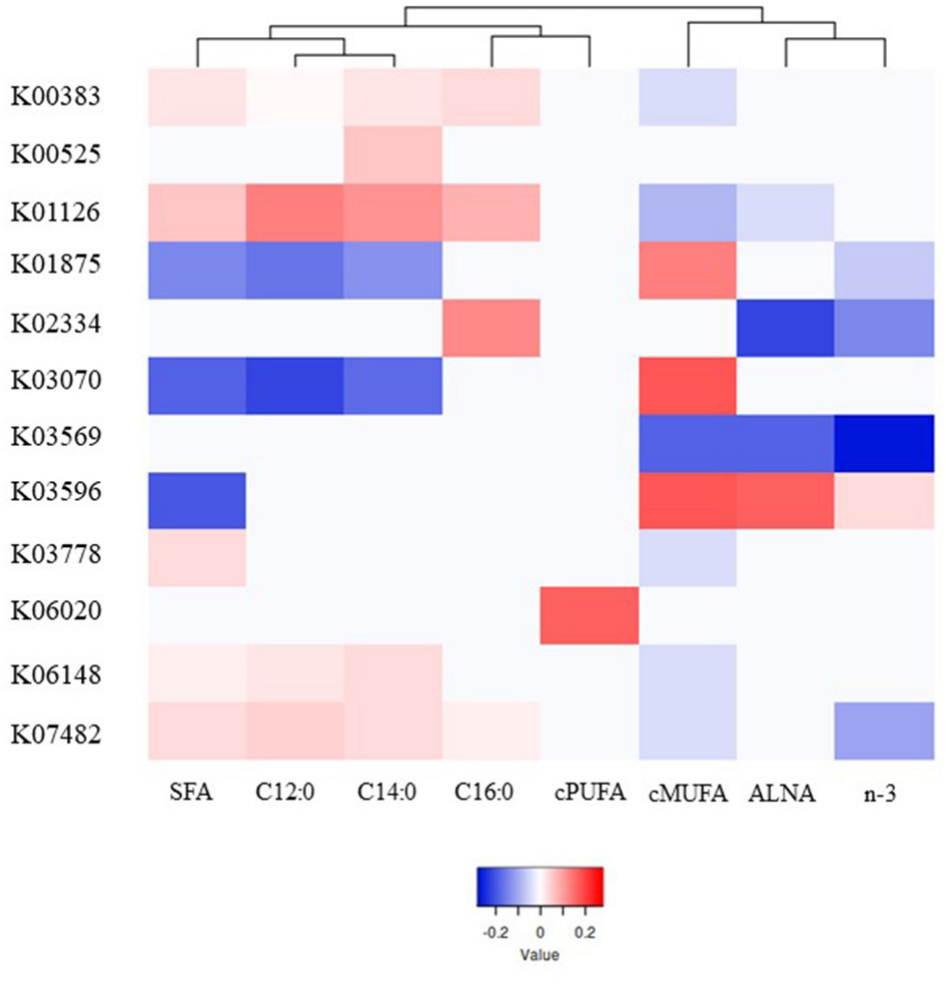
Heatmap of the most significant microbial genes indicating their partial least square coefficient for each fatty acid. Blue color indicates a negative PLS coefficient between the genus and the fatty acid, whilst red color indicates the opposite.

A total of 39 genes were highly correlated with FA groups (VIP > 0.8), explaining from 76% (C14:0) to 94% (C16:0) of the variability in milk FA by PLS analysis (Supplementary Table 8), and were associated with different pattern of responses as showed in the heatmap (Figure 3, Supplementary Figure 6). Contrasting with microbial genera, *cis-*PUFA did not group in the same main branch as other UFA. The genes which were significantly correlated with *cis-*PUFA by PLS were different for all FA tested and the gene with the highest VIP-value (>1.25) was involved in intracellular sulfate transport (K06020).

Within the 39 genes, the 8 genes which were positively correlated with SFA, and in general negatively correlated with unsaturated FA, were associated with stress resistance (K00383 and K07482), lactate metabolism (K03778), adaptation to new environments (K01126 and K06148), and cell division (K00525, K02334, and K03569). On the other hand, 3 other genes which are involved in no stress responses (K03070 and K03596) and protein synthesis (K01875) were strongly positively correlated mostly with *cis-*MUFA.

### Links Between the Rumen Microbial Genes and Rumen Metabolome

**Figure 4:**
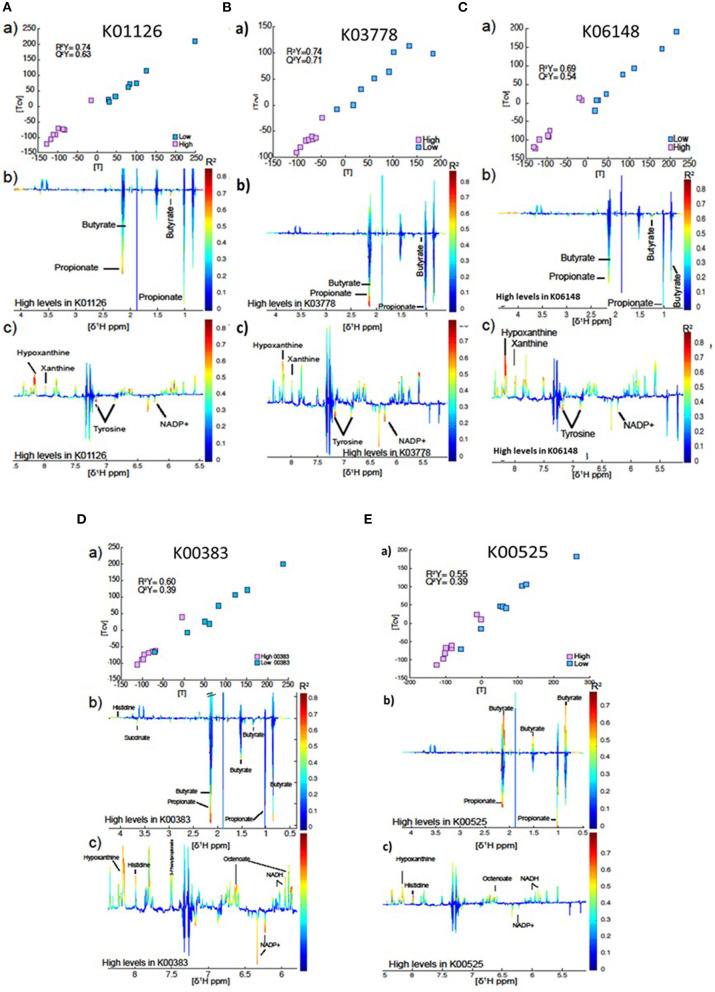
**(a)** Plot of the scores against the cross validated scores generated from the O-PLS DA model calculated using 16 samples of the spectra as a matrix of independent variables and high vs. low gene index as predictor. **(b,c)** Aliphatic and aromatic regions of ruminal fluid spectra showing metabolomics differences between groups with high and low levels of the genes K01126 **(A)**, K03778 **(B)**, K06148 **(C)**, K00383 **(D)**, and K00525 **(E)**.

In total, rumen microbial genes which were significantly correlated to milk SFA concentrations were used to compare the metabolome of cows that they had high vs. low (8 vs. 8) abundance of the gene (Figures 4A–E). The best models were obtained for the genes K01126 (*R*^2^Y = 0.74, *Q*^2^Y = 0.63) and K03778 (*R*^2^Y = 0.74, *Q*^2^Yc 0.71) and K06148 (*R*^2^Y = 0.69, *Q*^2^Y = 0.54). In all cases, higher levels of the gene were associated principally with butyrate, propionate, tyrosine and NADP+ production. Conversely, these individuals had lower levels of hypoxanthine and xanthine in the rumen fluid (see Figures 4A–C). The model produced by the gene K00383 showed the same pattern as the genes described above, but in this case the model was not as strong (R^2^Y = 0.60, Q^2^Y = 0.39, Figure 4D). Interestingly, higher levels of the gene K00525 were associated with high propionate, tyrosine and NADP+, but lower levels of butyrate (Figure 4E). However, this model was even weaker (R^2^Y = 0.55, Q^2^Y = 0.39).

Microorganisms strongly correlated with these 5 genes (VIP > 0.8) were mostly composed of the 3 LAB (*Lactobacillus, Leuconostoc*, and *Weissella*), as well as acetogenic bacteria such as *Acetobacter* and *Kozakia*, and to a lesser extend *Pediococcus* (LAB), *Komagataeibacter* (Acetogen), *Hafnia* (Phytate degrader) and other genera (*Babesia, Bacillus*, and *Oribacterium*). These genera explained between 67 and 88% of the variability of each gene using PLS analysis (Table 2) and the presence of these genes was also confirmed in their genome.

**Table 2:** Partial Least Square results identifying the most correlated genera with one of the genes identified as of interest explaining metabolomics results (K00383, K00525, K01126, K03778, and K06148).

**KEGG ID Gene K00383**		**Total variability explained^a^**=** 87.11%**	
VIP^*^	**Coefficient**	**Genus**	**Recognized activity**
1.32	0.20	*Weissella*	Lactic acid bacterium
1.29	0.19	*Leuconostoc*	Lactic acid bacterium
1.23	0.17	*Lactobacillus*	Lactic acid bacterium
1.13	0.12	*Acetobacter*	Acetogen
1.01	0.02	*Kozakia*	Acetogen
**KEGG ID Gene K00525**		**Total variability explained**^**a**^ **=** **73.96%**	
VIP^*^	**Coefficient**	**Genus**	**Recognized activity**
1.23	0.14	*Lactobacillus*	Lactic acid bacterium
1.22	0.15	*Leuconostoc*	Lactic acid bacterium
1.21	0.15	*Weissella*	Lactic acid bacterium
1.13	0.17	*Oribacterium*	Tannin resistance
1.11	0.02	*Pediococcus*	Lactic acid bacterium
0.92	0.22	*Bacillus*	Plant degrader
**KEGG ID Gene K01126**		**Total variability explained**^**a**^ **=** **66.79%**	
VIP^*^	**Coefficient**	**Genus**	**Recognized activity**
1.21	0.46	*Anaerovibrio*	Lipolytic bacterium
1.02	0.13	*Lactobacillus*	Lactic acid bacterium
0.98	0.13	*Leuconostoc*	Lactic acid bacterium
0.95	0.11	*Weissella*	Lactic acid bacterium
0.94	0.15	*Oribacterium*	Tannin resistance
0.91	0.08	*Hafnia*	Phytate degrader
**KEGG ID Gene K03778**		**Total variability explained**^**a**^ **=** **74.54%**	
VIP^*^	**Coefficient**	**Genus**	**Recognized activity**
1.38	0.26	*Leuconostoc*	Lactic acid bacterium
1.29	0.23	*Weissella*	Lactic acid bacterium
1.27	0.22	*Lactobacillus*	Lactic acid bacterium
0.90	0.03	*Pediococcus*	Lactic acid bacterium
0.90	0.07	*Acetobacter*	Acetogen
0.83	0.14	*Oribacterium*	Tannin resistance
0.81	0.16	*Bacillus*	Plant degrader
**KEGG ID Gene K06148**		**Total variability explained**^**a**^ **=** **88.07%**	
VIP^*^	**Coefficient**	**Genus**	**Recognized activity**
1.47	0.15	*Acetobacter*	Acetogen
1.28	0.12	*Weissella*	Lactic acid bacterium
1.25	0.09	*Kozakia*	Acetogen
1.21	0.11	*Leuconostoc*	Lactic acid bacterium
1.10	0.09	*Lactobacillus*	Lactic acid bacterium
1.09	0.10	*Babesia*	Potential Pathogen
1.08	0.04	*Komagataeibacter*	Acetogen
1.06	0.08	*Bacillus*	Plant degrader

a *Percentage of variability of one gene explained by several genera was determined using two PLS factors during the analysis*.

* *variable importance for projection (VIP)*.

## Discussion

The present study is the first, to our knowledge, to describe rumen microbial communities and genes and their associated mechanisms (using metagenomics), as well as the abundance of rumen metabolites in cows carrying these genes (metabolomics), which explain variation in the main milk SFA and UFA, using data and samples from 48 dairy cows. The present work also increases the number of rumen microbiome and metagenome results for dairy cattle and provides further information on possible mechanisms impacting milk FA profile (Saleem et al., 2012; Pitta et al., 2016; Golder et al., 2018). Milk FA originate by direct transfer from the diet, *de novo* synthesis in the mammary gland (using VFA produced in the rumen as substrate), rumen microbial biohydrogenation of dietary FA, desaturase enzyme activity in the mammary gland, and release from body fat stores (when the cow is in negative energy balance) (Chilliard et al., 2007). Although this manuscript mainly focused on understanding the importance of the rumen microbiome to explain variation in milk FA profile in dairy cattle, host parameters such as host genetic variation, fat mobilization, volatile fatty acids (VFA) absorption across the rumen wall or mammary gland activities are also important to explain the variability on milk fat profile (e.g., *cis-*MUFA) (Bielak et al., 2016; Ibeagha-Awemu et al., 2016). However, the impact of the rumen bacterial community on odd chain FA and PUFA with 18 carbon atoms in milk was previously confirmed, and it was notably a better predictor than host genotype information in Holstein cattle (Buitenhuis et al., 2019). As further rumen metagenomic datasets become available, it is likely that the predictive power of such analyses will increase and the role of rumen microbiome and their genes on milk FA profile will further unravel (Stewart et al., 2019).

### The Relation Between Rumen Microbiome Communities and Milk Fatty Acid Profile

A number of rumen microorganisms, have been found at higher abundance in the rumen of cows that produced milk with higher concentrations of SFA and were also positively correlated to SFA, C12:0, C14:0, or C16:0 and/or negatively correlated to *cis-*MUFA, *cis-*PUFA or *n-*3 PUFA. These were mainly LAB (*Lactobacillus, Leuconoctoc*, and *Weissella*), Proteobacteria (*Acetobacter* and *Kozakia*) and Actinobacteria (*Mycobacterium*), fungi (*Cutaneotrichosporon, Cyphellophora*), and to a lesser extent (e.g., significant when the 48 cows were used in the analysis but not when the extreme 16 cows were used) protists (*Nannochloropsis*), and methanogens (*Methanobrevibacter*).

The abundance of LAB, especially *Lactobacillus*, is higher in animals fed high-grain diets, and associated with lower rumen pH, because of the intake of rapidly fermentable carbohydrates, such as starch (Oetzel, 2003; Cremonesi et al., 2018). LAB are natural inhabitants of the rumen (Oetzel, 2003; Chen et al., 2012) and in this study were more abundant in extreme High-SFA animals (showing positive and negative correlations on SFA and unsaturated FA, respectively). This may reveal a relationship between slight changes in rumen pH and microbiome adaptation to lower pH, which further impacts milk SFA profile; as microorganisms which explain most of the variation in milk SFA profile are also those which are favored by lower rumen pH. It is however still unclear what is the extend of pH reductions that can trigger such microbiome responses. *Lactobacillus* species and other LAB are currently proposed as probiotics by Pophaly et al. (2012) to enhance gastrointestinal health in dairy cattle (with contrasting effectiveness between studies) and further studies may be necessary to identify beneficial LAB, that improve gastrointestinal health but also maintain rumen pH homeostasis and milk nutritional quality (Davidson et al., 2008).

Proteobacteria (in particular *Acetobacter and Kozakia*), which were found at higher concentrations in the rumen of cows producing High-SFA milk, are known to be involved in acetate production (Kersters et al., 2006). Fungi are known to improve fiber fermentation of the feed mainly by complementing mastication effects to break down feed particles and open up the structure (Akin and Borneman, 1990), whilst fermentation of fibrous material (e.g., forage) enhances the synthesis of acetate, at expense of propionate in the rumen (McDonald et al., 2011). It is therefore likely that the higher abundance of acetate-producing bacteria, and also potentially to a lesser extent, fiber-fermenting fungi, increase acetate synthesis, absorption and transfer to mammary gland. Acetate is extensively used by the mammary gland for the *de novo* synthesis of milk fat via the cytosolic malonyl-CoA pathway, while the main source of acetate in the mammary gland is the acetate which is produced in the rumen and subsequently absorbed and circulated in blood (McDonald et al., 2011). Given that *de novo* fat synthesis in the mammary gland includes solely SFA (all milk SFA from C4:0-C14:0 and 50% of milk C16:0 concentrations) (Chilliard et al., 2007), it is likely that higher supply of acetate would increase milk SFA synthesis and secretion to milk.

Previous work has shown that methanogens may enhance the ability of fungi to ferment fiber and well shift electron use toward the transformation of lactate and ethanol to methane via hydrogen; which is in turn converts to methane and carbon dioxide (Mountfort et al., 1982); while an overall increase in acetate abundance in the rumen may also be enhanced by such increase in fiber fermentation process. In the present study, methanogens were correlated with high concentrations of milk SFA when the 24 high-SFA vs. 24 low-SFA cows were compared. This may reveal a complementary role that methanogens play in increasing synthesis of acetate, as shown above for Proteobacteria and fungi, and potentially also adapting to slight reductions in rumen pH, as shown above for LAB; which further supports the suggestion that milk SFA content relates to such microbial activities.

In addition, the fungi, *Cutaneotrichosporon* (more abundant in the rumen of cows producing less *cis-*MUFA) is a lipase producing yeast (Kuncharoen et al., 2020), which may therefore enhance the hydrolysation of ester linkages in lipids causing the breakdown of triacylglycerols into glycerol and free fatty acids. This process will subsequently increase the availability of free fatty acids for ruminal biohydrogenation, the transformation *cis-*PUFA to a great range of *trans-*MUFA and *trans-*PUFA, and the saturated FA C18:0, and eventually reduce the flow of *cis-*UFA in the small intestine for absorption (Jenkins et al., 2008). The higher concentrations of *Nannochloropsis* in the rumens of cows that produced High-SFA milk (when the 24 high-SFA vs. 24 low-SFA cows were compared) was rather surprising given that *Nannochloropsis* is known to synthesize and store high concentrations of long chain *n-3* PUFA in their triacylglycerol (Ma et al., 2016). It is likely that the negative correlation with milk *n-3* is a result of the increased production of pyruvate, via the carbohydrate catabolic activity of *Nannochloropsis* (Li et al., 2014), which is the source of VFA (including acetate) in the rumen.

As previously shown in beef cattle, a stress on the rumen microbiome, caused by low pH, also increases the relative abundance of pathogens (Auffret et al., 2017). In this study, abundances of *Mycobacterium* were significantly more abundant in High-SFA animals and several species within the *Mycobacterium* genus are obligatory or opportunistic human and animal pathogens (Rastogi et al., 2001). Therefore, cows producing milk with more SFA in this study were also increasingly exposed to internal pathogens; although cows in this work did not experience any health incidences, this finding highlights that health benefits (e.g., reduced risk of disease) and improvement of milk nutritional quality may be related.

### The Relation Between Rumen Microbial Genes and Milk Fatty Acid Profile

The findings in the present study support a strong correlation between rumen microbial genes and FA profile, as the composition of microbial genes explained from 76 to 94% of the variation in certain individual FA and FA groups. In this study, K00688, a gene involved in starch degradation was more abundant in extreme High-SFA animals (positive and negative effects on SFA and unsaturated FA, respectively). In addition, microbial genes explaining most of the variability in milk FA, included those associated with lactic acid metabolism (K03778), and genes related to stress resistance and environmental adaptation by the synthesis of glutathione (K00383), which were mainly carried in LAB. This was coupled with the higher abundance of the cofactor NADPH/NADP^+^ (which was also identified by metabolomics as significantly higher in cows carrying more of these genes), and cell wall modification (K01126) or ABC transporter (K06148), which are important for LAB survival in lower pH environments (Pophaly et al., 2012; Zehavi et al., 2018). Therefore, the findings further support that microbial genes that may increase the synthesis of lactate in the rumen as well as enhance the adaptation of rumen microbiome in reduced pH conditions are also highly associated with higher concentrations of SFA in milk. This is potentially due to the subsequently increased *de novo* synthesis of SFA in the mammary gland by using the additional acetate (Chilliard et al., 2007), as described above.

As an outcome of the potential pH adaptation and effective lactate metabolism by rumen microbes, the concentrations of lactic acid were not significantly different between animals that had high concentrations vs. low abundances of these genes. The main explanation may be that lactate is easily metabolized by the rumen microbiome to produce propionate during the digestion of carbohydrates; a process which is also positively correlated with rumen pH (Plaizier et al., 2008; Saleem et al., 2012). Similarly, acetate concentrations in the rumen were not significantly different between animals with extreme abundances in these genes, and this can be partly explained by the fact that the two acetogenic bacteria, *Acetobacter*, and *Kozakia*, were positively correlated with the contrasting groups of FA (SFA and *cis-*MUFA, respectively); although the mechanisms controlling acetate concentration in the rumen, an important source for milk FA synthesis, are still unclear (McDonald et al., 2011).

Although based on the discussion above, it would be expected that acetate concentrations would be higher in the rumen of cows that produced High-SFA milk or carrying more of the correlated microbial genes, this was not observed. However, there is generally a lack of significant relationship between acetate concentrations in rumen fluid and plasma (McDonald et al., 2011), thus revealing that higher concentrations in plasma and mammary gland (that would increase milk SFA synthesis) do not necessarily reflect higher concentrations in rumen fluid. In addition, the rumen microbial genes associated with High-SFA milk were also correlated with increased concentrations of butyrate and propionate, rather than acetate. Their higher concentrations further support the suggestion that cows producing High-SFA milk may undergo higher rates of carbohydrate fermentation, because butyrate and propionate also represent, together with acetate, the major end products of rumen carbohydrate digestion (McDonald et al., 2011). However, the extent of metabolism of acetate during absorption via the rumen wall is lower than that of butyrate and propionate (Bergman and Wolff, 1971; Kristensen et al., 2000) and therefore it is likely that the relative proportions of these VFA in the rumen does not necessarily reflect the relative proportions of these VFA in the blood and mammary gland.

Interestingly, genes identified as important to explain milk SFA variation were associated with lactic acid metabolism, environmental adaptation and stress resistance but not FA biohydrogenation. Ventto et al. (2017) suggested that milk FA profile was under the control of multiple mechanisms in the rumen and the mammary gland and contributing to the regulation of fat synthesis. All milk SFA with carbon chains between C4-14, and 50% of C16:0, originate from *de novo* synthesis in the mammary gland using VFA as a substrate, while stearic acid, the end-product of rumen hydrogenation of dietary lipids, would partly convert to oleic acid in the mammary gland under the influence of Δ^9^ desaturase (Chilliard et al., 2007). It therefore seems that rumen hydrogenation, which is expected to play vital role in the concentrations of unsaturated fatty acids which are direct intermediate products of it (Chilliard et al., 2007), is not necessarily the most influential pathway for changes in milk SFA content as their synthesis are largely dictated by the activity in the mammary gland, and the supply of VFA substrates in the mammary gland. It appears that the effort of the microbial population to adapt to lower pH conditions in cows consuming the same diet, relates with the abundance of microbial communities and some of their genes responsible for such adaptation; many of which are also associated with milk SFA content. This demonstrates a potential indirect effect of rumen pH on specific microbial taxa adapted to environments with lower pH and impacting through their metabolism on rumen metabolites, lipolysis and eventually milk SFA content. The same genes which were associated with butyrate, propionate and the purine metabolites xanthine and hypoxanthine were more abundant in cows that produced milk with lower concentrations of SFA and higher concentrations of *cis-*MUFA.

Although challenging to speculate on any metabolic associations based on the findings of the present work, xanthine and hypoxanthine are known to be of microbial origin and their higher concentrations in the rumen also represent activities related to adaptation mechanisms to rumen pH changes (Saleem et al., 2012). The potential role of these metabolites to support rumen pH homeostasis should be investigated in the future. In addition, the fact that that microbial genes correlated with higher concentrations of milk SFA were also negatively correlated with rumen concentrations of hypoxanthine and xanthine [related to protein metabolism, e.g., protein synthesis and conversion of inosinic acid to uric acid; (McDonald et al., 2011)], may indicate an indirect association between rumen protein metabolism and milk fatty acid profiles; which may be explored further in future.

Tyrosine and propionate (which are contributing to milk and protein synthesis) were identified by metabolomics as highly correlated with microbial genes which were abundant in High-SFA animals, but milk yield between Low-SFA and High-SFA animals was similar. Differences in microbial and liver metabolisms related to tyrosine and propionate could explain this lack of effect on milk yield, in contrast with previous results (Rae and Ingalls, 1984; Xue et al., 2018) but further work is needed to clarify these mechanisms.

To conclude, the present study revealed that some microbial taxa, genes and their activities within the rumen microbiome differ substantially between cows which, under the same diet and housing conditions, produce milk at the high range (66.9–74.4% total FA; 24 cows) than in the low range (60.2–66.6% total FA; 24 cows) of the nutritionally undesirable SFA concentrations; while simultaneously show higher concentrations of the nutritionally beneficial *cis-*MUFA, *cis-*PUFA and/or *n-*3 PUFA. Rumens of cows producing milk with more SFA were characterized by higher abundances of several lactic acid bacteria and acetogenic bacteria, *Mycobacterium* taxa, fungi, and at a lesser extent methanogens and protists. Rumen microbial genes positively correlated with higher concentrations of SFA in milk were associated with increased activity of adaptation to lower pH, by the presence of xanthine and hypoxanthine, and synthesis of acetate and butyrate (which increases their supply for saturated fatty acid synthesis in the mammary gland). The diversity and abundances of rumen microbial genes explained from 76% (for C14:0) to 94% (for C16:0) of the variation in milk fatty acid profiles, thus highlighting that the rumen microbial environment is highly correlated with milk nutritional quality.

## Data Availability Statement

Supplementary Material and Supporting Data for this article are uploaded to figshare and can be found at https://figshare.com/s/b5413ccaf25f9b0d9f87. Metagenomics raw data can be downloaded from the European Nucleotide Archive under accession PRJEB33080. A metadata file giving information on the animal experiment is associated to PRJEB33080 and also summarized in Supplementary Table 4.

## Ethics Statement

The animal study was reviewed and approved by the animal experiment was conducted at the Centre for Dairy Research (CEDAR), University of Reading, UK, all experimental procedures used were licensed, regulated, and inspected by the UK Home Office under the Animals (Scientific Procedures) Act, 1996.

## Author Contributions

MA and SS: conceptualization and project management. MA, IC-L, MM-O, MW, and RS: formal analysis. MA, MM-O, and SS: original writing. SS, IC-L, MM-O, RD, DH, RR, MW, and MA: review and editing. All authors read and approved the final manuscript.

## Conflict of Interest

The authors declare that the research was conducted in the absence of any commercial or financial relationships that could be construed as a potential conflict of interest.

## References

[B1] Agricultural and Food Research Council (1993). Energy and Protein Requirements of Ruminants. Wallingford, UK: CAB International.

[B2] Akin D. E. Borneman W. S. (1990). Role of rumen fungi in fiber degradation. J. Dairy Sci. 73, 3023–3032. 10.3168/jds.S0022-0302(90)78989-8 2178175

[B3] Aue W. P. Bartholdi E. Ernst R. R. (1975). Two-dimensional spectroscopy. application to nuclear magnetic resonance. J. Chem. Phys. 64:2229 10.1063/1.432450

[B4] Auffret M. D. Dewhurst R. J. Duthie C.-A. Rooke J. A. Wallace R. J. Freeman T. C. (2017). The rumen microbiome as a reservoir of antimicrobial resistance and pathogenicity genes is directly affected by diet in beef cattle. Microbiome 5:159 10.1186/s40168-017-0378-z 29228991 PMC5725880

[B5] Barcelo-Goblijn G. Murphy E. J. (2009). Alpha-linolenic acid and its conversion to longer chain n-3 fatty acids: benefits for human health and a role in maintaining tissue n-3 fatty acid levels. Prog. Lipid Res. 48, 355–374. 10.1016/j.plipres.2009.07.002 19619583

[B6] Bates B. Lennox A. Prentice A. Bates C. Page P. Nicholson S. (2014). National Diet and Nutrition Survey: Results From Years 1, 2, 3 and 4 Combined of the Rolling Program (2008/9–2011/12). London: Public Health England.

[B7] Bergman E. N. Wolff J. E. (1971). Metabolism of volatile fatty acids by liver and portal-drained viscera in sheep. Am. J. Physiol. 221, 586–592. 10.1152/ajplegacy.1971.221.2.586 5560311

[B8] Bielak A. Derno M. Tuchsherer A. Hammon H. M. Susenbeth A. Kuhla B. (2016). Body fat mobilization in early lactation influences methane production of dairy cows. Sci. Rep. 6:28135. 10.1038/srep28135 27306038 PMC4910095

[B9] Buitenhuis B. Lassen J. Noel S. J. Plichta D. R. Sørensen P. Difford G. F.. (2019). Impact of the rumen microbiome on milk fatty acid composition of Holstein cattle. Genet. Sel. Evol. 51:23. 10.1186/s12711-019-0464-8 31142263 PMC6542034

[B10] Butler G. Stergiadis S. Seal C. Eyre M. Leifert C. (2011). Fat composition of organic and conventional retail milk in northeast England. J. Dairy Sci. 94, 24–36. 10.3168/jds.2010-3331 21183013

[B11] Bylesjö M. Rantalainen M. Cloarec O. Nicholson J. K. Holmes E. Trygg J. (2006). OPLS discriminant analysis: combining the strengths of PLS-DA and SIMCA classification. J. Chemom. 20, 341–351. 10.1002/cem.1006

[B12] Chen Y. Oba M. Guan L. L. (2012). Variation of bacterial communities and expression of Toll-like receptor genes in the rumen of steers differing in susceptibility to subacute ruminal acidosis. Vet Microbiol. 159, 451–459. 10.1016/j.vetmic.2012.04.032 22622335

[B13] Chilliard Y. Glasser F. Ferlay A. Bernard L. Rouel J. Doreau M. (2007). Diet, rumen biohydrogenation and nutritional quality of cow and goat milk fat. Eur. J. Lipid Sci. Technol. 109, 828–855. 10.1002/ejlt.200700080

[B14] Chilliard Y. Martin C. Rouel J. Doreau M. (2009). Milk fatty acids in dairy cows fed whole crude linseed, extruded linseed, or linseed oil, and their relationship with methane output. J. Dairy Sci. 92, 5199–5211. 10.3168/jds.2009-2375 19762838

[B15] Claus S. P. Tsang T. M. Wang Y. Cloarec O. Skordi E. Martin F.-P.. (2008). Systemic multicompartmental effects of the gut microbiome on mouse metabolic phenotypes. Mol. Syst. Biol. 4:219. 10.1038/msb.2008.56 18854818 PMC2583082

[B16] Cloarec O. Dumas M.-E. Craig A. Barton R. H. Trygg J. Hudson J.. (2005). Statistical total correlation spectroscopy: an exploratory approach for latent biomarker identification from metabolic 1H NMR data sets. Anal. Chem. 77, 1282–1289. 10.1021/ac048630x 15732908

[B17] Cremonesi P. Conte G. Severgnini M. Turri F. Monni A. Capra E.. (2018). Evaluation of the effects of different diets on microbiome diversity and fatty acid composition of rumen liquor in dairy goat. Animal 12, 1856–1866. 10.1017/S1751731117003433 29306345

[B18] Davidson A. L. Dassa E. Orelle C. Chen J. (2008). Structure, function, and evolution of bacterial ATP-binding cassette systems. Microbiol. Mol. Biol. Rev. 72, 317–364. 10.1128/MMBR.00031-07 18535149 PMC2415747

[B19] Dieterle F. Ross A. Schlotterbeck G. Senn H. (2006). Probabilistic quotient normalization as robust method to account for dilution of complex biological mixtures. application in 1H NMR metabonomics. Anal. Chem. 78, 4281–4290. 10.1021/ac051632c 16808434

[B20] EFSA Panel on Dietetic Products Nutrition, and Allergies (NDA). (2010). Scientific Opinion on Dietary Reference Values for fats, including saturated fatty acids, polyunsaturated fatty acids, monounsaturated fatty acids, trans fatty acids, and cholesterol. EFSA J. 8:1461 10.2903/j.efsa.2010.1461

[B21] FAO (2008). Fats and fatty Acids in Human Nutrition - Report of an Expert Consultation. Geneva: FAO. 21812367

[B22] Ferlay A. Bernard L. Meynadier A. Malpuech-Brugère C. (2017). Production of trans and conjugated fatty acids in dairy ruminants and their putative effects on human health: a review. Biochimie 141, 107–120. 10.1016/j.biochi.2017.08.006 28804001

[B23] Ferris C. P. (2007). Sustainable pasture based dairy systems – meeting the challenges. Can. J. Plant Sci. 87, 723–738. 10.4141/CJPS06011

[B24] Firkins J. L. Yu Z. (2015). Ruminant nutrition symposium: how to use data on the rumen microbiome to improve our understanding of ruminant nutrition. J. Anim. Sci. 93, 1450–1470. 10.2527/jas.2014-8754 26020167

[B25] Givens D. I. (2008). Impact on CVD risk of modifying milk fat to decrease intake of saturated fatty acids and increase intake of cis-monounsaturates. Proc. Nutr. Soc. 67, 419–427. 10.1017/S0029665108008707 18847519

[B26] Givens D. I. (2010). Milk and meat in our diet: good or bad for health? Animal 4, 1941–1952. 10.1017/S1751731110001503 22445367

[B27] Glasser F. Ferlay A. Chilliard Y. (2008). Oilseed lipid supplements and fatty acid composition of cow milk: a meta-analysis. J. Dairy Sci. 91, 4687–4703. 10.3168/jds.2008-0987 19038946

[B28] Golder H. M. Thomson J. M. Denman S. E. McSweeney C. S. Lean I. J. (2018). Genetic markers are associated with the ruminal microbiome and metabolome in grain and sugar challenged dairy heifers. Front. Genet. 9:62. 10.3389/fgene.2018.00062 29535763 PMC5835139

[B29] Harfoot C. G. Hazlewood G. P. (1988). “Lipid metabolism in the rumen,” in The Rumen Microbial Ecosystem, ed P. N. Hobson (London: Elsevier Applied Science Publishers), 285–322.

[B30] Haug A. Hostmark A. T. Harstad O. M. (2007). Bovine milk in human nutrition. Lipids Health Dis. 6:25. 10.1186/1476-511X-6-25 17894873 PMC2039733

[B31] Henderson G. Cox F. Ganesh S. Jonker A. Young W. Global Rumen Census Collaborators.. (2015). Rumen microbial community composition varies with diet and host, but a core microbiome is found across a wide geographical range. Sci. Rep. 5:14567. 10.1038/srep14567 26449758 PMC4598811

[B32] Huws S. A. Scott M. B. Tweed J. K. Lee M. R. (2013). Fatty acid oxidation products ('green odour') released from perennial ryegrass following biotic and abiotic stress, potentially have antimicrobial properties against the rumen microbiota resulting in decreased biohydrogenation. J. Appl. Microbiol. 15, 1081–1090. 10.1111/jam.12314 23889811

[B33] Ibeagha-Awemu E. M. Peters S. O. Akwanji K. A. Imumorin I. G. Zhao X. (2016). High density genome wide genotyping-by-sequencing and association identifies common and low frequency SNPs, and novel candidate genes influencing cow milk traits. Sci. Rep. 6:31109. 10.1038/srep31109 27506634 PMC4979022

[B34] Jami E. White B. A. Mizrahi I. (2014). Potential role of the bovine rumen microbiome in modulating milk composition and feed efficiency. PLoS ONE 9:e85423. 10.1371/journal.pone.0085423 24465556 PMC3899005

[B35] Jenkins T. C. Wallace R. J. Moate P. J. Mosley E. E. (2008). Board-invited review: recent advances in biohydrogenation of unsaturated fatty acids within the rumen microbial ecosystem. J. Anim. Sci. 86, 397–412. 10.2527/jas.2007-0588 18042812

[B36] Karisa B. K. Thomson J. Wang Z. Stothard P. Moore S. S. Plastow G. S. (2013). Candidate genes and single nucleotide polymorphisms associated with variation in residual feed intake in beef cattle. Anim. Sci. 91, 3502–3513. 10.2527/jas.2012-6170 23736061

[B37] Kersters K. Lisdiyanti P. Komagata K. Swings J. (2006). “The family *Acetobacteraceae*: the genera Acetobacter, Acidomonas, Asaia, Gluconacetobacter, Gluconobacter, and Kozakia,” in The Prokaryotes, Vol. 5, eds Dworkin M. Falkow S. Rosenberg E. Schleifer K. H. Stackebrandt E. (New York, NY: Springer), 163–200.

[B38] Kliem K. E. Shingfield K. J. (2016). Manipulation of milk fatty acid composition in lactating cows: opportunities and challenges. Eur. J. Lipid Sci. Technol. 118, 1661–1683. 10.1002/ejlt.201400543

[B39] Kliem K. E. Shingfield K. J. Livingstone K. M. Givens D. I. (2013). Seasonal variation in the fatty acid composition of milk available at retail in the United Kingdom and implications for dietary intake. Food Chem. 141, 274–281. 10.1016/j.foodchem.2013.02.116 23768358

[B40] Kristensen N. B. Gäbel G. Pierzynowski S. G. Danfær A. (2000). Portal recovery of short-chain fatty acids infused into the temporarily-isolated and washed reticulo-rumen of sheep. Br. J. Nutr. 84, 477–482. 10.1017/S0007114500001781 11103218

[B41] Kuncharoen N. Techo S. Savarajara A. Tanasupawat S. (2020). Identification and lipolytic activity of yeasts isolated from foods and wastes. Mycology 11, 279–286. 10.1080/21501203.2020.1745922 33329923 PMC7723016

[B42] Li J. Han D. Wang D. Ning K. Jia J. Wei L.. (2014). Choreography of transcriptomes and lipidomes of Nannochloropsis reveals the mechanisms of oil synthesis in microalgae. Plant Cell 26, 1645–1665. 10.1105/tpc.113.121418 24692423 PMC4036577

[B43] Lourenco M. Ramos-Morales E. Wallace R. J. (2010). The role of microbes in rumen lipolysis and biohydrogenation and their manipulation. Animal 4, 1008–1023. 10.1017/S175173111000042X 22444606

[B44] Ma X.-N. Chen T.-P. Yang B. Liu J. Chen F. (2016). Lipid production from nannochloropsis. Mar. Drugs 14:61. 10.3390/md14040061 27023568 PMC4849066

[B45] McDonald P. Edwards R. A. Greenhalgh J. F. D. Morgan C. A. Sinclair L. A. Wilkinson R. (2011). Digestion in Animal Nutrition. 7th Edn. Harlow, UK: Pearson Education Limited.

[B46] Meiboom S. Gill D. (1958). Modified spin-echo method for measuring nuclear relaxation times. Rev. Sci. Instruments 29:688 10.1063/1.1716296

[B47] Mountfort D. O. Asher R. A. Bauchop T. (1982). Fermentation of cellulose to methane and carbon dioxide by a rumen anaerobic fungus in a triculture with *Methanobrevibacter* sp. strain RA1 and *Methanosarcina barkeri*. Appl. Environ. Microbiol. 44, 128–134. 10.1128/AEM.44.1.128-134.1982 16346048 PMC241979

[B48] Nettleton J. A. Brouwer I. A. Geleijnse J. M. Hornstra G. (2017). Saturated fat consumption and risk of coronary heart disease and ischemic stroke: a science update. Ann. Nutr. Metab. 70, 26–33. 10.1159/000455681 28125802 PMC5475232

[B49] Oetzel G. R. (2003). Subacute ruminal acidosis in dairy cattle. Adv. Dairy Technol. 15, 307–317.

[B50] Petri R. M. Schwaiger T. Penner G. B. Beauchemin K. A. Forster R. J. (2013). Characterization of the core rumen microbiome in cattle during transition from forage to concentrate as well as during and after an acidotic challenge. PLoS ONE 8:e83424. 10.1371/journal.pone.0083424 24391765 PMC3877040

[B51] Pitta D. W. Indugu N. Kumar S. Vecchiarelli B. Sinha R. Baker L. D.. (2016). Metagenomic assessment of the functional potential of the rumen microbiome in Holstein dairy cows. Anaerobe 38, 50–60. 10.1016/j.anaerobe.2015.12.003 26700882

[B52] Plaizier J. C. Krause D. O. Gozho G. N. McBride B. W. (2008). Subacute ruminal acidosis in dairy cows: the physiological causes, incidence and consequences. Vet. J. 176, 21–31. 10.1016/j.tvjl.2007.12.016 18329918

[B53] Pophaly S. D. Singh R. Pophaly S. D. Kaushik J. K. Tomar S. K. (2012). Current status and emerging role of glutathione in food grade lactic acid bacteria. Microb. Cell Fact. 11:114. 10.1186/1475-2859-11-114 22920585 PMC3462692

[B54] Rae R. C. Ingalls J. R. (1984). Lactational response of dairy cows to oral administration of L-tyrosine. J. Dairy Sci. 67, 1430–1438. 10.3168/jds.S0022-0302(84)81458-7 6747048

[B55] Rastogi N. Legrand E. Sola C. (2001). The mycobacteria: an introduction to nomenclature and pathogenesis. Rev. Sci. Tech. 20, 21–54. 10.20506/rst.20.1.1265 11288513

[B56] Roehe R. Dewhurst R. J. Duthie C.-A. Rooke J. A. Mckain N. Ross D. W.. (2016). Bovine host genetic variation influences rumen microbial methane production with best selection criterion for low methane emitting and efficiently feed converting hosts based on metagenomic gene abundance. PLoS Genet. 12:e1005846. 10.1371/journal.pgen.1005846 26891056 PMC4758630

[B57] Rooke J. A. Wallace R. J. Duthie C.-A. McKain N. Motta de Souza S. Hyslop J. J.. (2014). Hydrogen and methane emissions from beef cattle and their rumen microbial community vary with diet, time after feeding and genotype. Br. J. Nutr. 112, 398–407. 10.1017/S0007114514000932 24780126

[B58] Russell J. B. (1998). The importance of pH in the regulation of ruminal acetate to propionate ratio and methane production *in vitro*. J. Dairy Sci. 81, 3222–3230. 10.3168/jds.S0022-0302(98)75886-2 9891267

[B59] SACN (2018). Draft Report: Saturated Fats and Health. London: Public Health England.

[B60] Saleem F. Ametaj B. N. Bouatra S. Mandal R. Zebeli Q. Dunn S. M.. (2012). A metabolomics approach to uncover the effects of grain diets on rumen health in dairy cows. J. Dairy Sci. 95, 6606–6623. 10.3168/jds.2012-5403 22959937

[B61] Saleem F. Bouatra S. Guo A. C. Psychogios N. Mandal R. Dunn S. M. (2013). The bovine ruminal fluid metabolome. Metabolomics 9, 360–378. 10.1007/s11306-012-0458-9

[B62] Snelling T. J. Auffret M. D. Duthie C.-A. Stewart R. D. Watson M. Dewhurst R. J. (2019). Temporal stability of the rumen microbiota in beef cattle, and response to diet and supplements. Anim. Microbiome 1:16 10.1186/s42523-019-0018-y 33499961 PMC7807515

[B63] Stergiadis S. Berlitz C. B. Hunt B. Garg S. Givens D. I. Kliem K. E. (2019). An update to the fatty acid profiles of bovine retail milk in the United Kingdom: implications for nutrition in different age and gender groups. Food Chem. 276, 218–230. 10.1016/j.foodchem.2018.09.165 30409587

[B64] Stergiadis S. Leifert C. Seal C. J. Eyre M. D. Larsen M. K. Slots T. (2015). A 2-year study on milk quality from three pasture-based dairy systems of contrasting production intensities in Wales. J. Agric. Sci. 153, 708–731. 10.1017/S0021859614000963

[B65] Stergiadis S. Leifert C. Seal C. J. Eyre M. D. Nielsen J. H. Larsen M. K.. (2012). Effect of feeding intensity and milking system on nutritionally relevant milk components in dairy farming systems in the north east of England. J. Agric. Food Chem. 60, 7270–7281. 10.1021/jf301053b 22737968

[B66] Stergiadis S. Leifert C. Seal C. J. Eyre M. D. Steinshamn H. Butler G. (2014). Improving the fatty acid profile of winter milk from housed cows with contrasting feeding regimes by oilseed supplementation. Food Chem. 164, 293–300. 10.1016/j.foodchem.2014.05.021 24996337

[B67] Stewart R. D. Auffret M. D. Warr A. Walker A. W. Roehe R. Watson M. (2019). Compendium of 4,941 rumen metagenome-assembled genomes for rumen microbiome biology and enzyme discovery. Nat. Biotechnol. 37, 953–961. 10.1038/s41587-019-0202-3 31375809 PMC6785717

[B68] Stewart R. D. Auffret M. D. Warr A. Wiser A. H. Press M. O. Langford K. W.. (2018). Assembly of 913 microbial genomes from metagenomic sequencing of the cow rumen. Nat. Commun. 9:870. 10.1038/s41467-018-03317-6 29491419 PMC5830445

[B69] Swanson D. Block R. Mousa S. (2012). Omega-3 fatty acids EPA and DHA: health benefits throughout life. Adv. Nutr. 3, 1–7. 10.3945/an.111.000893 22332096 PMC3262608

[B70] Thomas C. (2004). Feed into Milk: an Advisory Manual. Nottingham, UK: Nottingham University Press.

[B71] Ulberth F. Gabernig R. G. Schrammel F. (1999). Flame-ionization detector response to methyl, ethyl, propyl, and butyl esters of fatty acids. J. Am. Oil Chem. Soc. 76, 263–266. 10.1007/s11746-999-0228-7

[B72] Ventto L. Leskinen H. Kairenius P. Stefański T. Bayat A. R. Vilkki J.. (2017). Diet-induced milk fat depression is associated with alterations in ruminal biohydrogenation pathways and formation of novel fatty acid intermediates in lactating cows. Br. J. Nutr. 117, 364–376. 10.1017/S0007114517000010 28236814

[B73] Viant M. R. (2007). Revealing the metabolome of animal tissues using 1H nuclear magnetic resonance spectroscopy. Methods Mol. Biol. 358, 229–246. 10.1007/978-1-59745-244-1_13 17035689

[B74] Wallace R. J. Rooke J. A. Duthie C.-A. Hyslop J. J. Ross D. W. McKain N.. (2014). Archaeal abundance in postmortem ruminal digesta may help predict methane emissions from beef cattle. Sci. Rep. 4:5892. 10.1038/srep05892 25081098 PMC5376199

[B75] Wallace R. J. Rooke J. A. Mckain N. Duthie C.-A. Hyslop J. J. Ross D. W.. (2015). The rumen microbial metagenome associated with high methane production in cattle. BMC Genomics 16:839. 10.1186/s12864-015-2032-0 26494241 PMC4619255

[B76] Wold S. (1995). “PLS for multivariate linear modelling,” in Chemometric Methods in Molecular Design, ed H. Van de Waterbeemd (Weinheim: VCH Publishers), 195–218.

[B77] Wood D. E. Salzberg S. L. (2014). Kraken: ultrafast metagenomic sequence classification using exact alignments. Genome Biol. Evol. 15:R46. 10.1186/gb-2014-15-3-r46 24580807 PMC4053813

[B78] World Health Organization (WHO) (2003). Food and Agriculture Organization of the United Nations, Diet, Nutrition and the Prevention of Chronic Diseases 2003. Geneva: World Health Organization.

[B79] Xue M. Sun H. Wu X. Guan L. L. Liu J. (2018). Assessment of rumen microbiota from a large dairy cattle cohort reveals the pan and core bacteriomes contributing to varied phenotypes. Appl. Environ. Microbiol. 84, e00970–18. 10.1128/AEM.00970-18 30054362 PMC6146982

[B80] Zehavi T. Probst M. Mizrahi I. (2018). Insights into culturomics of the rumen microbiome. Front. Microbiol. 9:1999. 10.3389/fmicb.2018.01999 30210474 PMC6123358

