# Supplementary material for: Unravelling the Role of Rumen Microbial Communities, Genes, and Activities on Milk Fatty Acid Profile Using a Combination of Omics Approaches
Source: Front Microbiol. 2021 Jan 21;11:590441. doi: 10.3389/fmicb.2020.590441 (PMC7859430; doi:10.3389/fmicb.2020.590441)
Supplement: Supplementary file 1 [file Data_Sheet_1.PDF]

S1A. S1B. S1C.

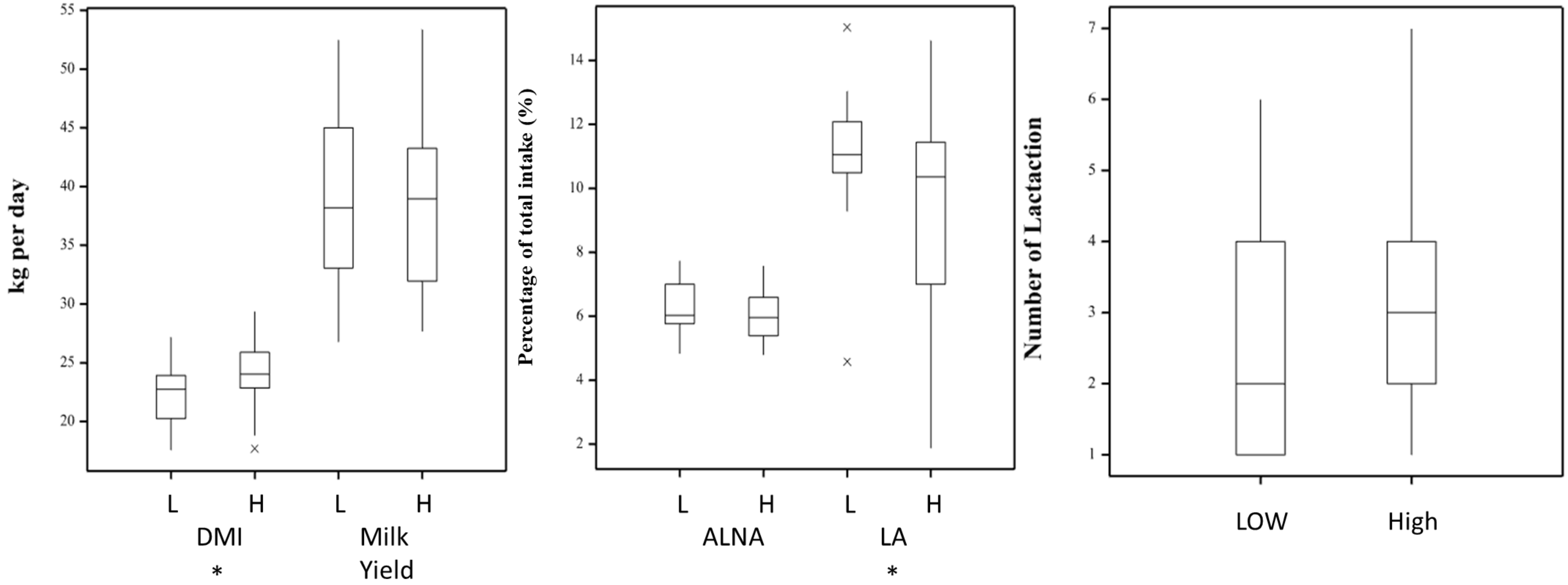
kg per day
55
50
45
40
35
30
25
20
L
H
L
H
DMI
*
Milk
Yield
Percentage of total intake (%)
14
12
10
8
6
4
2
L
H
L
H
ALNA
LA
*
Number of Lactaction
7
6
5
4
3
2
1
LOW
High

S2.

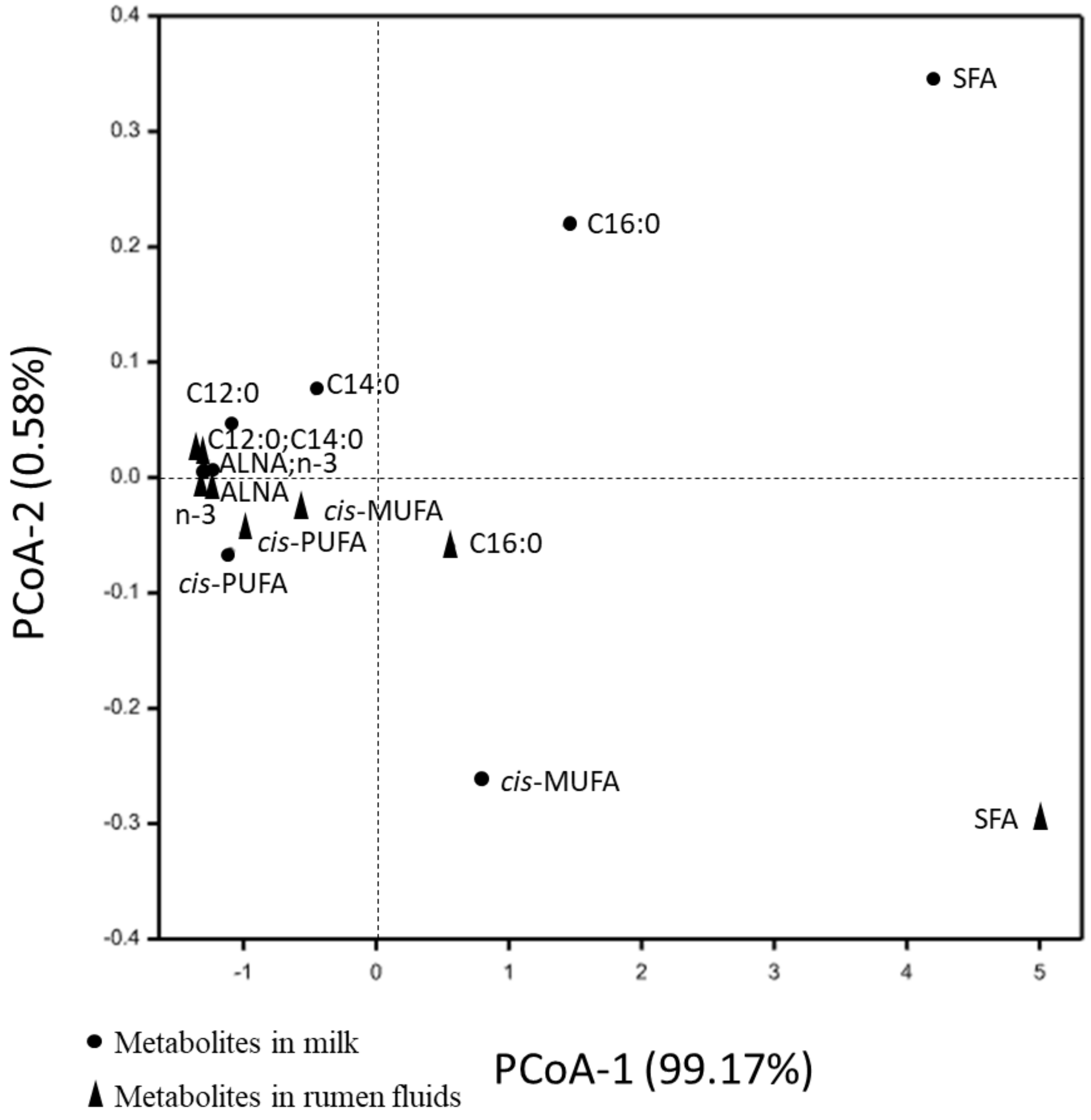

PCoA-2 (0.58%)
PCoA-1 (99.17%)
0.4
0.3
0.2
0.1
0.0
-0.1
-0.2
-0.3
-0.4
-1
0
1
2
3
4
5
SFA
C16:0
C14:0
C12:0
C12:0;C14:0
ALNA;n-3
ALNA
n-3
cis-MUFA
cis-PUFA
C16:0
cis-PUFA
cis-MUFA
SFA
Metabolites in milk
Metabolites in rumen fluids

S3A.

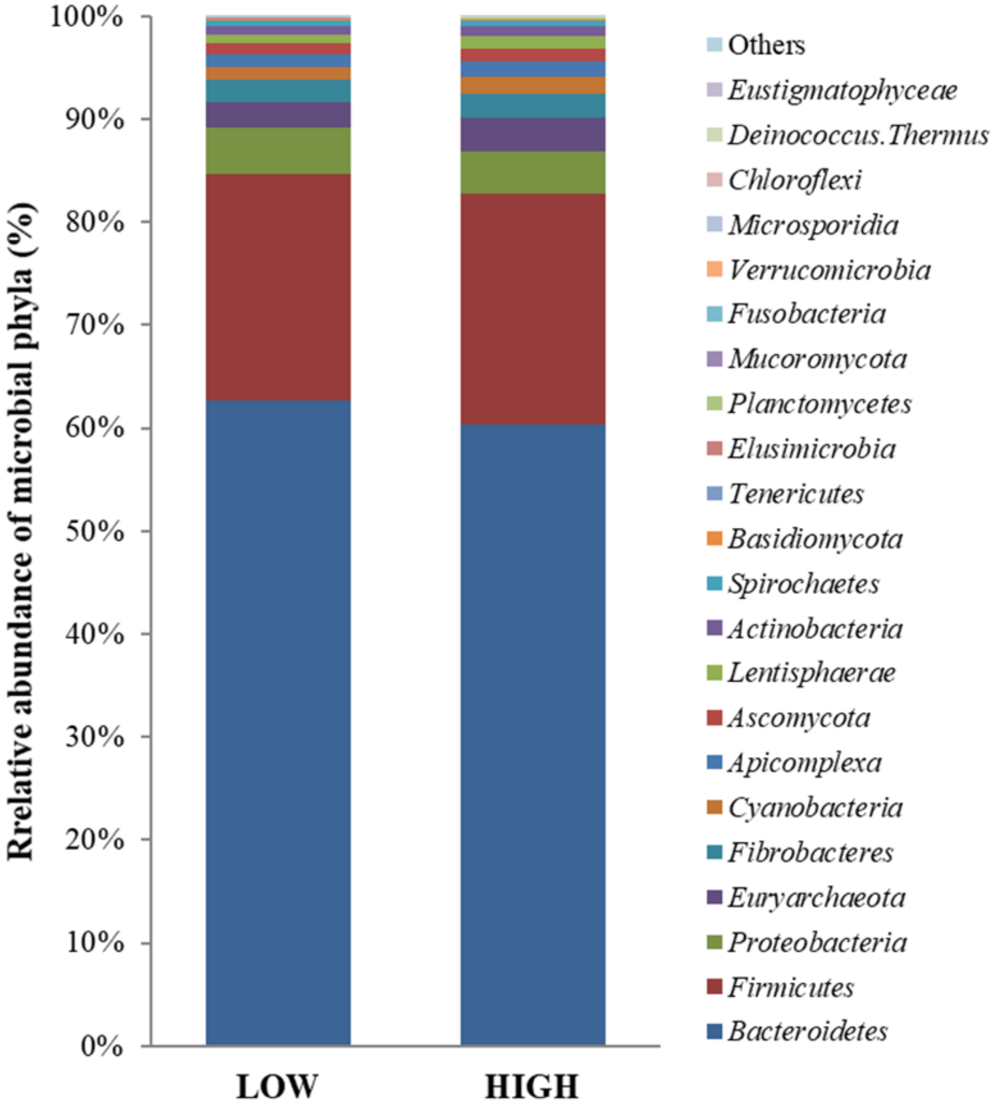

100%
90%
80%
70%
60%
50%
40%
30%
20%
10%
0%
Rrelative abundance of microbial phyla (%)
LOW
HIGH
Others
Eustigmatophyceae
Deinococcus.Thermus
Chloroflexi
Microsporidia
Verrucomicrobia
Fusobacteria
Mucoromycota
Planctomycetes
Elusimicrobia
Tenericutes
Basidiomycota
Spirochaetes
Actinobacteria
Lentisphaerae
Ascomycota
Apicomplexa
Cyanobacteria
Fibrobacteres
Euryarchaeota
Proteobacteria
Firmicutes
Bacteroidetes

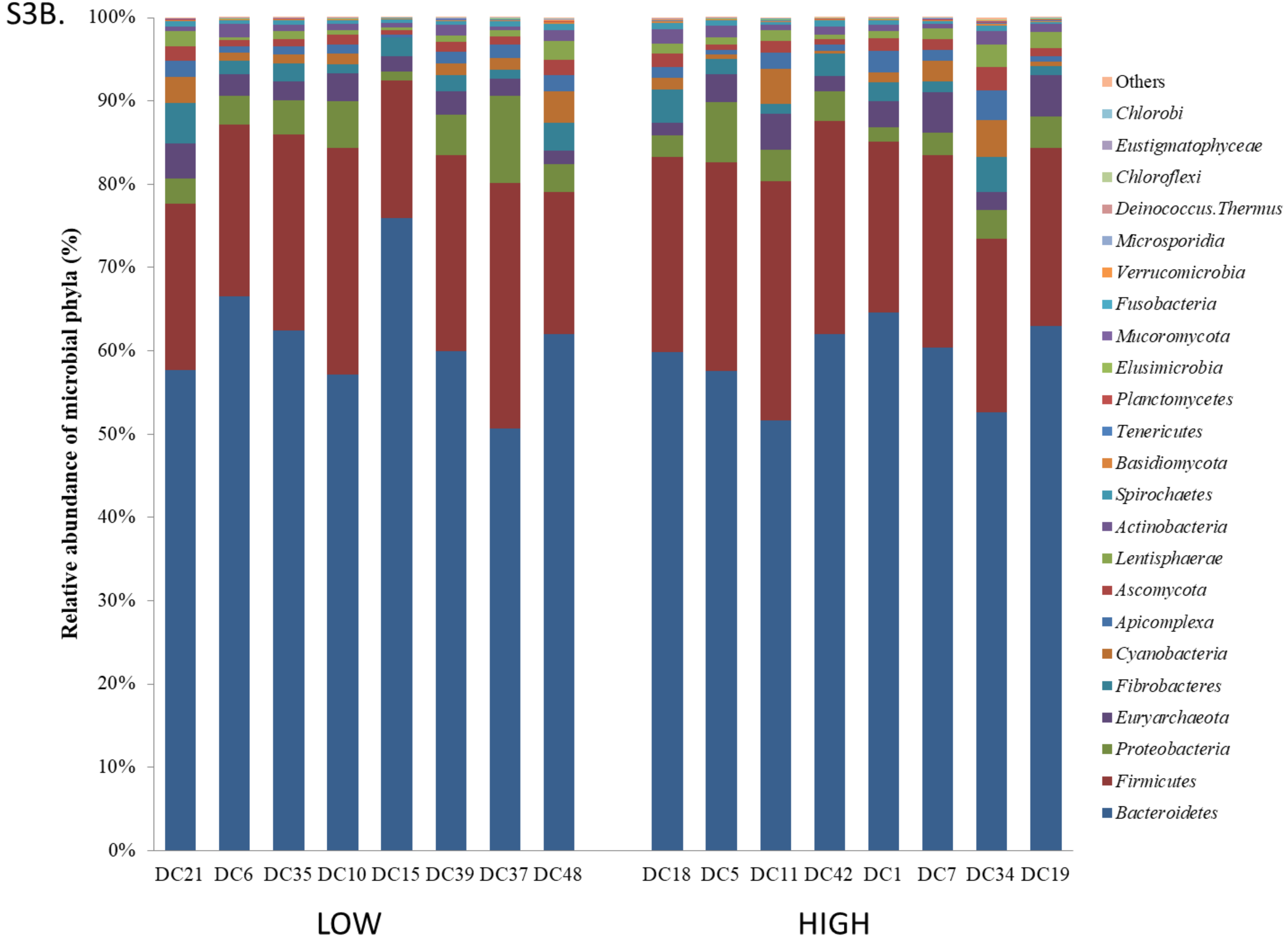
S3B.
Relative abundance of microbial phyla (%)
100%
90%
80%
70%
60%
50%
40%
30%
20%
10%
0%
DC21 DC6 DC35 DC10 DC15 DC39 DC37 DC48
DC18 DC5 DC11 DC42 DC1 DC7 DC34 DC19
LOW
HIGH
Others
Chlorobi
Eustigmatophyceae
Chloroflexi
Deinococcus.Thermus
Microsporidia
Verrucomicrobia
Fusobacteria
Mucoromycota
Elusimicrobia
Planctomycetes
Tenericutes
Basidiomycota
Spirochaetes
Actinobacteria
Lentisphaerae
Ascomycota
Apicomplexa
Cyanobacteria
Fibrobacteres
Euryarchaeota
Proteobacteria
Firmicutes
Bacteroidetes

S4.

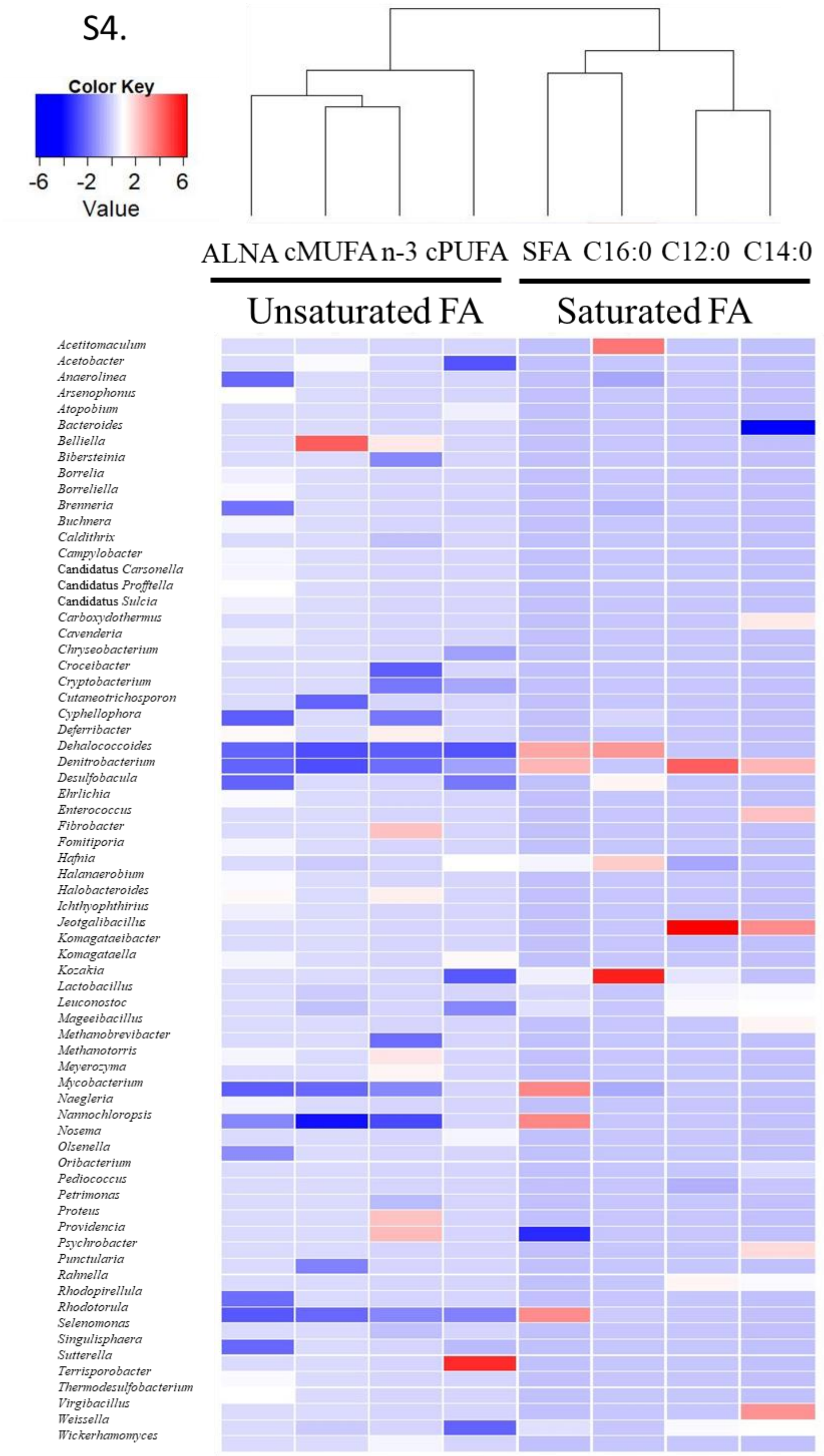
Color Key
-6 -2 2 6
Value
ALNA cMUFA n-3 cPUFA SFA C16:0 C12:0 C14:0
Unsaturated FA
Saturated FA
Acetitomaculum
Acetobacter
Anaerolinea
Arsenophonus
Atopobium
Bacteroides
Belliella
Bibersteinia
Borrelia
Borreliella
Brenneria
Buchnera
Caldithrix
Campylobacter
Candidatus Carsonella
Candidatus Profftella
Candidatus Sulcia
Carboxydothermus
Cavenderia
Chryseobacterium
Croceibacter
Cryptobacterium
Cutaneotrichosporon
Cyphellophora
Deferribacter
Dehalococcoides
Denitrobacterium
Desulfobacula
Ehrlichia
Enterococcus
Fibrobacter
Fomitiporia
Hafnia
Halanaerobium
Halobacteroides
Ichthyophthirius
Jeotgalibacillus
Komagataeibacter
Komagataella
Kozakia
Lactobacillus
Leuconostoc
Mageeibacillus
Methanobrevibacter
Methanotorris
Meyerozyma
Mycobacterium
Naegleria
Nannochloropsis
Nosema
Olsenella
Oribacterium
Pediococcus
Petrimonas
Proteus
Providencia
Psychrobacter
Punctularia
Rahnella
Rhodopirellula
Rhodotorula
Selenomonas
Singulisphaera
Sutterella
Terrisporobacter
Thermodesulfobacterium
Virgibacillus
Weissella
Wickerhamomyces

S5.

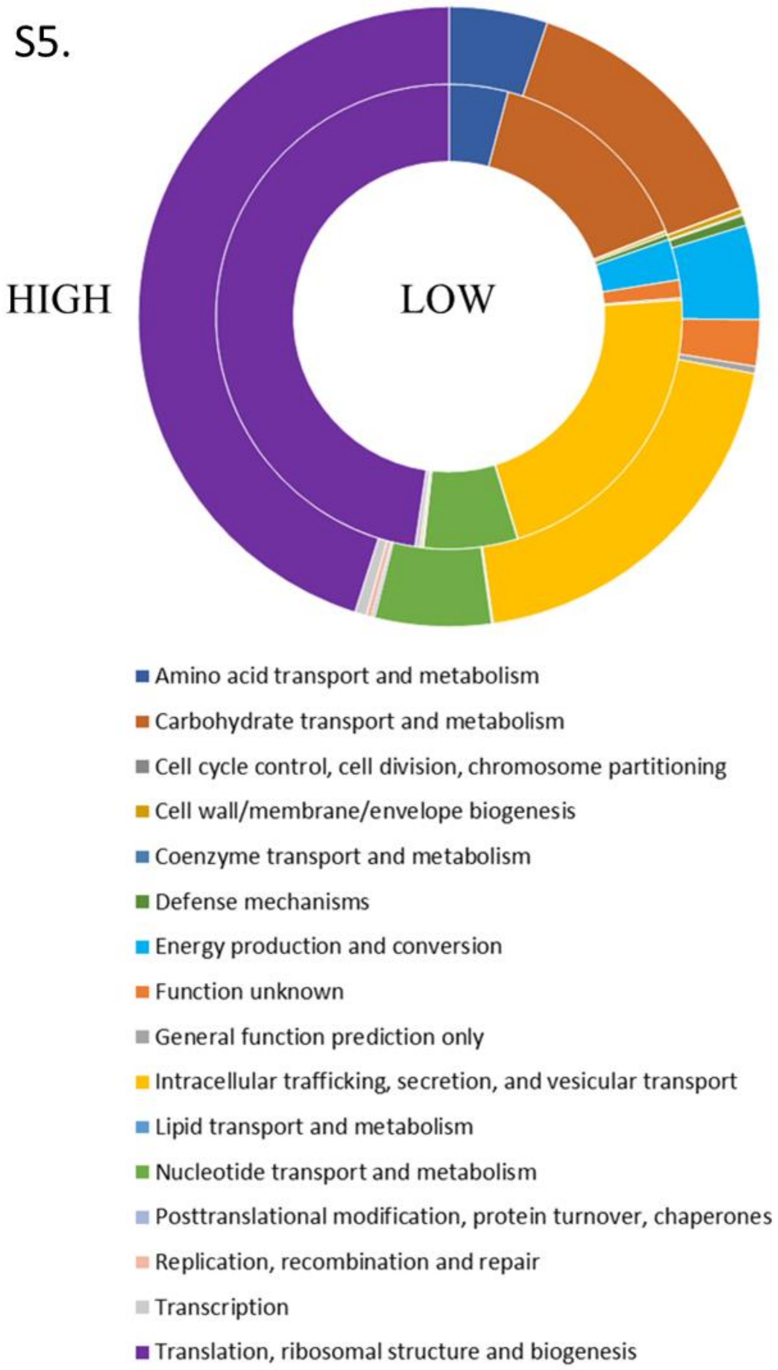
HIGH
LOW
Amino acid transport and metabolism
Carbohydrate transport and metabolism
Cell cycle control, cell division, chromosome partitioning
Cell wall/membrane/envelope biogenesis
Coenzyme transport and metabolism
Defense mechanisms
Energy production and conversion
Function unknown
General function prediction only
Intracellular trafficking, secretion, and vesicular transport
Lipid transport and metabolism
Nucleotide transport and metabolism
Posttranslational modification, protein turnover, chaperones
Replication, recombination and repair
Transcription
Translation, ribosomal structure and biogenesis

S6.

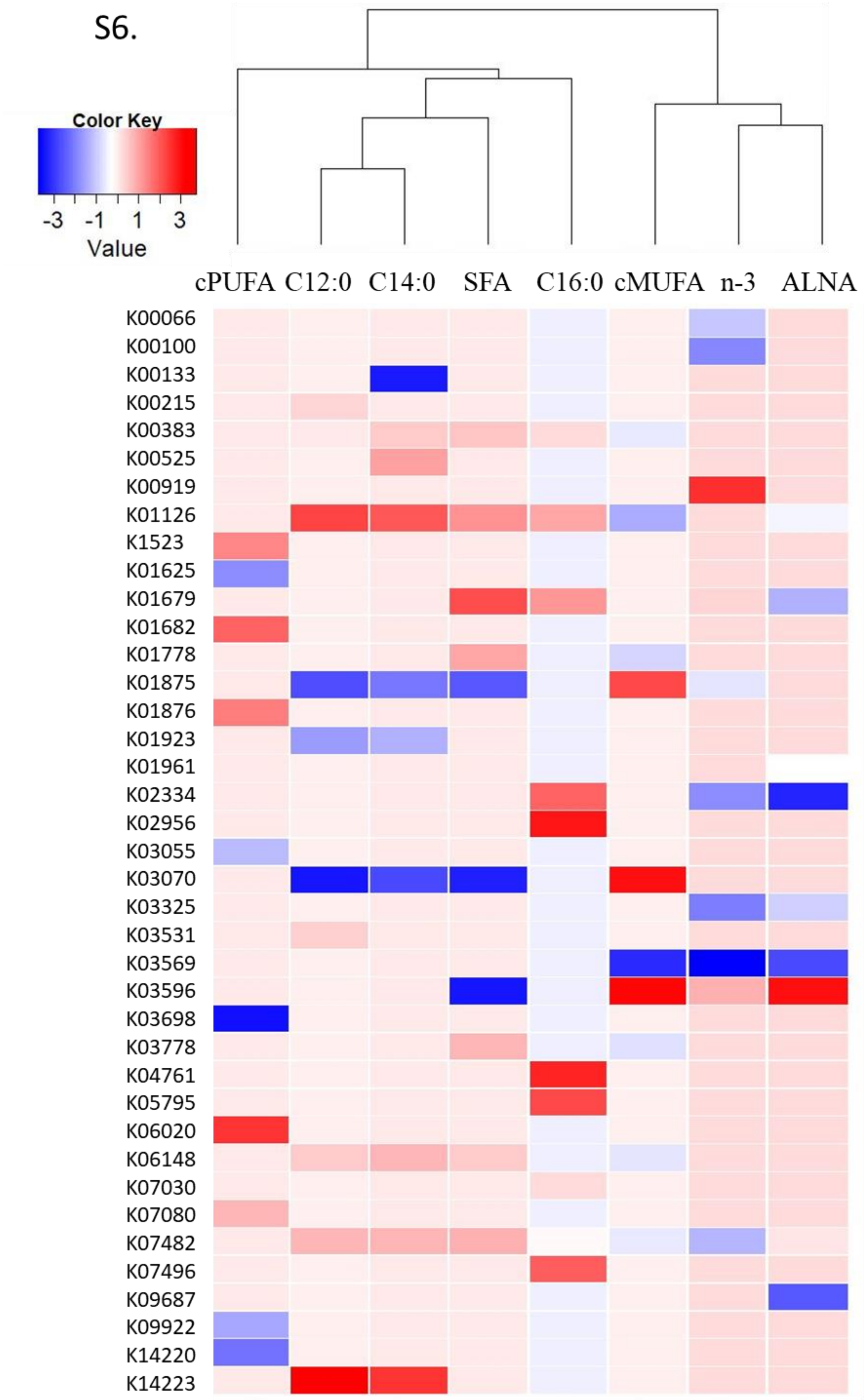
Color Key
-3 -1 1 3
Value
cPUFA C12:0 C14:0 SFA C16:0 cMUFA n-3 ALNA
K00066
K00100
K00133
K00215
K00383
K00525
K00919
K01126
K1523
K01625
K01679
K01682
K01778
K01875
K01876
K01923
K01961
K02334
K02956
K03055
K03070
K03325
K03531
K03569
K03596
K03698
K03778
K04761
K05795
K06020
K06148
K07030
K07080
K07482
K07496
K09687
K09922
K14220
K14223
