# Supplementary material for: Unravelling the Role of Rumen Microbial Communities, Genes, and Activities on Milk Fatty Acid Profile Using a Combination of Omics Approaches
Source: Front Microbiol. 2021 Jan 21;11:590441. doi: 10.3389/fmicb.2020.590441 (PMC7859430; doi:10.3389/fmicb.2020.590441)
Supplement: Supplementary file 2 [file Data_Sheet_2.docx]

**Unravelling the role of rumen microbial communities, genes and activities on milk fatty acid profile using a combination of omics approaches**

**Sokratis Stergiadis**^1†*^**, Irene Cabeza-Luna**^1,2^**, Marina Mora-Ortiz**^1‡^**, Robert D. Stewart^3^ Richard J. Dewhurst**^2^**, David J. Humphries**^4^**, Mick Watson**^3^**, Rainer Roehe**^2^, **Marc D. Auffret**^2†*€^

* Corresponding authors

^†^These authors contributed equally to this work

[s.stergiadis@reading.ac.uk](mailto:s.stergiadis@reading.ac.uk); [irenecl85@gmail.com](mailto:irenecl85@gmail.com) ; [Marina.mora_ortiz@kcl.ac.uk](mailto:Marina.mora_ortiz@kcl.ac.uk) ; [rob.stewart@ed.ac.uk](mailto:rob.stewart@ed.ac.uk); [Richard.Dewhurst@sruc.ac.uk](mailto:Richard.Dewhurst@sruc.ac.uk); [d.j.humphries@reading.ac.uk](mailto:d.j.humphries@reading.ac.uk); [Mick.watson@roslin.ed.ac.uk](mailto:Mick.watson@roslin.ed.ac.uk); [Rainer.Roehe@sruc.ac.uk](mailto:Rainer.Roehe@sruc.ac.uk); [Marc.Auffret@danone.com](mailto:Marc.Auffret@danone.com)

^1^University of Reading, Animal, Dairy and Food Chain Sciences, Reading, RG6 6AR, UK.

^2^SRUC, Roslin Institute Building, Edinburgh EH25 9RG, UK.

^3^The Roslin Institute, University of Edinburgh, Roslin Institute Building, Easter Bush, EH25 9RG, UK.

^4^University of Reading, Centre for Dairy Research, Reading, RG6 6AR.

‡Current address: Department of Twin Research, Kings College London, St Thomas’ Hospital Campus, 3rd Floor South Wing Block D Westminster Bridge Road, London SE1 7EH

^€^Current address: Danone Nutricia Research, Innovation, Science & Nutrition Department, Gut & Microbiology Laboratories, RD 128 Avenue de la Vauve, 91767 Palaiseau Cedex, France.

**Supplementary Tables**

**Figure captions**

Figure S1: Boxplots showing the variation in dry matter intake (DMI; kg per day; Fig. S1A), milk yield (kg per day; Fig. S1A), linoleic acid (LA) and α–linoleic acid (ALNA) transfer rate (Fig. S1B) and number of lactation (Fig. S1C) for 48 animals grouped between Low-(L) and High-(H) milk SFA. **P* < 0.05. The % of total intake of ALNA and LA was calculated as follow: g of ALNA/LA in milk/100 g ALNA/LA intake.

Figure S2: Principal Coordinate Analysis (PCoA) of microbial communities using genera data between 8 extreme Low-(grey circle) and High- (black circle) milk SFA groups.

Figure S3: Diversity of microbial phyla (relative abundance in %) for 48 animals grouped between Low-(LOW), medium-(MEDIUM) and High-(HIGH) milk SFA (Fig. S2A) and for the 8 extreme-LOW and 8 extreme-HIGH milk SFA (Fig. S2B).

Figure S4: Heatmap of the microbial genera indicating their partial least square coefficient for each fatty acid. Blue colour indicates a negative PLS coefficient between the genus and the fatty acid, whilst red indicates the opposite.

Figure S5: Doughnut charts showing the contribution of general functions (n = 16) identified using the Clusters of Orthologous Groups (COGs) classification and comparing predicted protein sequences of genes significant different between 8 extreme Low-(LOW) and High- HIGH) milk SFA groups with proteins of complete genomes in COGs database.

Figure S6: Heatmap of microbial genes indicating their partial least square coefficient for each fatty acid. Blue colour indicates a negative PLS coefficient between the genus and the fatty acid, whilst red colour indicates the opposite.

**Table S1.** Nutrient composition, digestibility, metabolisable energy contents, volatile compounds concentrations and fatty acid profile of the total mixed ratio fed to experimental cows.

|  | | | | |  |
| --- | --- | --- | --- | --- | --- |
|  | Mean | SD | Min | Max | |
| **Nutrient composition** |  |  |  |  | |
| DM (g/kg DM) | 402 | 16.5 | 391 | 421 | |
| Crude protein (g/kg DM) | 30.7 | 0.58 | 30.0 | 31.0 | |
| Oil (g/kg DM) | 21.4 | 1.31 | 20.0 | 22.6 | |
| Starch (g/kg DM) | 169 | 27.0 | 143 | 197 | |
| Neutral-detergent fibre (g/kg DM) | 132 | 4.7 | 128 | 137 | |
| Acid-detergent fibre (g/kg DM) | 70.0 | 2.82 | 67.9 | 73.2 | |
| Ash (g/kg DM) | 31.7 | 0.58 | 31.0 | 32.0 | |
| **Digestibility** |  |  |  |  | |
| DM digestibility (g/kg DM) | 767 | 25.2 | 740 | 790 | |
| **Energy contents** |  |  |  |  | |
| Metabolisable Energy (MJ/kg DM) | 12.1 | 0.2 | 11.8 | 12.2 | |
| **Volatile compounds** |  |  |  |  | |
| Lactic acid (g/kg DM) | 14.7 | 10.11 | 5.2 | 25.3 | |
| Ammonia N (g/kg DM) | 0.44 | 0.117 | 0.35 | 0.57 | |
| Ethanol (mg/kg DM) | 2920 | 166.6 | 2784 | 3106 | |
| Acetic acid (mg/kg DM) | 5473 | 591.1 | 4803 | 5922 | |
| Heptanoic Acid (mg/kg DM) | <25 |  |  |  | |
| Hexanoic Acid (mg/kg DM) | 126 | 33.1 | 97 | 162 | |
| iso-butyric Acid (mg/kg DM) | <25 |  |  |  | |
| iso-valeric Acid (mg/kg DM) | 49.0 | 28.16 | 28.0 | 81.0 | |
| n-butyric Acid (mg/kg DM) | 290 | 222.5 | 125 | 543 | |
| n-valeric Acid (mg/kg DM) | 58.5 | 43.13 | 28.0 | 89.0 | |
| Propan-1-ol (mg/kg DM) | 402 | 153.7 | 225 | 497 | |
| Propane-1,2-diol (mg/kg DM) | 368 | 131.5 | 275 | 461 | |
| Propionic Acid (mg/kg DM) | 252 | 108.6 | 131 | 341 | |
| **pH** | 4.53 | 0.058 | 4.50 | 4.60 | |
| **Fatty acid profile (g/kg DM)** |  |  |  |  | |
| C16:0 | 11.3 | 0.98 | 10.3 | 12.6 | |
| C18:0 | 1.1 | 0.10 | 1.0 | 1.2 | |
| C18:1 c9 | 9.9 | 0.62 | 9.4 | 10.9 | |
| C18:2 c9c12 | 11.5 | 0.32 | 11.2 | 11.9 | |
| C18:3 c9,12,15 | 4.5 | 0.47 | 3.8 | 4.9 | |
| Total fatty acids | 43.6 | 3.54 | 39.9 | 48.0 | |
|  | | | | |  |

n = number of samples, SD = standard deviation, Min = minimum value observed, Max = maximum value observed, DM = dry matter

**Table S2.** Fatty acid profile of milk (n =48) for the cows with contrasting potential for milk SFA.

|  | Low-SFA (n = 24) | | | |  | High-SFA (n =24) | | | | |
| --- | --- | --- | --- | --- | --- | --- | --- | --- | --- | --- |
|  | Mean | SD | Min | Max |  | Mean | SD | Min | Max | |
| **Individual fatty acids** |  |  |  |  |  |  |  |  |  | |
| C4:0 | 2.200 | 0.258 | 1.672 | 2.715 |  | 2.135 | 0.233 | 1.618 | 2.575 | |
| C5:0 | 0.017 | 0.005 | 0.007 | 0.027 |  | 0.018 | 0.004 | 0.008 | 0.025 | |
| C6:0 | 1.561 | 0.121 | 1.343 | 1.813 |  | 1.675 | 0.129 | 1.447 | 1.960 | |
| C7:0 | 0.017 | 0.004 | 0.010 | 0.026 |  | 0.021 | 0.004 | 0.016 | 0.034 | |
| C8:0 | 0.973 | 0.083 | 0.818 | 1.163 |  | 1.101 | 0.062 | 1.016 | 1.242 | |
| C9:0 | 0.020 | 0.006 | 0.005 | 0.031 |  | 0.029 | 0.007 | 0.019 | 0.050 | |
| C10:0 | 2.289 | 0.225 | 1.815 | 2.748 |  | 2.746 | 0.206 | 2.378 | 3.294 | |
| C10:1 c9 | 0.221 | 0.036 | 0.157 | 0.284 |  | 0.242 | 0.030 | 0.187 | 0.311 | |
| C11:0 | 0.042 | 0.009 | 0.026 | 0.059 |  | 0.057 | 0.014 | 0.035 | 0.097 | |
| C12:0 | 2.769 | 0.295 | 2.149 | 3.248 |  | 3.356 | 0.346 | 2.869 | 4.523 | |
| C13:0 iso | 0.020 | 0.003 | 0.015 | 0.025 |  | 0.020 | 0.002 | 0.017 | 0.024 | |
| C13:0 anteiso | 0.005 | 0.002 | 0.000 | 0.009 |  | 0.005 | 0.002 | 0.001 | 0.008 | |
| C12:1 c9 | 0.065 | 0.014 | 0.038 | 0.089 |  | 0.077 | 0.014 | 0.059 | 0.109 | |
| C13:0 | 0.077 | 0.013 | 0.053 | 0.100 |  | 0.090 | 0.017 | 0.061 | 0.141 | |
| C14:0 iso | 0.070 | 0.014 | 0.057 | 0.115 |  | 0.071 | 0.012 | 0.057 | 0.108 | |
| C14:0 | 10.244 | 0.668 | 9.149 | 11.244 |  | 11.123 | 0.595 | 10.201 | 12.269 | |
| C14:1 t9 | 0.177 | 0.011 | 0.154 | 0.194 |  | 0.175 | 0.012 | 0.135 | 0.192 | |
| C15:0 anteiso | 0.372 | 0.022 | 0.335 | 0.436 |  | 0.348 | 0.034 | 0.242 | 0.404 | |
| C14:1 c9 | 0.839 | 0.175 | 0.544 | 1.200 |  | 0.835 | 0.144 | 0.558 | 1.122 | |
| C15:0 | 0.841 | 0.073 | 0.701 | 0.953 |  | 0.885 | 0.099 | 0.722 | 1.214 | |
| C15:1 c9 | nd |  |  |  |  | nd |  |  |  | |
| C16:0 iso | 0.210 | 0.038 | 0.161 | 0.317 |  | 0.210 | 0.027 | 0.158 | 0.260 | |
| C16:0 | 31.968 | 1.953 | 27.792 | 35.816 |  | 34.104 | 2.139 | 30.971 | 41.285 | |
| C16:1 c7-8 | 0.050 | 0.008 | 0.029 | 0.066 |  | 0.044 | 0.006 | 0.027 | 0.050 | |
| C16:1 t9 | 0.062 | 0.012 | 0.038 | 0.087 |  | 0.048 | 0.008 | 0.026 | 0.068 | |
| C17:0 iso | 0.207 | 0.027 | 0.160 | 0.285 |  | 0.171 | 0.016 | 0.142 | 0.219 | |
| C16:1 c9 | 1.326 | 0.260 | 0.438 | 1.876 |  | 1.259 | 0.212 | 0.808 | 1.581 | |
| C17:0 anteiso | 0.409 | 0.090 | 0.041 | 0.527 |  | 0.386 | 0.037 | 0.296 | 0.462 | |
| C16:1 c14 | 0.171 | 0.027 | 0.110 | 0.227 |  | 0.197 | 0.027 | 0.155 | 0.244 | |
| C17:0 | 0.446 | 0.033 | 0.409 | 0.537 |  | 0.427 | 0.022 | 0.359 | 0.464 | |
| C18:0 iso | 0.090 | 0.016 | 0.030 | 0.114 |  | 0.086 | 0.007 | 0.074 | 0.105 | |
| C17:1 c9 | 0.178 | 0.034 | 0.127 | 0.260 |  | 0.143 | 0.020 | 0.120 | 0.194 | |
| C18:0 | 9.429 | 1.095 | 6.434 | 10.803 |  | 9.976 | 0.766 | 8.268 | 11.372 | |
| C18:1 t4 | 0.024 | 0.005 | 0.013 | 0.034 |  | 0.025 | 0.004 | 0.019 | 0.038 | |
| C18:1 t5 | 0.017 | 0.010 | 0.002 | 0.053 |  | 0.016 | 0.006 | 0.002 | 0.026 | |
| C18:1 t6-8 | 0.369 | 0.056 | 0.277 | 0.512 |  | 0.338 | 0.049 | 0.234 | 0.481 | |
| C18:1 t9 | 0.304 | 0.045 | 0.244 | 0.415 |  | 0.260 | 0.042 | 0.193 | 0.377 | |
| C18:1 t10 | 0.480 | 0.098 | 0.315 | 0.717 |  | 0.373 | 0.127 | 0.175 | 0.637 | |
| C18:1 t11 | 1.185 | 0.196 | 0.821 | 1.655 |  | 1.042 | 0.149 | 0.776 | 1.362 | |
| C18:1 c6 + C18:1 t12 | nd |  |  |  |  | nd |  |  |  | |
| C18:1 c9 | 22.630 | 1.444 | 20.729 | 26.412 |  | 19.389 | 1.320 | 16.697 | 21.142 | |
| C18:1 t15 | 0.537 | 0.297 | 0.168 | 1.200 |  | 0.595 | 0.490 | 0.150 | 2.099 | |
| C18:1 c11 | 0.900 | 0.074 | 0.770 | 1.027 |  | 0.758 | 0.149 | 0.526 | 1.367 | |
| C18:1 c12 | 0.402 | 0.057 | 0.265 | 0.493 |  | 0.344 | 0.055 | 0.170 | 0.430 | |
| C18:1 c13 | 0.086 | 0.021 | 0.042 | 0.139 |  | 0.070 | 0.021 | 0.041 | 0.124 | |
| C18:1 t16 + C18:1 c14 | 0.407 | 0.041 | 0.327 | 0.476 |  | 0.374 | 0.041 | 0.260 | 0.437 | |
| C19:0 + C18:1 c15 | 0.131 | 0.019 | 0.093 | 0.171 |  | 0.125 | 0.022 | 0.080 | 0.153 | |
| C18:2 t9t12 | nd |  |  |  |  | nd |  |  |  | |
| C18:2 t11t15 | 0.063 | 0.008 | 0.045 | 0.082 |  | 0.064 | 0.009 | 0.044 | 0.082 | |
| C18:2 c9t13 | 0.242 | 0.043 | 0.185 | 0.376 |  | 0.191 | 0.035 | 0.125 | 0.247 | |
| C18:2 c10t14 | 0.124 | 0.032 | 0.083 | 0.215 |  | 0.116 | 0.022 | 0.070 | 0.180 | |
| C18:2 c9t14 | 0.139 | 0.031 | 0.036 | 0.207 |  | 0.112 | 0.023 | 0.066 | 0.150 | |
| C18:2 c9t12 | 0.068 | 0.010 | 0.036 | 0.084 |  | 0.062 | 0.011 | 0.041 | 0.079 | |
| C18:1 c16 | 0.039 | 0.007 | 0.026 | 0.052 |  | 0.032 | 0.007 | 0.020 | 0.042 | |
| C18:2 t11c15 | 0.108 | 0.022 | 0.074 | 0.164 |  | 0.088 | 0.014 | 0.052 | 0.120 | |
| C18:2 c9c12 | 2.280 | 0.367 | 1.078 | 2.767 |  | 1.695 | 0.597 | 0.380 | 2.464 | |
| C19:1 c10 + C18:2c15 | 0.122 | 0.010 | 0.104 | 0.147 |  | 0.110 | 0.013 | 0.081 | 0.134 | |
| C20:0 | 0.121 | 0.015 | 0.085 | 0.149 |  | 0.128 | 0.014 | 0.110 | 0.175 | |
| C18:3 c6c9c12 | 0.032 | 0.005 | 0.023 | 0.043 |  | 0.030 | 0.007 | 0.015 | 0.042 | |
| C18:3 c9c12c15 | 0.481 | 0.049 | 0.398 | 0.571 |  | 0.421 | 0.044 | 0.284 | 0.496 | |
| C11 C20:1 | 0.074 | 0.005 | 0.065 | 0.084 |  | 0.063 | 0.008 | 0.036 | 0.079 | |
| C18:2 c9t11 | 0.638 | 0.087 | 0.448 | 0.822 |  | 0.493 | 0.065 | 0.398 | 0.654 | |
| C20:2 c11c14 | 0.025 | 0.009 | 0.011 | 0.060 |  | 0.029 | 0.007 | 0.019 | 0.048 | |
| C22:0 | 0.039 | 0.011 | 0.019 | 0.066 |  | 0.047 | 0.036 | 0.027 | 0.212 | |
| C20:3 c8c11c14 | 0.158 | 0.034 | 0.095 | 0.240 |  | 0.153 | 0.027 | 0.100 | 0.221 | |
| C22:1 c13 | nd |  |  |  |  | nd |  |  |  | |
| C20:3 c11c14c17 | 0.013 | 0.003 | 0.008 | 0.025 |  | 0.012 | 0.002 | 0.008 | 0.017 | |
| C20:4 c5c8c11c14 | 0.140 | 0.016 | 0.107 | 0.170 |  | 0.135 | 0.045 | 0.075 | 0.326 | |
| C22:2 c13c16 | 0.027 | 0.010 | 0.017 | 0.068 |  | 0.023 | 0.003 | 0.017 | 0.030 | |
| C20:5 c5c8c11c14c17 | 0.046 | 0.030 | 0.023 | 0.170 |  | 0.041 | 0.022 | 0.026 | 0.130 | |
| C24:0 | 0.025 | 0.007 | 0.009 | 0.042 |  | 0.027 | 0.011 | 0.014 | 0.067 | |
| C22:3 c13c16c19 | 0.013 | 0.011 | 0.004 | 0.042 |  | 0.014 | 0.013 | 0.004 | 0.050 | |
| C22:4 c7c10c13c16 | 0.048 | 0.061 | 0.018 | 0.314 |  | 0.027 | 0.010 | 0.008 | 0.051 | |
| C22:5 c7c10c13c16c19 | 0.075 | 0.025 | 0.043 | 0.135 |  | 0.082 | 0.051 | 0.040 | 0.295 | |
| C22:6 c4c7c10c13c16c19 | 0.025 | 0.027 | 0.007 | 0.141 |  | 0.032 | 0.028 | 0.011 | 0.121 | |
| **Fatty acid Groups** |  |  |  |  |  |  |  |  |  | |
| SFA | 64.525 | 1.829 | 60.183 | 66.637 |  | 69.306 | 1.975 | 66.902 | 74.432 | |
| MUFA | 30.668 | 1.625 | 28.621 | 34.380 |  | 26.819 | 1.389 | 23.399 | 28.776 | |
| cis-MUFA | 27.259 | 1.466 | 25.217 | 31.002 |  | 23.715 | 1.467 | 20.475 | 25.965 | |
| trans-MUFA | 3.409 | 0.444 | 2.755 | 4.472 |  | 3.104 | 0.542 | 2.500 | 4.787 | |
| PUFA | 4.807 | 0.495 | 3.552 | 5.959 |  | 3.876 | 0.720 | 2.170 | 4.980 | |
| cis-PUFA | 3.363 | 0.400 | 2.065 | 3.952 |  | 2.694 | 0.659 | 1.203 | 3.701 | |
| trans-PUFA | 0.063 | 0.008 | 0.045 | 0.082 |  | 0.064 | 0.009 | 0.044 | 0.082 | |
| cis/trans PUFA | 1.380 | 0.178 | 1.067 | 1.925 |  | 1.118 | 0.141 | 0.863 | 1.376 | |
| ω-3 PUFA | 0.886 | 0.087 | 0.778 | 1.099 |  | 0.808 | 0.088 | 0.664 | 1.046 | |
| cis ω-3 PUFA | 0.654 | 0.070 | 0.572 | 0.846 |  | 0.601 | 0.084 | 0.489 | 0.838 | |
| ω-6 PUFA | 2.777 | 0.368 | 1.539 | 3.242 |  | 2.155 | 0.620 | 0.757 | 2.933 | |
| cis ω-6 PUFA | 2.710 | 0.366 | 1.473 | 3.167 |  | 2.093 | 0.615 | 0.714 | 2.863 | |
| ω-3 PUFA : ω-6 PUFA | 0.324 | 0.051 | 0.272 | 0.517 |  | 0.419 | 0.177 | 0.276 | 0.965 | |
|  | | | | | | | | | |  |

n = number of samples, SFA = saturated fatty acids, SD = standard deviation, Min = minimum value observed, Max = maximum value observed, nd = not detected, MUFA = monounsaturated fatty acids, PUFA = polyunsaturated fatty acids.

**Table S3.** Fatty acid profile of milk (n =16) for the extreme cows with contrasting potential for milk SFA.

|  | Extreme Low-SFA (n = 8) | | | |  | Extreme High-SFA (n =8) | | | | |
| --- | --- | --- | --- | --- | --- | --- | --- | --- | --- | --- |
|  | Mean | SD | Min | Max |  | Mean | SD | Min | Max | |
| **Individual fatty acids** |  |  |  |  |  |  |  |  |  | |
| C4:0 | 2.20 | 0.201 | 1.88 | 2.49 |  | 2.19 | 0.228 | 1.84 | 2.58 | |
| C5:0 | 0.01 | 0.005 | 0.01 | 0.02 |  | 0.02 | 0.003 | 0.02 | 0.03 | |
| C6:0 | 1.52 | 0.098 | 1.34 | 1.63 |  | 1.74 | 0.116 | 1.57 | 1.96 | |
| C7:0 | 0.01 | 0.004 | 0.01 | 0.02 |  | 0.02 | 0.006 | 0.02 | 0.03 | |
| C8:0 | 0.92 | 0.046 | 0.84 | 0.99 |  | 1.14 | 0.053 | 1.09 | 1.24 | |
| C9:0 | 0.02 | 0.003 | 0.01 | 0.02 |  | 0.03 | 0.012 | 0.02 | 0.05 | |
| C10:0 | 2.15 | 0.151 | 1.93 | 2.38 |  | 2.83 | 0.124 | 2.59 | 2.95 | |
| C10:1 c9 | 0.21 | 0.030 | 0.17 | 0.25 |  | 0.24 | 0.022 | 0.22 | 0.28 | |
| C11:0 | 0.03 | 0.006 | 0.03 | 0.04 |  | 0.06 | 0.020 | 0.04 | 0.10 | |
| C12:0 | 2.57 | 0.177 | 2.31 | 2.87 |  | 3.44 | 0.220 | 3.04 | 3.74 | |
| C13:0 iso | 0.02 | 0.004 | 0.01 | 0.02 |  | 0.02 | 0.002 | 0.02 | 0.02 | |
| C13:0 anteiso | 0.01 | 0.002 | 0.00 | 0.01 |  | 0.01 | 0.002 | 0.00 | 0.01 | |
| C12:1 c9 | 0.06 | 0.009 | 0.05 | 0.08 |  | 0.08 | 0.014 | 0.06 | 0.10 | |
| C13:0 | 0.07 | 0.009 | 0.05 | 0.08 |  | 0.09 | 0.026 | 0.06 | 0.14 | |
| C14:0 iso | 0.07 | 0.019 | 0.06 | 0.12 |  | 0.07 | 0.011 | 0.06 | 0.09 | |
| C14:0 | 9.8 | 0.50 | 9.1 | 10.7 |  | 11.4 | 0.60 | 10.5 | 12.3 | |
| C14:1 t9 | 0.18 | 0.011 | 0.16 | 0.19 |  | 0.17 | 0.016 | 0.14 | 0.18 | |
| C15:0 anteiso | 0.37 | 0.020 | 0.34 | 0.40 |  | 0.33 | 0.042 | 0.24 | 0.39 | |
| C14:1 c9 | 0.81 | 0.138 | 0.67 | 1.05 |  | 0.82 | 0.144 | 0.61 | 1.05 | |
| C15:0 | 0.79 | 0.047 | 0.72 | 0.86 |  | 0.88 | 0.162 | 0.72 | 1.21 | |
| C15:1 c9 | nd |  |  |  |  |  |  |  |  | |
| C16:0 iso | 0.22 | 0.035 | 0.16 | 0.28 |  | 0.21 | 0.032 | 0.16 | 0.24 | |
| C16:0 | 30.8 | 1.95 | 27.8 | 33.7 |  | 35.9 | 2.54 | 33.1 | 41.3 | |
| C16:1 c7-8 | 0.05 | 0.010 | 0.04 | 0.07 |  | 0.04 | 0.007 | 0.03 | 0.05 | |
| C16:1 t9 | 0.07 | 0.014 | 0.05 | 0.09 |  | 0.05 | 0.010 | 0.03 | 0.05 | |
| C17:0 iso | 0.23 | 0.033 | 0.18 | 0.29 |  | 0.16 | 0.011 | 0.14 | 0.17 | |
| C16:1 c9 | 1.41 | 0.234 | 1.15 | 1.88 |  | 1.30 | 0.210 | 0.99 | 1.58 | |
| C17:0 anteiso | 0.43 | 0.054 | 0.35 | 0.53 |  | 0.36 | 0.031 | 0.30 | 0.39 | |
| C16:1 c14 | 0.16 | 0.017 | 0.14 | 0.19 |  | 0.20 | 0.031 | 0.16 | 0.24 | |
| C17:0 | 0.46 | 0.045 | 0.41 | 0.54 |  | 0.41 | 0.026 | 0.36 | 0.44 | |
| C18:0 iso | 0.09 | 0.025 | 0.03 | 0.11 |  | 0.08 | 0.007 | 0.07 | 0.09 | |
| C17:1 c9 | 0.20 | 0.047 | 0.13 | 0.26 |  | 0.13 | 0.013 | 0.12 | 0.15 | |
| C18:0 | 9.43 | 1.101 | 7.91 | 10.69 |  | 9.88 | 0.764 | 8.92 | 11.02 | |
| C18:1 t4 | 0.02 | 0.008 | 0.01 | 0.03 |  | 0.02 | 0.006 | 0.02 | 0.04 | |
| C18:1 t5 | 0.02 | 0.016 | 0.00 | 0.05 |  | 0.02 | 0.005 | 0.01 | 0.03 | |
| C18:1 t6-8 | 0.39 | 0.080 | 0.28 | 0.51 |  | 0.33 | 0.070 | 0.23 | 0.48 | |
| C18:1 t9 | 0.34 | 0.053 | 0.25 | 0.42 |  | 0.25 | 0.057 | 0.20 | 0.38 | |
| C18:1 t10 | 0.53 | 0.122 | 0.38 | 0.72 |  | 0.29 | 0.089 | 0.17 | 0.44 | |
| C18:1 t11 | 1.30 | 0.229 | 0.97 | 1.66 |  | 1.03 | 0.177 | 0.78 | 1.36 | |
| C18:1 c6 + C18:1 t12 | nd |  |  |  |  |  |  |  |  | |
| C18:1 c9 | 24.1 | 1.16 | 23.2 | 26.4 |  | 18.0 | 1.19 | 16.7 | 19.9 | |
| C18:1 t15 | 0.59 | 0.297 | 0.36 | 1.20 |  | 0.69 | 0.413 | 0.19 | 1.27 | |
| C18:1 c11 | 0.93 | 0.070 | 0.83 | 1.03 |  | 0.67 | 0.069 | 0.53 | 0.75 | |
| C18:1 c12 | 0.40 | 0.085 | 0.26 | 0.49 |  | 0.31 | 0.068 | 0.17 | 0.38 | |
| C18:1 c13 | 0.09 | 0.028 | 0.04 | 0.14 |  | 0.06 | 0.025 | 0.04 | 0.12 | |
| C18:1 t16 + C18:1 c14 | 0.43 | 0.047 | 0.33 | 0.48 |  | 0.35 | 0.043 | 0.26 | 0.40 | |
| C19:0 + C18:1 c15 | 0.14 | 0.025 | 0.09 | 0.17 |  | 0.11 | 0.025 | 0.08 | 0.15 | |
| C18:2 t9t12 |  |  |  |  |  |  |  |  |  | |
| C18:2 t11t15 | 0.07 | 0.008 | 0.06 | 0.08 |  | 0.06 | 0.008 | 0.06 | 0.08 | |
| C18:2 c9t13 | 0.27 | 0.056 | 0.21 | 0.38 |  | 0.17 | 0.040 | 0.12 | 0.23 | |
| C18:2 c10t14 | 0.12 | 0.040 | 0.08 | 0.21 |  | 0.11 | 0.025 | 0.07 | 0.15 | |
| C18:2 c9t14 | 0.16 | 0.026 | 0.12 | 0.21 |  | 0.10 | 0.024 | 0.07 | 0.13 | |
| C18:2 c9t12 | 0.07 | 0.011 | 0.06 | 0.08 |  | 0.06 | 0.013 | 0.04 | 0.08 | |
| C18:1 c16 | 0.04 | 0.007 | 0.03 | 0.05 |  | 0.03 | 0.007 | 0.02 | 0.04 | |
| C18:2 t11c15 | 0.12 | 0.025 | 0.07 | 0.16 |  | 0.09 | 0.009 | 0.07 | 0.10 | |
| C18:2 c9c12 | 2.35 | 0.328 | 1.92 | 2.77 |  | 1.26 | 0.674 | 0.38 | 2.14 | |
| C19:1 c10 + C18:2c15 | 0.13 | 0.010 | 0.12 | 0.15 |  | 0.10 | 0.017 | 0.08 | 0.13 | |
| C20:0 | 0.12 | 0.009 | 0.11 | 0.13 |  | 0.12 | 0.007 | 0.11 | 0.13 | |
| C18:3 c6c9c12 | 0.03 | 0.005 | 0.03 | 0.04 |  | 0.02 | 0.004 | 0.02 | 0.03 | |
| C18:3 c9c12c15 | 0.51 | 0.044 | 0.45 | 0.57 |  | 0.39 | 0.043 | 0.28 | 0.41 | |
| C11 C20:1 | 0.08 | 0.005 | 0.07 | 0.08 |  | 0.06 | 0.004 | 0.05 | 0.06 | |
| C18:2 c9t11 | 0.71 | 0.085 | 0.53 | 0.82 |  | 0.47 | 0.056 | 0.40 | 0.58 | |
| C20:2 c11c14 | 0.03 | 0.014 | 0.02 | 0.06 |  | 0.03 | 0.009 | 0.02 | 0.05 | |
| C22:0 | 0.04 | 0.015 | 0.02 | 0.07 |  | 0.04 | 0.003 | 0.03 | 0.04 | |
| C20:3 c8c11c14 | 0.16 | 0.028 | 0.10 | 0.19 |  | 0.14 | 0.019 | 0.10 | 0.16 | |
| C22:1 c13 | nd |  |  |  |  |  |  |  |  | |
| C20:3 c11c14c17 | 0.01 | 0.005 | 0.01 | 0.02 |  | 0.01 | 0.002 | 0.01 | 0.01 | |
| C20:4 c5c8c11c14 | 0.14 | 0.019 | 0.11 | 0.16 |  | 0.12 | 0.018 | 0.08 | 0.14 | |
| C22:2 c13c16 | 0.03 | 0.015 | 0.02 | 0.07 |  | 0.02 | 0.003 | 0.02 | 0.03 | |
| C20:5 c5c8c11c14c17 | 0.04 | 0.019 | 0.03 | 0.09 |  | 0.03 | 0.004 | 0.03 | 0.04 | |
| C24:0 | 0.03 | 0.009 | 0.02 | 0.04 |  | 0.03 | 0.008 | 0.02 | 0.04 | |
| C22:3 c13c16c19 | 0.01 | 0.011 | 0.01 | 0.03 |  | 0.02 | 0.016 | 0.00 | 0.05 | |
| C22:4 c7c10c13c16 | 0.05 | 0.034 | 0.02 | 0.13 |  | 0.03 | 0.013 | 0.01 | 0.05 | |
| C22:5 c7c10c13c16c19 | 0.08 | 0.032 | 0.04 | 0.13 |  | 0.06 | 0.009 | 0.04 | 0.07 | |
| C22:6 c4c7c10c13c16c19 | 0.01 | 0.007 | 0.01 | 0.02 |  | 0.04 | 0.030 | 0.01 | 0.10 | |
| **Fatty acid Groups** |  |  |  |  |  |  |  |  |  | |
| SFA | 62.4 | 1.33 | 60.2 | 64.0 |  | 71.5 | 1.51 | 69.9 | 74.4 | |
| MUFA | 32.6 | 1.16 | 31.5 | 34.4 |  | 25.2 | 0.96 | 23.4 | 26.2 | |
| cis-MUFA | 28.9 | 1.15 | 28.0 | 31.0 |  | 22.1 | 1.12 | 20.5 | 23.5 | |
| trans-MUFA | 3.71 | 0.425 | 3.07 | 4.47 |  | 3.06 | 0.412 | 2.51 | 3.62 | |
| PUFA | 5.05 | 0.537 | 4.36 | 5.96 |  | 3.28 | 0.726 | 2.17 | 4.29 | |
| cis-PUFA | 3.47 | 0.379 | 3.02 | 3.95 |  | 2.16 | 0.701 | 1.20 | 3.04 | |
| trans-PUFA | 0.07 | 0.008 | 0.06 | 0.08 |  | 0.06 | 0.008 | 0.06 | 0.08 | |
| cis/trans PUFA | 1.51 | 0.211 | 1.19 | 1.93 |  | 1.06 | 0.146 | 0.86 | 1.29 | |
| ω-3 PUFA | 0.94 | 0.089 | 0.85 | 1.10 |  | 0.76 | 0.043 | 0.66 | 0.80 | |
| cis ω-3 PUFA | 0.68 | 0.066 | 0.61 | 0.79 |  | 0.55 | 0.029 | 0.49 | 0.59 | |
| ω-6 PUFA | 2.86 | 0.325 | 2.48 | 3.24 |  | 1.67 | 0.696 | 0.76 | 2.57 | |
| cis ω-6 PUFA | 2.78 | 0.318 | 2.41 | 3.17 |  | 1.61 | 0.691 | 0.71 | 2.49 | |
| ω-3 PUFA : ω-6 PUFA | 0.33 | 0.018 | 0.29 | 0.35 |  | 0.54 | 0.256 | 0.31 | 0.97 | |
|  | | | | | | | | | |  |

n = number of samples, SFA = saturated fatty acids, SD = standard deviation, Min = minimum value observed, Max = maximum value observed, nd = not detected, MUFA = monounsaturated fatty acids, PUFA = polyunsaturated fatty acids

**Table S4.** Metadata file including animal IDs and grouping information.

| **Animal ID** | **Metagenomics ID** | **Information** |
| --- | --- | --- |
| DC1 | 11289AM0001 | 2018 Holstein Medium Concentration Diet High Milk Saturated Fatty Acids |
| DC2 | 11289AM0002 | 2018 Holstein Medium Concentration Diet Medium Milk Saturated Fatty Acids |
| DC3 | 11289AM0003 | 2018 Holstein Medium Concentration Diet Medium Milk Saturated Fatty Acids |
| DC4 | 11289AM0004 | 2018 Holstein Medium Concentration Diet Medium Milk Saturated Fatty Acids |
| DC5 | 11289AM0005 | 2018 Holstein Medium Concentration Diet High Milk Saturated Fatty Acids |
| DC6 | 11289AM0006 | 2018 Holstein Medium Concentration Diet Low Milk Saturated Fatty Acids |
| DC7 | 11289AM0007 | 2018 Holstein Medium Concentration Diet High Milk Saturated Fatty Acids |
| DC8 | 11289AM0008 | 2018 Holstein Medium Concentration Diet Medium Milk Saturated Fatty Acids |
| DC9 | 11289AM0009 | 2018 Holstein Medium Concentration Diet Medium Milk Saturated Fatty Acids |
| DC10 | 11289AM0010 | 2018 Holstein Medium Concentration Diet Low Milk Saturated Fatty Acids |
| DC11 | 11289AM0011 | 2018 Holstein Medium Concentration Diet High Milk Saturated Fatty Acids |
| DC12 | 11289AM0012 | 2018 Holstein Medium Concentration Diet Medium Milk Saturated Fatty Acids |
| DC13 | 11289AM0013 | 2018 Holstein Medium Concentration Diet Medium Milk Saturated Fatty Acids |
| DC14 | 11289AM0014 | 2018 Holstein Medium Concentration Diet Medium Milk Saturated Fatty Acids |
| DC15 | 11289AM0015 | 2018 Holstein Medium Concentration Diet Low Milk Saturated Fatty Acids |
| DC16 | 11289AM0016 | 2018 Holstein Medium Concentration Diet Medium Milk Saturated Fatty Acids |
| DC17 | 11289AM0017 | 2018 Holstein Medium Concentration Diet Medium Milk Saturated Fatty Acids |
| DC18 | 11289AM0018 | 2018 Holstein Medium Concentration Diet High Milk Saturated Fatty Acids |
| DC19 | 11289AM0019 | 2018 Holstein Medium Concentration Diet High Milk Saturated Fatty Acids |
| DC20 | 11289AM0020 | 2018 Holstein Medium Concentration Diet Medium Milk Saturated Fatty Acids |
| DC21 | 11289AM0021 | 2018 Holstein Medium Concentration Diet Low Milk Saturated Fatty Acids |
| DC22 | 11289AM0022 | 2018 Holstein Medium Concentration Diet Medium Milk Saturated Fatty Acids |
| DC23 | 11289AM0023 | 2018 Holstein Medium Concentration Diet Medium Milk Saturated Fatty Acids |
| DC24 | 11289AM0024 | 2018 Holstein Medium Concentration Diet Medium Milk Saturated Fatty Acids |
| DC25 | 11289AM0025 | 2018 Holstein Medium Concentration Diet Medium Milk Saturated Fatty Acids |
| DC26 | 11289AM0026 | 2018 Holstein Medium Concentration Diet Medium Milk Saturated Fatty Acids |
| DC27 | 11289AM0027 | 2018 Holstein Medium Concentration Diet Medium Milk Saturated Fatty Acids |
| DC28 | 11289AM0028 | 2018 Holstein Medium Concentration Diet Medium Milk Saturated Fatty Acids |
| DC29 | 11289AM0029 | 2018 Holstein Medium Concentration Diet Medium Milk Saturated Fatty Acids |
| DC30 | 11289AM0030 | 2018 Holstein Medium Concentration Diet Medium Milk Saturated Fatty Acids |
| DC31 | 11289AM0031 | 2018 Holstein Medium Concentration Diet Medium Milk Saturated Fatty Acids |
| DC32 | 11289AM0032 | 2018 Holstein Medium Concentration Diet Medium Milk Saturated Fatty Acids |
| DC33 | 11289AM0033 | 2018 Holstein Medium Concentration Diet Medium Milk Saturated Fatty Acids |
| DC34 | 11289AM0034 | 2018 Holstein Medium Concentration Diet High Milk Saturated Fatty Acids |
| DC35 | 11289AM0035 | 2018 Holstein Medium Concentration Diet Low Milk Saturated Fatty Acids |
| DC36 | 11289AM0036 | 2018 Holstein Medium Concentration Diet Medium Milk Saturated Fatty Acids |
| DC37 | 11289AM0037 | 2018 Holstein Medium Concentration Diet Low Milk Saturated Fatty Acids |
| DC38 | 11289AM0038 | 2018 Holstein Medium Concentration Diet Medium Milk Saturated Fatty Acids |
| DC39 | 11289AM0039 | 2018 Holstein Medium Concentration Diet Low Milk Saturated Fatty Acids |
| DC40 | 11289AM0040 | 2018 Holstein Medium Concentration Diet Medium Milk Saturated Fatty Acids |
| DC41 | 11289AM0041 | 2018 Holstein Medium Concentration Diet Medium Milk Saturated Fatty Acids |
| DC42 | 11289AM0042 | 2018 Holstein Medium Concentration Diet High Milk Saturated Fatty Acids |
| DC43 | 11289AM0043 | 2018 Holstein Medium Concentration Diet Medium Milk Saturated Fatty Acids |
| DC44 | 11289AM0044 | 2018 Holstein Medium Concentration Diet Medium Milk Saturated Fatty Acids |
| DC45 | 11289AM0045 | 2018 Holstein Medium Concentration Diet Medium Milk Saturated Fatty Acids |
| DC46 | 11289AM0046 | 2018 Holstein Medium Concentration Diet Medium Milk Saturated Fatty Acids |
| DC47 | 11289AM0047 | 2018 Holstein Medium Concentration Diet Medium Milk Saturated Fatty Acids |
| DC48 | 11289AM0048 | 2018 Holstein Medium Concentration Diet Low Milk Saturated Fatty Acids |

**Table S5.** Fatty acid profile of rumen fluids (n =48) for the cows with contrasting potential for milk SFA.

|  | Extreme Low-SFA (n = 8) | | | |  | Extreme High-SFA (n =8) | | | | |
| --- | --- | --- | --- | --- | --- | --- | --- | --- | --- | --- |
|  | Mean | SD | Min | Max |  | Mean | SD | Min | Max | |
| **Individual fatty acids** |  |  |  |  |  |  |  |  |  | |
| C4:0 | 0.94 | 0.681 | 0.16 | 2.23 |  | 2.24 | 1.908 | 0.4 | 5.69 | |
| C5:0 | 0.29 | 0.225 | 0.04 | 0.71 |  | 0.71 | 0.68 | 0.13 | 1.9 | |
| C6:0 | 0.35 | 0.345 | 0.02 | 0.96 |  | 0.7 | 0.667 | 0.12 | 1.64 | |
| C7:0 | nd |  |  |  |  | nd |  |  |  | |
| C8:0 | nd |  |  |  |  | nd |  |  |  | |
| C9:0 | nd |  |  |  |  | nd |  |  |  | |
| C10:0 | nd |  |  |  |  | nd |  |  |  | |
| C10:1 c9 | nd |  |  |  |  | nd |  |  |  | |
| C11:0 | 0.04 | 0.012 | 0.03 | 0.06 |  | 0.07 | 0.013 | 0.04 | 0.08 | |
| C12:0 | 0.15 | 0.056 | 0.09 | 0.28 |  | 0.14 | 0.036 | 0.1 | 0.21 | |
| C13:0 iso | 0.05 | 0.028 | 0 | 0.08 |  | 0.05 | 0.03 | 0.01 | 0.09 | |
| C13:0 anteiso | 0.03 | 0.03 | 0.01 | 0.08 |  | 0.06 | 0.043 | 0.01 | 0.14 | |
| C12:1 c9 | 0.22 | 0.027 | 0.18 | 0.26 |  | 0.25 | 0.092 | 0.14 | 0.38 | |
| C13:0 | 0.05 | 0.013 | 0.03 | 0.07 |  | 0.04 | 0.015 | 0.02 | 0.07 | |
| C14:0 iso | 0.17 | 0.053 | 0.11 | 0.29 |  | 0.16 | 0.049 | 0.08 | 0.22 | |
| C14:0 | 0.52 | 0.108 | 0.43 | 0.69 |  | 0.61 | 0.189 | 0.34 | 0.87 | |
| C14:1 t9 | 0.46 | 0.117 | 0.33 | 0.72 |  | 0.43 | 0.119 | 0.23 | 0.54 | |
| C15:0 anteiso | 1.13 | 0.23 | 0.79 | 1.51 |  | 1.14 | 0.448 | 0.51 | 1.95 | |
| C14:1 c9 | nd |  |  |  |  | nd |  |  |  | |
| C15:0 | 0.9 | 0.131 | 0.75 | 1.19 |  | 0.88 | 0.159 | 0.62 | 1.07 | |
| C15:1 c9 | 1.21 | 0.237 | 0.9 | 1.53 |  | 1.41 | 0.511 | 0.72 | 2.03 | |
| C16:0 iso | 0.41 | 0.103 | 0.29 | 0.6 |  | 0.41 | 0.131 | 0.25 | 0.62 | |
| C16:0 | 23.7 | 0.8 | 22.5 | 24.6 |  | 23.5 | 1.92 | 19.6 | 26 | |
| C16:1 c7-8 | nd |  |  |  |  | nd |  |  |  | |
| C16:1 t9 | nd |  |  |  |  | nd |  |  |  | |
| C17:0 iso | 0.18 | 0.08 | 0.1 | 0.34 |  | 0.18 | 0.051 | 0.11 | 0.26 | |
| C16:1 c9 | nd |  |  |  |  | nd |  |  |  | |
| C17:0 anteiso | 0.35 | 0.172 | 0.05 | 0.63 |  | 0.4 | 0.149 | 0.17 | 0.58 | |
| C16:1 c14 | 0.56 | 0.118 | 0.42 | 0.77 |  | 0.57 | 0.125 | 0.31 | 0.74 | |
| C17:0 | 0.42 | 0.039 | 0.39 | 0.5 |  | 0.41 | 0.042 | 0.36 | 0.48 | |
| C18:0 iso | 0.14 | 0.047 | 0.06 | 0.2 |  | 0.08 | 0.056 | 0 | 0.16 | |
| C17:1 c9 | 0.07 | 0.033 | 0.03 | 0.13 |  | 0.08 | 0.048 | 0.03 | 0.17 | |
| C18:0 | 45.1 | 1.917 | 43 | 48.6 |  | 44.2 | 5.593 | 37.7 | 54.1 | |
| C18:1 t4 | 0.08 | 0.028 | 0.03 | 0.13 |  | 0.07 | 0.025 | 0.02 | 0.1 | |
| C18:1 t5 | 0.07 | 0.035 | 0.02 | 0.12 |  | 0.07 | 0.032 | 0.01 | 0.11 | |
| C18:1 t6-8 | 0.7 | 0.137 | 0.48 | 0.89 |  | 0.67 | 0.148 | 0.51 | 0.96 | |
| C18:1 t9 | 0.47 | 0.079 | 0.35 | 0.61 |  | 0.45 | 0.086 | 0.34 | 0.64 | |
| C18:1 t10 | 1.06 | 0.33 | 0.74 | 1.79 |  | 0.88 | 0.239 | 0.5 | 1.22 | |
| C18:1 t11 | 3.31 | 0.72 | 2.34 | 4.37 |  | 3.04 | 0.843 | 2.04 | 4.88 | |
| C18:1 c6 + C18:1 t12 | 0.92 | 0.143 | 0.69 | 1.09 |  | 0.88 | 0.171 | 0.65 | 1.2 | |
| C18:1 c9 | 7.08 | 0.46 | 6.49 | 7.81 |  | 6.9 | 0.832 | 6.06 | 8.27 | |
| C18:1 t15 | 0.81 | 0.106 | 0.64 | 0.95 |  | 0.71 | 0.092 | 0.6 | 0.84 | |
| C18:1 c11 | 0.67 | 0.122 | 0.56 | 0.95 |  | 0.63 | 0.102 | 0.45 | 0.75 | |
| C18:1 c12 | 0.55 | 0.168 | 0.33 | 0.72 |  | 0.62 | 0.294 | 0.23 | 1.12 | |
| C18:1 c13 | nd |  |  |  |  | nd |  |  |  | |
| C18:1 t16 + C18:1 c14 | 0.78 | 0.084 | 0.64 | 0.88 |  | 0.67 | 0.085 | 0.55 | 0.81 | |
| C19:0 + C18:1 c15 | 0.13 | 0.039 | 0.05 | 0.17 |  | 0.08 | 0.039 | 0.02 | 0.13 | |
| C18:2 t9t12 | 0.28 | 0.057 | 0.23 | 0.42 |  | 0.23 | 0.095 | 0.11 | 0.33 | |
| C18:2 t11t15 | 0.24 | 0.051 | 0.15 | 0.31 |  | 0.25 | 0.069 | 0.14 | 0.35 | |
| C18:2 c9t13 | nd |  |  |  |  | nd |  |  |  | |
| C18:2 c10t14 | nd |  |  |  |  | nd |  |  |  | |
| C18:2 c9t14 | nd |  |  |  |  | nd |  |  |  | |
| C18:2 c9t12 | nd |  |  |  |  | nd |  |  |  | |
| C18:1 c16 | nd |  |  |  |  | nd |  |  |  | |
| C18:2 t11c15 | nd |  |  |  |  | nd |  |  |  | |
| C18:2 c9c12 | 3.1 | 0.778 | 2.12 | 4.74 |  | 2.92 | 0.821 | 1.83 | 3.75 | |
| C19:1 c10 + C18:2c15 | nd |  |  |  |  | nd |  |  |  | |
| C20:0 | 0.52 | 0.021 | 0.49 | 0.56 |  | 0.54 | 0.041 | 0.49 | 0.61 | |
| C18:3 c6c9c12 | nd |  |  |  |  | nd |  |  |  | |
| C18:3 c9c12c15 | 0.66 | 0.124 | 0.49 | 0.88 |  | 0.55 | 0.122 | 0.32 | 0.68 | |
| C11 C20:1 | 0.07 | 0.033 | 0.01 | 0.1 |  | 0.07 | 0.064 | 0.01 | 0.18 | |
| C18:2 c9t11 | 0.27 | 0.162 | 0.12 | 0.56 |  | 0.42 | 0.314 | 0.14 | 1.09 | |
| C20:2 c11c14 | nd |  |  |  |  | nd |  |  |  | |
| C22:0 | 0.04 | 0.035 | 0 | 0.1 |  | 0.04 | 0.046 | 0 | 0.13 | |
| C20:3 c8c11c14 | 0.29 | 0.115 | 0.12 | 0.42 |  | 0.24 | 0.024 | 0.21 | 0.27 | |
| C22:1 c13 | 0.01 | 0.024 | 0 | 0.07 |  | 0.01 | 0.012 | 0 | 0.04 | |
| C20:3 c11c14c17 | nd |  |  |  |  | nd |  |  |  | |
| C20:4 c5c8c11c14 | 0.03 | 0.029 | 0 | 0.1 |  | 0.03 | 0.021 | 0 | 0.06 | |
| C22:2 c13c16 | 0.08 | 0.192 | 0 | 0.55 |  | 0.01 | 0.006 | 0 | 0.02 | |
| C20:5 c5c8c11c14c17 | 0.04 | 0.049 | 0 | 0.13 |  | 0.02 | 0.011 | 0 | 0.04 | |
| C24:0 | 0.27 | 0.066 | 0.11 | 0.31 |  | 0.27 | 0.028 | 0.22 | 0.3 | |
| C22:3 c13c16c19 | 0.05 | 0.021 | 0 | 0.08 |  | 0.04 | 0.035 | 0 | 0.12 | |
| C22:4 c7c10c13c16 | nd |  |  |  |  | nd |  |  |  | |
| C22:5 c7c10c13c16c19 | nd |  |  |  |  | nd |  |  |  | |
| C22:6 c4c7c10c13c16c19 | nd |  |  |  |  | nd |  |  |  | |
| **Fatty acid Groups** |  |  |  |  |  |  |  |  |  | |
| SFA | 75.75 | 1.61 | 69.45 | 64.0 |  | 76.83 | 1.62 | 61.28 | 97.17 | |
| MUFA | 18.01 | 0.64 | 14.33 | 34.4 |  | 17.07 | 0.65 | 12.7 | 23.04 | |
| cis-MUFA | 9.35 | 0.37 | 8.07 | 31.0 |  | 9.2 | 0.47 | 7.25 | 11.74 | |
| trans-MUFA | 8.66 | 0.99 | 6.26 | 4.47 |  | 7.87 | 1.06 | 5.45 | 11.3 | |
| PUFA | 5.04 | 0.54 | 3.23 | 5.96 |  | 4.71 | 1.04 | 2.75 | 6.71 | |
| cis-PUFA | 4.25 | 0.46 | 2.73 | 3.95 |  | 3.81 | 1.24 | 2.36 | 4.94 | |
| trans-PUFA | 0.79 | 0.09 | 0.5 | 0.08 |  | 0.9 | 0.12 | 0.39 | 1.77 | |
| cis ω-3 PUFA | 0.66 | 0.06 | 0.49 | 0.88 |  | 0.55 | 0.03 | 0.32 | 0.68 | |
|  | | | | | | | | | |  |

n = number of samples, SFA = saturated fatty acids, SD = standard deviation, Min = minimum value observed, Max = maximum value observed, nd = not detected, MUFA = monounsaturated fatty acids, PUFA = polyunsaturated fatty acids

**Table S6.** Partial Least Square results between selected milk fatty acids and microbial genera.

| **Type of fatty acid: SFA** | **Percentage of variability explained: 78.3%** | |
| --- | --- | --- |
| VIP* | Coefficient | MAG/Genus |
| 1.20344 | 0.17288 | *Denitrobacterium* |
| 1.16693 | 0.22532 | *Rhodotorula* |
| 1.16608 | 0.23288 | *Mycobacterium* |
| 1.04417 | 0.06465 | *Hafnia* |
| 1.03121 | 0.05614 | *Kozakia* |
| 0.98442 | 0.19698 | *Dehalococcoides* |
| 0.97214 | 0.23054 | *Nannochloropsis* |
| 0.94524 | 0.03356 | *Weissella* |
| 0.94219 | -0.00312 | *Acetobacter* |
| 0.94151 | 0.03704 | *Leuconostoc* |
| 0.92702 | 0.02063 | *Lactobacillus* |
| 0.83456 | -0.19556 | *Providencia* |
| **Type of fatty acid: C12:0** | **Percentage of variability explained: 66.7%** | |
| VIP* | Coefficient | MAG/Genus |
| 1.48165 | 0.3971 | *Jeotgalibacillus* |
| 1.34461 | 0.2813 | *Denitrobacterium* |
| 0.99911 | 0.05999 | *Lactobacillus* |
| 0.9914 | 0.06482 | *Leuconostoc* |
| 0.98487 | 0.0666 | *Weissella* |
| 0.96607 | -0.02282 | *Pediococcus* |
| 0.95156 | -0.03577 | *Hafnia* |
| 0.92393 | -0.00248 | *Komagataeibacter* |
| 0.88394 | 0.09067 | *Rahnella* |
| 0.88059 | 0.04242 | *Kozakia* |
| 0.85571 | 0.00845 | *Acetobacter* |
| **Type of fatty acid: C14:0** | **Percentage of variability explained: 73.8%** | |
| VIP* | Coefficient | MAG/Genus |
| 1.38533 | -0.25198 | *Bacteroides* |
| 1.18788 | 0.23149 | *Jeotgalibacillus* |
| 1.13598 | 0.17849 | *Denitrobacterium* |
| 1.08881 | 0.00337 | *Pediococcus* |
| 1.02264 | 0.06996 | *Rahnella* |
| 0.9824 | 0.22477 | *Virgibacillus* |
| 0.96637 | 0.07217 | *Lactobacillus* |
| 0.90975 | 0.07818 | *Leuconostoc* |
| 0.90374 | 0.03367 | *Oribacterium* |
| 0.89683 | 0.08579 | *Weissella* |
| 0.88378 | 0.11232 | *Carboxydothermus* |
| 0.8461 | 0.10134 | *Mageeibacillus* |
| 0.81145 | 0.13236 | *Psychrobacter* |
| 0.80309 | 0.16724 | *Enterococcus* |
| **Type of fatty acid: C16:0** | **Percentage of variability explained: 64.3%** | |
| VIP* | Coefficient | Genus |
| 1.23607 | 0.40728 | *Kozakia* |
| 1.0322 | -0.03076 | *Mycobacterium* |
| 1.02646 | 0.00838 | *Rhodotorula* |
| 1.00727 | 0.28299 | *Acetitomaculum* |
| 1.00553 | -0.03805 | *Anaerolinea* |
| 0.99408 | -0.02069 | *Brenneria* |
| 0.99376 | 0.10428 | *Desulfobacula* |
| 0.94898 | 0.00712 | *Punctularia* |
| 0.92677 | 0.02548 | *Cyphellophora* |
| 0.8981 | 0.23822 | *Dehalococcoides* |
| 0.88632 | 0.16769 | *Hafnia* |
| **Type of fatty acid: ALNA** | **Percentage of variability explained: 64.3%** | |
| VIP* | Coefficient | Genus |
| 1.33633 | -0.08433 | *Dehalococcoides* |
| 1.30352 | -0.0901 | *Rhodotorula* |
| 1.26692 | -0.08766 | *Mycobacterium* |
| 1.25927 | -0.0842 | *Desulfobacula* |
| 1.24969 | -0.08822 | *Cyphellophora* |
| 1.20636 | -0.08055 | *Singulisphaera* |
| 1.20192 | -0.08327 | *Denitrobacterium* |
| 1.19601 | -0.07972 | *Anaerolinea* |
| 1.17391 | -0.07823 | *Rhodopirellula* |
| 1.15303 | -0.07399 | *Brenneria* |
| 0.94164 | 0.023547 | *Borreliella* |
| 0.90901 | 0.034701 | *Deferribacter* |
| 0.90698 | 0.033415 | *Halobacteroides* |
| 0.90297 | 0.023641 | *Terrisporobacter* |
| 0.89802 | 0.029672 | *Arsenophonus* |
| 0.89408 | 0.018539 | *Buchnera* |
| 0.89292 | 0.025182 | *Candidatus Profftella* |
| 0.88323 | 0.018151 | *Candidatus Carsonella* |
| 0.87391 | 0.019521 | *Komagataella* |
| 0.87358 | 0.023697 | *Halanaerobium* |
| 0.87198 | 0.019156 | *Campylobacter* |
| 0.87052 | 0.018836 | *Naegleria* |
| 0.86708 | 0.02535 | *Thermodesulfobacterium* |
| 0.86595 | 0.024792 | *Ehrlichia* |
| 0.86484 | 0.016157 | *Cavenderia* |
| 0.86284 | 0.016602 | *Ichthyophthirius* |
| 0.85926 | 0.019609 | *Fomitiporia* |
| 0.8558 | -0.05383 | *Olsenella* |
| 0.85216 | 0.015219 | *Borrelia* |
| 0.82235 | -0.05782 | *Nannochloropsis* |
| 0.81343 | 0.014534 | *Candidatus Sulcia* |
| **Type of fatty acid: MUFA** | **Percentage of variability explained: 79.4%** | |
| VIP* | Coefficient | Genus |
| 1.23521 | -0.1802 | *Denitrobacterium* |
| 1.10176 | -0.2675 | *Nannochloropsis* |
| 1.09353 | 0.26739 | *Belliella* |
| 1.05748 | 0.00228 | *Kozakia* |
| 1.05453 | -0.14669 | *Rhodotorula* |
| 1.05326 | -0.02083 | *Hafnia* |
| 1.03418 | -0.1508 | *Mycobacterium* |
| 0.98377 | -0.11729 | *Punctularia* |
| 0.96995 | -0.15339 | *Cutaneotrichosporon* |
| 0.9689 | 0.04288 | *Acetobacter* |
| 0.93516 | -0.18309 | *Dehalococcoides* |
| 0.91274 | -0.01685 | *Weissella* |
| 0.90649 | -0.03469 | *Leuconostoc* |
| 0.89359 | -0.01805 | *Lactobacillus* |
| **Type of fatty acid: PUFA** | **Percentage of variability explained: 72.1%** | |
| VIP* | Coefficient | Genus |
| 1.51471 | 0.2807 | *Sutterella* |
| 1.30642 | -0.14031 | *Dehalococcoides* |
| 1.2569 | 0.04327 | *Hafnia* |
| 1.09481 | -0.13852 | *Kozakia* |
| 1.09039 | 0.02715 | *Atopobium* |
| 1.02862 | -0.05595 | *Denitrobacterium* |
| 1.02432 | -0.00967 | *Brenneria* |
| 1.01892 | -0.05776 | *Chryseobacterium* |
| 1.00235 | -0.02706 | *Singulisphaera* |
| 0.99981 | -0.12455 | *Weissella* |
| 0.98809 | -0.10538 | *Desulfobacula* |
| 0.98146 | -0.09165 | *Rhodotorula* |
| 0.95792 | -0.14022 | *Acetobacter* |
| 0.94504 | -0.05216 | *Cryptobacterium* |
| 0.87988 | 0.05587 | *Komagataella* |
| 0.8788 | -0.08575 | *Leuconostoc* |
| 0.83897 | 0.02963 | *Nosema* |
| **Type of fatty acid: n-3** | **Percentage of variability explained: 86.2%** | |
| VIP* | Coefficient | Genus |
| 1.44103 | -0.13334 | *Dehalococcoides* |
| 1.30631 | -0.11843 | *Denitrobacterium* |
| 1.20858 | 0.06975 | *Halobacteroides* |
| 1.13221 | 0.07059 | *Deferribacter* |
| 1.09557 | -0.15604 | *Nannochloropsis* |
| 1.04218 | -0.08574 | *Rhodotorula* |
| 1.03704 | -0.10208 | *Cryptobacterium* |
| 1.02647 | 0.08603 | *Methanotorris* |
| 1.02376 | -0.01949 | *Selenomonas* |
| 1.01514 | -0.13361 | *Croceibacter* |
| 1.00045 | -0.08378 | *Mycobacterium* |
| 0.98232 | -0.0858 | *Bibersteinia* |
| 0.968 | 0.03574 | *Wickerhamomyces* |
| 0.94945 | -0.10218 | *Cyphellophora* |
| 0.89458 | 0.06858 | *Meyerozyma* |
| 0.89387 | 0.12329 | *Fibrobacter* |
| 0.88234 | 0.13257 | *Providencia* |
| 0.85044 | -0.02496 | *Caldithrix* |
| 0.84898 | -0.03074 | *Petrimonas* |
| 0.81268 | 0.12224 | *Proteus* |

***** Variable importance for projection (VIP)

**Table S7.** ANOVA results for genes significantly different between Low- compared to High-SFA groups of 24 vs 24 or 8 vs 8 extreme animals.

| **Comparison between Low- compared to High-SFA groups for the 48 animals** | | | | |
| --- | --- | --- | --- | --- |
| **KEGG ID** | **FUNCTION** | **Mean LOW** | **Mean HIGH** | ***P*-value** |
| K00013 | histidinol dehydrogenase [EC:1.1.1.23] | 0.0647 | 0.0578 | 0.023 |
| K00027 | malate dehydrogenase (oxaloacetate-decarboxylating) [EC:1.1.1.38] | 0.0038 | 0.0040 | 0.039 |
| K00040 | fructuronate reductase [EC:1.1.1.57] | 0.1357 | 0.1206 | 0.007 |
| K00057 | glycerol-3-phosphate dehydrogenase (NAD(P)+) [EC:1.1.1.94] | 0.0681 | 0.0614 | 0.039 |
| K00088 | IMP dehydrogenase [EC:1.1.1.205] | 0.1439 | 0.1475 | 0.045 |
| K00123 | formate dehydrogenase, alpha subunit [EC:1.2.1.2] | 0.0613 | 0.0868 | 0.003 |
| K00125 | formate dehydrogenase, beta subunit [EC:1.2.1.2] | 0.0198 | 0.0300 | 0.014 |
| K00150 | glyceraldehyde-3-phosphate dehydrogenase (NAD(P)) [EC:1.2.1.59] | 0.0121 | 0.0202 | 0.013 |
| K00169 | pyruvate ferredoxin oxidoreductase, alpha subunit [EC:1.2.7.1] | 0.0134 | 0.0210 | 0.022 |
| K00170 | pyruvate ferredoxin oxidoreductase, beta subunit [EC:1.2.7.1] | 0.0100 | 0.0163 | 0.007 |
| K00171 | pyruvate ferredoxin oxidoreductase, delta subunit [EC:1.2.7.1] | 0.0025 | 0.0041 | 0.005 |
| K00172 | pyruvate ferredoxin oxidoreductase, gamma subunit [EC:1.2.7.1] | 0.0044 | 0.0070 | 0.034 |
| K00186 | 2-oxoisovalerate ferredoxin oxidoreductase, alpha subunit [EC:1.2.7.7] | 0.0026 | 0.0043 | 0.019 |
| K00187 | 2-oxoisovalerate ferredoxin oxidoreductase, beta subunit [EC:1.2.7.7] | 0.0058 | 0.0096 | 0.021 |
| K00200 | formylmethanofuran dehydrogenase subunit A [EC:1.2.99.5] | 0.0304 | 0.0467 | 0.006 |
| K00201 | formylmethanofuran dehydrogenase subunit B [EC:1.2.99.5] | 0.0459 | 0.0671 | 0.002 |
| K00202 | formylmethanofuran dehydrogenase subunit C [EC:1.2.99.5] | 0.0174 | 0.0255 | 0.011 |
| K00203 | formylmethanofuran dehydrogenase subunit D [EC:1.2.99.5] | 0.0077 | 0.0123 | 0.003 |
| K00204 | formylmethanofuran dehydrogenase subunit H [EC:1.2.99.5] | 0.0017 | 0.0034 | 0.029 |
| K00283 | glycine dehydrogenase subunit 2 [EC:1.4.4.2] | 0.0533 | 0.0454 | 0.017 |
| K00319 | methylenetetrahydromethanopterin dehydrogenase [EC:1.5.99.9] | 0.0285 | 0.0413 | 0.009 |
| K00320 | coenzyme F420-dependent N5,N10-methenyltetrahydromethanopterin reductase [EC:1.5.99.11] | 0.0318 | 0.0459 | 0.006 |
| K00382 | dihydrolipoamide dehydrogenase [EC:1.8.1.4] | 0.0186 | 0.0174 | 0.041 |
| K00399 | methyl-coenzyme M reductase alpha subunit [EC:2.8.4.1] | 0.0700 | 0.0979 | 0.007 |
| K00400 | methyl coenzyme M reductase system, component A2 | 0.0094 | 0.0139 | 0.021 |
| K00401 | methyl-coenzyme M reductase beta subunit [EC:2.8.4.1] | 0.0370 | 0.0522 | 0.009 |
| K00402 | methyl-coenzyme M reductase gamma subunit [EC:2.8.4.1] | 0.0240 | 0.0332 | 0.007 |
| K00440 | coenzyme F420 hydrogenase alpha subunit [EC:1.12.98.1] | 0.0110 | 0.0167 | 0.034 |
| K00443 | coenzyme F420 hydrogenase gamma subunit [EC:1.12.98.1] | 0.0064 | 0.0107 | 0.026 |
| K00560 | thymidylate synthase [EC:2.1.1.45] | 0.0605 | 0.0578 | 0.037 |
| K00577 | tetrahydromethanopterin S-methyltransferase subunit A [EC:2.1.1.86] | 0.0112 | 0.0185 | 0.012 |
| K00580 | tetrahydromethanopterin S-methyltransferase subunit D [EC:2.1.1.86] | 0.0114 | 0.0185 | 0.005 |
| K00581 | tetrahydromethanopterin S-methyltransferase subunit E [EC:2.1.1.86] | 0.0277 | 0.0409 | 0.007 |
| K00584 | tetrahydromethanopterin S-methyltransferase subunit H [EC:2.1.1.86] | 0.0307 | 0.0463 | 0.005 |
| K00602 | phosphoribosylaminoimidazolecarboxamide formyltransferase / IMP cyclohydrolase [EC:2.1.2.3 3.5.4.10] | 0.1356 | 0.1264 | 0.036 |
| K00606 | 3-methyl-2-oxobutanoate hydroxymethyltransferase [EC:2.1.2.11] | 0.0350 | 0.0293 | 0.016 |
| K00626 | acetyl-CoA C-acetyltransferase [EC:2.3.1.9] | 0.0226 | 0.0337 | 0.030 |
| K00634 | phosphate butyryltransferase [EC:2.3.1.19] | 0.0158 | 0.0155 | 0.041 |
| K00651 | homoserine O-succinyltransferase [EC:2.3.1.46] | 0.0195 | 0.0156 | 0.021 |
| K00656 | formate C-acetyltransferase [EC:2.3.1.54] | 0.2109 | 0.1788 | 0.013 |
| K00672 | formylmethanofuran--tetrahydromethanopterin N-formyltransferase [EC:2.3.1.101] | 0.0094 | 0.0182 | 0.013 |
| K00677 | UDP-N-acetylglucosamine acyltransferase [EC:2.3.1.129] | 0.0597 | 0.0543 | 0.028 |
| K00700 | 1,4-alpha-glucan branching enzyme [EC:2.4.1.18] | 0.1542 | 0.1489 | 0.035 |
| K00705 | 4-alpha-glucanotransferase [EC:2.4.1.25] | 0.1091 | 0.1009 | 0.038 |
| K00757 | uridine phosphorylase [EC:2.4.2.3] | 0.0340 | 0.0277 | 0.021 |
| K00761 | uracil phosphoribosyltransferase [EC:2.4.2.9] | 0.0116 | 0.0104 | 0.013 |
| K00765 | ATP phosphoribosyltransferase [EC:2.4.2.17] | 0.0673 | 0.0605 | 0.038 |
| K00768 | nicotinate-nucleotide--dimethylbenzimidazole phosphoribosyltransferase [EC:2.4.2.21] | 0.0109 | 0.0092 | 0.026 |
| K00818 | acetylornithine aminotransferase [EC:2.6.1.11] | 0.0297 | 0.0242 | 0.023 |
| K00847 | fructokinase [EC:2.7.1.4] | 0.0292 | 0.0270 | 0.027 |
| K00857 | thymidine kinase [EC:2.7.1.21] | 0.0154 | 0.0127 | 0.035 |
| K00864 | glycerol kinase [EC:2.7.1.30] | 0.0018 | 0.0025 | 0.011 |
| K00876 | uridine kinase [EC:2.7.1.48] | 0.0605 | 0.0529 | 0.033 |
| K00925 | acetate kinase [EC:2.7.2.1] | 0.1933 | 0.1817 | 0.032 |
| K00928 | aspartate kinase [EC:2.7.2.4] | 0.1076 | 0.0969 | 0.013 |
| K00940 | nucleoside-diphosphate kinase [EC:2.7.4.6] | 0.0169 | 0.0221 | 0.016 |
| K00951 | GTP pyrophosphokinase [EC:2.7.6.5] | 0.1520 | 0.1393 | 0.033 |
| K00990 | [protein-PII] uridylyltransferase [EC:2.7.7.59] | 0.0015 | 0.0007 | 0.039 |
| K01007 | pyruvate, water dikinase [EC:2.7.9.2] | 0.0294 | 0.0410 | 0.022 |
| K01079 | phosphoserine phosphatase [EC:3.1.3.3] | 0.0731 | 0.0844 | 0.036 |
| K01089 | imidazoleglycerol-phosphate dehydratase / histidinol-phosphatase [EC:4.2.1.19 3.1.3.15] | 0.0271 | 0.0215 | 0.016 |
| K01090 | protein phosphatase [EC:3.1.3.16] | 0.0018 | 0.0010 | 0.041 |
| K01270 | aminoacylhistidine dipeptidase [EC:3.4.13.3] | 0.0856 | 0.0709 | 0.015 |
| K01499 | methenyltetrahydromethanopterin cyclohydrolase [EC:3.5.4.27] | 0.0194 | 0.0314 | 0.008 |
| K01516 | nucleoside-triphosphatase [EC:3.6.1.15] | 0.0299 | 0.0376 | 0.027 |
| K01551 | arsenite-transporting ATPase [EC:3.6.3.16] | 0.0024 | 0.0048 | 0.047 |
| K01591 | orotidine-5prime-phosphate decarboxylase [EC:4.1.1.23] | 0.0416 | 0.0354 | 0.025 |
| K01619 | deoxyribose-phosphate aldolase [EC:4.1.2.4] | 0.0420 | 0.0342 | 0.036 |
| K01622 | fructose 1,6-bisphosphate aldolase/phosphatase [EC:4.1.2.13 3.1.3.11] | 0.0301 | 0.0447 | 0.008 |
| K01623 | fructose-bisphosphate aldolase, class I [EC:4.1.2.13] | 0.0027 | 0.0042 | 0.001 |
| K01673 | carbonic anhydrase [EC:4.2.1.1] | 0.0039 | 0.0058 | 0.012 |
| K01709 | CDP-glucose 4,6-dehydratase [EC:4.2.1.45] | 0.0061 | 0.0072 | 0.001 |
| K01784 | UDP-glucose 4-epimerase [EC:5.1.3.2] | 0.0897 | 0.0850 | 0.032 |
| K01872 | alanyl-tRNA synthetase [EC:6.1.1.7] | 0.3184 | 0.3093 | 0.005 |
| K01875 | seryl-tRNA synthetase [EC:6.1.1.11] | 0.1840 | 0.1769 | 0.001 |
| K01885 | glutamyl-tRNA synthetase [EC:6.1.1.17] | 0.1601 | 0.1484 | 0.008 |
| K01897 | long-chain acyl-CoA synthetase [EC:6.2.1.3] | 0.1086 | 0.0897 | 0.024 |
| K01912 | phenylacetate-CoA ligase [EC:6.2.1.30] | 0.1730 | 0.1609 | 0.023 |
| K01915 | glutamine synthetase [EC:6.3.1.2] | 0.4029 | 0.3921 | 0.036 |
| K01918 | pantoate--beta-alanine ligase [EC:6.3.2.1] | 0.0323 | 0.0285 | 0.049 |
| K01929 | UDP-N-acetylmuramoylalanyl-D-glutamyl-2,6-diaminopimelate--D-alanyl-D-alanine ligase [EC:6.3.2.10] | 0.0500 | 0.0456 | 0.038 |
| K01939 | adenylosuccinate synthase [EC:6.3.4.4] | 0.1347 | 0.1260 | 0.044 |
| K01959 | pyruvate carboxylase subunit A [EC:6.4.1.1] | 0.0138 | 0.0204 | 0.009 |
| K01983 | SRP RNA | 0.0056 | 0.0086 | 0.028 |
| K01990 | ABC-2 type transport system ATP-binding protein | 0.0626 | 0.0563 | 0.044 |
| K01991 | polysaccharide export outer membrane protein | 0.0084 | 0.0076 | 0.011 |
| K02005 | HlyD family secretion protein | 0.1192 | 0.1077 | 0.039 |
| K02007 | cobalt/nickel transport system permease protein | 0.0171 | 0.0265 | 0.017 |
| K02009 | cobalt transport protein | 0.0032 | 0.0045 | 0.014 |
| K02018 | molybdate transport system permease protein | 0.0051 | 0.0097 | 0.012 |
| K02020 | molybdate transport system substrate-binding protein | 0.0054 | 0.0087 | 0.015 |
| K02108 | F-type H+-transporting ATPase subunit a [EC:3.6.3.14] | 0.0425 | 0.0368 | 0.037 |
| K02109 | F-type H+-transporting ATPase subunit b [EC:3.6.3.14] | 0.0196 | 0.0159 | 0.031 |
| K02117 | V-type H+-transporting ATPase subunit A [EC:3.6.3.14] | 0.0980 | 0.1245 | 0.009 |
| K02118 | V-type H+-transporting ATPase subunit B [EC:3.6.3.14] | 0.0815 | 0.1031 | 0.012 |
| K02119 | V-type H+-transporting ATPase subunit C [EC:3.6.3.14] | 0.0084 | 0.0117 | 0.014 |
| K02120 | V-type H+-transporting ATPase subunit D [EC:3.6.3.14] | 0.0197 | 0.0272 | 0.009 |
| K02121 | V-type H+-transporting ATPase subunit E [EC:3.6.3.14] | 0.0140 | 0.0179 | 0.048 |
| K02122 | V-type H+-transporting ATPase subunit F [EC:3.6.3.14] | 0.0035 | 0.0052 | 0.010 |
| K02124 | V-type H+-transporting ATPase subunit K [EC:3.6.3.14] | 0.0367 | 0.0443 | 0.017 |
| K02217 | ferritin [EC:1.16.3.1] | 0.0043 | 0.0067 | 0.031 |
| K02227 | adenosylcobinamide-phosphate synthase CobD [EC:6.3.1.10] | 0.0045 | 0.0041 | 0.022 |
| K02303 | uroporphyrin-III C-methyltransferase [EC:2.1.1.107] | 0.0017 | 0.0029 | 0.033 |
| K02322 | DNA polymerase II large subunit [EC:2.7.7.7] | 0.0092 | 0.0149 | 0.027 |
| K02335 | DNA polymerase I [EC:2.7.7.7] | 0.1505 | 0.1373 | 0.035 |
| K02337 | DNA polymerase III subunit alpha [EC:2.7.7.7] | 0.2997 | 0.2796 | 0.021 |
| K02437 | glycine cleavage system H protein | 0.0115 | 0.0108 | 0.050 |
| K02503 | Hit-like protein involved in cell-cycle regulation | 0.0172 | 0.0141 | 0.017 |
| K02520 | translation initiation factor IF-3 | 0.0742 | 0.0697 | 0.018 |
| K02536 | UDP-3-O-[3-hydroxymyristoyl] glucosamine N-acyltransferase [EC:2.3.1.-] | 0.0331 | 0.0288 | 0.031 |
| K02548 | 1,4-dihydroxy-2-naphthoate octaprenyltransferase [EC:2.5.1.74] | 0.0084 | 0.0070 | 0.039 |
| K02600 | N utilization substance protein A | 0.1454 | 0.1370 | 0.048 |
| K02622 | topoisomerase IV subunit B [EC:5.99.1.-] | 0.1251 | 0.1156 | 0.017 |
| K02837 | peptide chain release factor RF-3 | 0.1436 | 0.1369 | 0.050 |
| K02838 | ribosome recycling factor | 0.0300 | 0.0266 | 0.023 |
| K02866 | large subunit ribosomal protein L10e | 0.0100 | 0.0138 | 0.026 |
| K02869 | large subunit ribosomal protein L12 | 0.0090 | 0.0135 | 0.011 |
| K02877 | large subunit ribosomal protein L15e | 0.0173 | 0.0272 | 0.004 |
| K02883 | large subunit ribosomal protein L18e | 0.0032 | 0.0053 | 0.031 |
| K02885 | large subunit ribosomal protein L19e | 0.0099 | 0.0146 | 0.021 |
| K02896 | large subunit ribosomal protein L24e | 0.0035 | 0.0050 | 0.010 |
| K02897 | large subunit ribosomal protein L25 | 0.0368 | 0.0330 | 0.022 |
| K02904 | large subunit ribosomal protein L29 | 0.0235 | 0.0279 | 0.003 |
| K02906 | large subunit ribosomal protein L3 | 0.2618 | 0.2759 | 0.030 |
| K02907 | large subunit ribosomal protein L30 | 0.0277 | 0.0311 | 0.032 |
| K02908 | large subunit ribosomal protein L30e | 0.0060 | 0.0097 | 0.018 |
| K02910 | large subunit ribosomal protein L31e | 0.0034 | 0.0045 | 0.010 |
| K02912 | large subunit ribosomal protein L32e | 0.0076 | 0.0118 | 0.005 |
| K02915 | large subunit ribosomal protein L34e | 0.0065 | 0.0091 | 0.029 |
| K02921 | large subunit ribosomal protein L37Ae | 0.0070 | 0.0100 | 0.006 |
| K02924 | large subunit ribosomal protein L39e | 0.0061 | 0.0086 | 0.019 |
| K02927 | large subunit ribosomal protein L40e | 0.0045 | 0.0064 | 0.000 |
| K02929 | large subunit ribosomal protein L44e | 0.0116 | 0.0162 | 0.007 |
| K02930 | large subunit ribosomal protein L4e | 0.0143 | 0.0244 | 0.006 |
| K02936 | large subunit ribosomal protein L7Ae | 0.0100 | 0.0138 | 0.005 |
| K02956 | small subunit ribosomal protein S15 | 0.0503 | 0.0544 | 0.001 |
| K02962 | small subunit ribosomal protein S17e | 0.0034 | 0.0052 | 0.021 |
| K02966 | small subunit ribosomal protein S19e | 0.0101 | 0.0140 | 0.038 |
| K02974 | small subunit ribosomal protein S24e | 0.0019 | 0.0041 | 0.041 |
| K02979 | small subunit ribosomal protein S28e | 0.0056 | 0.0072 | 0.029 |
| K02984 | small subunit ribosomal protein S3Ae | 0.0139 | 0.0195 | 0.032 |
| K02987 | small subunit ribosomal protein S4e | 0.0093 | 0.0127 | 0.009 |
| K02991 | small subunit ribosomal protein S6e | 0.0051 | 0.0065 | 0.027 |
| K02992 | small subunit ribosomal protein S7 | 0.1734 | 0.1809 | 0.041 |
| K02995 | small subunit ribosomal protein S8e | 0.0075 | 0.0115 | 0.005 |
| K02996 | small subunit ribosomal protein S9 | 0.0892 | 0.0918 | 0.043 |
| K03041 | DNA-directed RNA polymerase subunit Aprime [EC:2.7.7.6] | 0.0210 | 0.0328 | 0.012 |
| K03042 | DNA-directed RNA polymerase subunit Aprime [EC:2.7.7.6] | 0.0171 | 0.0257 | 0.007 |
| K03044 | DNA-directed RNA polymerase subunit Bprime [EC:2.7.7.6] | 0.0321 | 0.0505 | 0.005 |
| K03045 | DNA-directed RNA polymerase subunit Bprime [EC:2.7.7.6] | 0.0208 | 0.0320 | 0.016 |
| K03047 | DNA-directed RNA polymerase subunit D [EC:2.7.7.6] | 0.0082 | 0.0121 | 0.005 |
| K03055 | DNA-directed RNA polymerase subunit K [EC:2.7.7.6] | 0.0010 | 0.0016 | 0.014 |
| K03070 | preprotein translocase subunit SecA | 0.4469 | 0.4226 | 0.018 |
| K03073 | preprotein translocase subunit SecE | 0.0063 | 0.0049 | 0.011 |
| K03075 | preprotein translocase subunit SecG | 0.0228 | 0.0197 | 0.048 |
| K03113 | translation initiation factor eIF-1 | 0.0067 | 0.0102 | 0.004 |
| K03120 | transcription initiation factor TFIID TATA-box-binding protein | 0.0170 | 0.0265 | 0.013 |
| K03124 | transcription initiation factor TFIIB | 0.0143 | 0.0225 | 0.016 |
| K03147 | thiamine biosynthesis protein ThiC | 0.0971 | 0.1092 | 0.040 |
| K03150 | thiamine biosynthesis ThiH | 0.0545 | 0.0496 | 0.024 |
| K03166 | DNA topoisomerase VI subunit A [EC:5.99.1.3] | 0.0132 | 0.0209 | 0.006 |
| K03167 | DNA topoisomerase VI subunit B [EC:5.99.1.3] | 0.0107 | 0.0166 | 0.014 |
| K03217 | preprotein translocase subunit YidC | 0.1122 | 0.1055 | 0.026 |
| K03231 | elongation factor EF-1 alpha subunit [EC:3.6.5.3] | 0.0403 | 0.0591 | 0.006 |
| K03234 | elongation factor EF-2 [EC:3.6.5.3] | 0.0350 | 0.0529 | 0.004 |
| K03236 | translation initiation factor eIF-1A | 0.0079 | 0.0116 | 0.010 |
| K03237 | translation initiation factor eIF-2 alpha subunit | 0.0157 | 0.0229 | 0.018 |
| K03242 | translation initiation factor eIF-2 gamma subunit | 0.0159 | 0.0226 | 0.010 |
| K03243 | translation initiation factor eIF-5B | 0.0064 | 0.0101 | 0.022 |
| K03264 | translation initiation factor eIF-6 | 0.0023 | 0.0039 | 0.014 |
| K03265 | peptide chain release factor eRF subunit 1 | 0.0022 | 0.0040 | 0.024 |
| K03281 | chloride channel protein, CIC family | 0.0499 | 0.0442 | 0.039 |
| K03289 | MFS transporter, NHS family, nucleoside permease | 0.0314 | 0.0248 | 0.041 |
| K03315 | Na+:H+ antiporter, NhaC family | 0.0229 | 0.0197 | 0.038 |
| K03340 | diaminopimelate dehydrogenase [EC:1.4.1.16] | 0.0743 | 0.0695 | 0.035 |
| K03388 | heterodisulfide reductase subunit A [EC:1.8.98.1] | 0.0751 | 0.1073 | 0.006 |
| K03389 | heterodisulfide reductase subunit B [EC:1.8.98.1] | 0.0200 | 0.0275 | 0.019 |
| K03390 | heterodisulfide reductase subunit C [EC:1.8.98.1] | 0.0100 | 0.0153 | 0.015 |
| K03422 | methyl-coenzyme M reductase subunit D | 0.0011 | 0.0020 | 0.031 |
| K03432 | proteasome alpha subunit [EC:3.4.25.1] | 0.0126 | 0.0186 | 0.005 |
| K03433 | proteasome beta subunit [EC:3.4.25.1] | 0.0101 | 0.0153 | 0.017 |
| K03455 | monovalent cation:H+ antiporter-2, CPA2 family | 0.0342 | 0.0298 | 0.042 |
| K03530 | DNA-binding protein HU-beta | 0.0405 | 0.0379 | 0.034 |
| K03545 | trigger factor | 0.0919 | 0.0839 | 0.037 |
| K03596 | GTP-binding protein LepA | 0.1392 | 0.1330 | 0.007 |
| K03615 | electron transport complex protein RnfC | 0.0351 | 0.0315 | 0.025 |
| K03624 | transcription elongation factor GreA | 0.0286 | 0.0259 | 0.028 |
| K03625 | N utilization substance protein B | 0.0242 | 0.0210 | 0.014 |
| K03639 | molybdenum cofactor biosynthesis protein | 0.0019 | 0.0034 | 0.020 |
| K03679 | exosome complex component RRP4 | 0.0151 | 0.0227 | 0.007 |
| K03686 | molecular chaperone DnaJ | 0.0523 | 0.0484 | 0.029 |
| K03698 | CMP-binding protein | 0.0012 | 0.0014 | 0.016 |
| K03724 | ATP-dependent helicase Lhr and Lhr-like helicase [EC:3.6.4.-] | 0.0028 | 0.0045 | 0.025 |
| K03726 | helicase [EC:3.6.4.-] | 0.0013 | 0.0021 | 0.002 |
| K03733 | integrase/recombinase XerC | 0.0193 | 0.0152 | 0.042 |
| K03738 | aldehyde:ferredoxin oxidoreductase [EC:1.2.7.5] | 0.0056 | 0.0077 | 0.010 |
| K03927 | carboxylesterase type B [EC:3.1.1.1] | 0.0734 | 0.0673 | 0.025 |
| K03977 | GTP-binding protein | 0.0874 | 0.0817 | 0.047 |
| K04069 | pyruvate formate lyase activating enzyme [EC:1.97.1.4] | 0.0505 | 0.0417 | 0.021 |
| K04076 | Lon-like ATP-dependent protease [EC:3.4.21.-] | 0.0125 | 0.0187 | 0.023 |
| K04483 | DNA repair protein RadA | 0.0141 | 0.0221 | 0.015 |
| K04566 | lysyl-tRNA synthetase, class I [EC:6.1.1.6] | 0.0088 | 0.0137 | 0.024 |
| K04652 | hydrogenase nickel incorporation protein HypB | 0.0019 | 0.0033 | 0.033 |
| K04655 | hydrogenase expression/formation protein HypE | 0.0050 | 0.0067 | 0.048 |
| K04656 | hydrogenase maturation protein HypF | 0.0029 | 0.0048 | 0.031 |
| K04751 | nitrogen regulatory protein P-II 1 | 0.0302 | 0.0271 | 0.010 |
| K04764 | integration host factor subunit alpha | 0.0083 | 0.0058 | 0.032 |
| K04796 | small nuclear ribonucleoprotein | 0.0056 | 0.0078 | 0.005 |
| K04797 | prefoldin alpha subunit | 0.0026 | 0.0041 | 0.015 |
| K04798 | prefoldin beta subunit | 0.0016 | 0.0028 | 0.019 |
| K04801 | replication factor C small subunit | 0.0034 | 0.0059 | 0.013 |
| K04802 | proliferating cell nuclear antigen | 0.0125 | 0.0188 | 0.009 |
| K05795 | tellurium resistance protein TerD | 0.0011 | 0.0012 | 0.016 |
| K05884 | (R)-2-hydroxyacid dehydrogenase [EC:1.1.1.272] | 0.0035 | 0.0050 | 0.012 |
| K06041 | arabinose-5-phosphate isomerase [EC:5.3.1.13] | 0.0161 | 0.0128 | 0.006 |
| K06142 | outer membrane protein | 0.0612 | 0.0544 | 0.027 |
| K06174 | ATP-binding cassette, sub-family E, member 1 | 0.0090 | 0.0151 | 0.034 |
| K06207 | GTP-binding protein | 0.1921 | 0.1794 | 0.028 |
| K06215 | pyridoxine biosynthesis protein [EC:4.-.-.-] | 0.0555 | 0.0491 | 0.011 |
| K06217 | phosphate starvation-inducible protein PhoH and related proteins | 0.0470 | 0.0460 | 0.040 |
| K06863 | 5-formaminoimidazole-4-carboxamide-1-(beta)-D-ribofuranosyl 5prime-monophosphate synthetase [EC:6.3.4.-] | 0.0110 | 0.0159 | 0.036 |
| K06885 | uncharacterized protein | 0.0327 | 0.0248 | 0.016 |
| K06901 | putative MFS transporter, AGZA family, xanthine/uracil permease | 0.0439 | 0.0424 | 0.035 |
| K06932 | tRNA(Ile2)-agmatinylcytidine synthase | 0.0036 | 0.0068 | 0.033 |
| K06990 | MEMO1 family protein | 0.0359 | 0.0284 | 0.037 |
| K07033 | uncharacterized protein | 0.0020 | 0.0026 | 0.046 |
| K07041 | uncharacterized protein | 0.0117 | 0.0196 | 0.013 |
| K07056 | 16S rRNA (cytidine1402-2'-O)-methyltransferase | 0.0254 | 0.0246 | 0.025 |
| K07068 | uncharacterized protein | 0.0067 | 0.0088 | 0.043 |
| K07079 | uncharacterized protein | 0.0453 | 0.0389 | 0.037 |
| K07085 | putative transport protein | 0.0898 | 0.0796 | 0.023 |
| K07095 | uncharacterized protein | 0.0215 | 0.0190 | 0.030 |
| K07114 | uncharacterized protein | 0.0797 | 0.0683 | 0.015 |
| K07158 | uncharacterized protein | 0.0023 | 0.0037 | 0.002 |
| K07159 | uncharacterized protein | 0.0013 | 0.0022 | 0.009 |
| K07164 | uncharacterized protein | 0.0404 | 0.0342 | 0.021 |
| K07321 | CO dehydrogenase maturation factor | 0.0103 | 0.0134 | 0.018 |
| K07334 | proteic killer suppression protein | 0.0031 | 0.0025 | 0.020 |
| K07388 | hydrogenase expression/formation protein | 0.0050 | 0.0087 | 0.013 |
| K07444 | putative N6-adenine-specific DNA methylase [EC:2.1.1.-] | 0.0790 | 0.0696 | 0.034 |
| K07462 | single-stranded-DNA-specific exonuclease [EC:3.1.-.-] | 0.1066 | 0.1006 | 0.047 |
| K07572 | putative nucleotide binding protein | 0.0016 | 0.0031 | 0.020 |
| K07582 | hypothetical protein | 0.0048 | 0.0069 | 0.033 |
| K07588 | LAO/AO transport system kinase [EC:2.7.-.-] | 0.0256 | 0.0218 | 0.029 |
| K07636 | two-component system, OmpR family, phosphate regulon sensor histidine kinase PhoR [EC:2.7.13.3] | 0.0673 | 0.0589 | 0.035 |
| K07722 | CopG family transcriptional regulator, nickel-responsive regulator | 0.0014 | 0.0023 | 0.029 |
| K07739 | elongator complex protein 3 [EC:2.3.1.48] | 0.0023 | 0.0044 | 0.003 |
| K07792 | anaerobic C4-dicarboxylate transporter DcuB | 0.0279 | 0.0250 | 0.027 |
| K08094 | 6-phospho-3-hexuloisomerase [EC:5.3.1.27] | 0.0014 | 0.0025 | 0.014 |
| K08218 | MFS transporter, PAT family, beta-lactamase induction signal transducer AmpG | 0.0247 | 0.0217 | 0.048 |
| K09013 | Fe-S cluster assembly ATP-binding protein | 0.1145 | 0.1069 | 0.032 |
| K09014 | Fe-S cluster assembly protein SufB | 0.1638 | 0.1561 | 0.020 |
| K09015 | Fe-S cluster assembly protein SufD | 0.0822 | 0.0759 | 0.036 |
| K09142 | hypothetical protein | 0.0155 | 0.0229 | 0.007 |
| K09154 | hypothetical protein | 0.0271 | 0.0428 | 0.002 |
| K09687 | antibiotic transport system ATP-binding protein | 0.0056 | 0.0069 | 0.007 |
| K09733 | hypothetical protein | 0.0070 | 0.0117 | 0.006 |
| K09748 | hypothetical protein | 0.0234 | 0.0204 | 0.044 |
| K09955 | hypothetical protein | 0.1801 | 0.1765 | 0.047 |
| K10726 | replicative DNA helicase Mcm [EC:3.6.4.-] | 0.0115 | 0.0170 | 0.010 |
| K11261 | formylmethanofuran dehydrogenase subunit E [EC:1.2.99.5] | 0.0010 | 0.0017 | 0.012 |
| K11600 | exosome complex component RRP41 | 0.0091 | 0.0144 | 0.005 |
| K11646 | dehydroquinate synthase II [EC:1.4.1.-] | 0.0025 | 0.0043 | 0.005 |
| K12589 | exosome complex component RRP42 | 0.0068 | 0.0105 | 0.015 |
| K13038 | phosphopantothenoylcysteine decarboxylase / phosphopantothenate--cysteine ligase [EC:4.1.1.36 6.3.2.5] | 0.0450 | 0.0410 | 0.021 |
| K13525 | transitional endoplasmic reticulum ATPase | 0.0141 | 0.0233 | 0.026 |
| K13942 | 5,10-methenyltetrahydromethanopterin hydrogenase [EC:1.12.98.2] | 0.0205 | 0.0370 | 0.008 |
| K13953 | alcohol dehydrogenase, propanol-preferring [EC:1.1.1.1] | 0.0033 | 0.0074 | 0.031 |
| K14126 | F420-non-reducing hydrogenase subunit A [EC:1.12.99.-] | 0.0336 | 0.0496 | 0.011 |
| K14127 | F420-non-reducing hydrogenase iron-sulfur subunit D [EC:1.12.99.-] | 0.0220 | 0.0312 | 0.030 |
| K14128 | F420-non-reducing hydrogenase subunit G [EC:1.12.99.-] | 0.0234 | 0.0331 | 0.007 |
| K14228 | tRNA Leu | 0.0679 | 0.0698 | 0.027 |
| K14234 | tRNA Thr | 0.0336 | 0.0348 | 0.013 |
| **Comparison between Low- compared to High-SFA groups for the 8*8 extreme animals** | | | | |
| **KEGG ID** | **FUNCTION** | **Mean LOW** | **Mean HIGH** | **P-value** |
| K00100 | butanol dehydrogenase | 0.001152 | 0.001849 | 0.047527 |
| K00383 | glutathione reductase (NADPH) [EC:1.8.1.7] | 0.000711 | 0.002018 | 0.028076 |
| K00525 | ribonucleoside-diphosphate reductase alpha chain [EC:1.17.4.1] | 0.002046 | 0.003417 | 0.037709 |
| K00688 | starch phosphorylase [EC:2.4.1.1] | 0.308631 | 0.288747 | 0.039055 |
| K00864 | glycerol kinase [EC:2.7.1.30] | 0.001346 | 0.002707 | 0.012539 |
| K01079 | phosphoserine phosphatase [EC:3.1.3.3] | 0.070768 | 0.089106 | 0.029791 |
| K01126 | glycerophosphoryl diester phosphodiesterase [EC:3.1.4.46] | 0.000716 | 0.001726 | 0.012895 |
| K01516 | nucleoside-triphosphatase [EC:3.6.1.15] | 0.028903 | 0.037425 | 0.032284 |
| K01679 | fumarate hydratase, class II [EC:4.2.1.2] | 0.002082 | 0.003166 | 0.020607 |
| K01682 | aconitate hydratase 2 [EC:4.2.1.3] | 0.001609 | 0.000821 | 0.042397 |
| K01709 | CDP-glucose 4,6-dehydratase [EC:4.2.1.45] | 0.005642 | 0.00758 | 0.031406 |
| K01778 | diaminopimelate epimerase [EC:5.1.1.7] | 0.015791 | 0.018611 | 0.03578 |
| K01873 | valyl-tRNA synthetase [EC:6.1.1.9] | 0.30154 | 0.284606 | 0.016948 |
| K01875 | seryl-tRNA synthetase [EC:6.1.1.11] | 0.19184 | 0.170984 | 0.000515 |
| K01879 | glycyl-tRNA synthetase beta chain [EC:6.1.1.14] | 0.000645 | 0.001577 | 0.046157 |
| K01881 | prolyl-tRNA synthetase [EC:6.1.1.15] | 0.24027 | 0.224581 | 0.03195 |
| K01939 | adenylosuccinate synthase [EC:6.3.4.4] | 0.135778 | 0.123829 | 0.027288 |
| K01992 | ABC-2 type transport system permease protein | 0.001207 | 0.002511 | 0.019255 |
| K02056 | simple sugar transport system ATP-binding protein [EC:3.6.3.17] | 0.000924 | 0.001754 | 0.012569 |
| K02230 | cobaltochelatase CobN [EC:6.6.1.2] | 0.00052 | 0.001788 | 0.020699 |
| K02334 | DNA polymerase bacteriophage-type [EC:2.7.7.7] | 0.003631 | 0.00623 | 0.013235 |
| K02520 | translation initiation factor IF-3 | 0.077556 | 0.070299 | 0.036312 |
| K02912 | large subunit ribosomal protein L32e | 0.007229 | 0.01278 | 0.04156 |
| K02956 | small subunit ribosomal protein S15 | 0.049093 | 0.054771 | 0.048746 |
| K03047 | DNA-directed RNA polymerase subunit D [EC:2.7.7.6] | 0.007421 | 0.012824 | 0.042467 |
| K03070 | preprotein translocase subunit SecA | 0.453087 | 0.416449 | 0.000405 |
| K03596 | GTP-binding protein LepA | 0.14148 | 0.129415 | 0.003236 |
| K03738 | aldehyde:ferredoxin oxidoreductase [EC:1.2.7.5] | 0.005229 | 0.008021 | 0.044605 |
| K03778 | D-lactate dehydrogenase [EC:1.1.1.28] | 0.000621 | 0.001743 | 0.03709 |
| K03885 | NADH dehydrogenase [EC:1.6.99.3] | 0.002047 | 0.004066 | 0.023407 |
| K04042 | bifunctional UDP-N-acetylglucosamine pyrophosphorylase / Glucosamine-1-phosphate N-acetyltransferase [EC:2.7.7.23 2.3.1.157] | 0.004141 | 0.00626 | 0.049253 |
| K04653 | hydrogenase expression/formation protein HypC | 0.001525 | 0.002948 | 0.041332 |
| K05795 | tellurium resistance protein TerD | 0.000919 | 0.001369 | 0.033239 |
| K06148 | ATP-binding cassette, subfamily C, bacterial | 0.00098 | 0.003384 | 0.021817 |
| K07158 | uncharacterized protein | 0.001968 | 0.003886 | 0.008114 |
| K07482 | transposase, IS30 family | 0.000635 | 0.002638 | 0.02368 |
| K09154 | hypothetical protein | 0.023807 | 0.044373 | 0.048477 |
| K09687 | antibiotic transport system ATP-binding protein | 0.005469 | 0.00737 | 0.044817 |
| K13942 | 5,10-methenyltetrahydromethanopterin hydrogenase [EC:1.12.98.2] | 0.017319 | 0.039115 | 0.037622 |
| K13953 | alcohol dehydrogenase, propanol-preferring [EC:1.1.1.1] | 0.002989 | 0.007885 | 0.026209 |

**Table S8.** Partial Least Square results between selected milk fatty acids and microbial genes.

| **Type of fatty acid: SFA** | | **Percentage of variability explained: 79.6%** | |
| --- | --- | --- | --- |
| **VIP*** | **Coefficient** | KEGG ID | FUNCTION |
| 1.25 | -0.26 | K03596 | GTP-binding protein LepA |
| 1.21 | -0.25 | K03070 | preprotein translocase subunit SecA |
| 1.17 | -0.19 | K01875 | seryl-tRNA synthetase [EC:6.1.1.11] |
| 0.99 | 0.16 | K01679 | fumarate hydratase, class II [EC:4.2.1.2] |
| 0.97 | 0.04 | K00383 | glutathione reductase (NADPH) [EC:1.8.1.7] |
| 0.95 | 0.03 | K06148 | ATP-binding cassette, subfamily C, bacterial |
| 0.89 | 0.06 | K07482 | transposase, IS30 family |
| 0.89 | 0.09 | K01126 | glycerophosphoryl diester phosphodiesterase [EC:3.1.4.46] |
| 0.78 | 0.05 | K03778 | D-lactate dehydrogenase [EC:1.1.1.28] |
| **Type of fatty acid: C12:0** | | **Percentage of variability explained: 84.4%** | |
| **VIP*** | **Coefficient** | KEGG ID | FUNCTION |
| 1.22 | -0.22 | K01875 | seryl-tRNA synthetase [EC:6.1.1.11] |
| 1.20 | -0.29 | K03070 | preprotein translocase subunit SecA |
| 1.03 | 0.27 | K14223 | tRNA Gln |
| 1.00 | 0.04 | K06148 | ATP-binding cassette, subfamily C, bacterial |
| 0.96 | 0.03 | K00215 | dihydrodipicolinate reductase [EC:1.3.1.26] |
| 0.96 | 0.19 | K01126 | glycerophosphoryl diester phosphodiesterase [EC:3.1.4.46] |
| 0.91 | 0.01 | K00383 | glutathione reductase (NADPH) [EC:1.8.1.7] |
| 0.91 | 0.06 | K07482 | transposase, IS30 family |
| 0.89 | 0.03 | K03531 | cell division protein FtsZ |
| 0.84 | -0.13 | K01923 | phosphoribosylaminoimidazole-succinocarboxamide synthase [EC:6.3.2.6] |
| **Type of fatty acid: C14:0** | | **Percentage of variability explained: 76.3%** | |
| **VIP*** | **Coefficient** | KEGG ID | FUNCTION |
| 1.21 | -0.23 | K03070 | preprotein translocase subunit SecA |
| 1.17 | -0.18 | K01875 | seryl-tRNA synthetase [EC:6.1.1.11] |
| 1.12 | -0.28 | K00133 | aspartate-semialdehyde dehydrogenase [EC:1.2.1.11] |
| 1.03 | 0.16 | K01126 | glycerophosphoryl diester phosphodiesterase [EC:3.1.4.46] |
| 0.98 | 0.20 | K14223 | tRNA Gln |
| 0.94 | 0.06 | K06148 | ATP-binding cassette, subfamily C, bacterial |
| 0.89 | 0.03 | K00383 | glutathione reductase (NADPH) [EC:1.8.1.7] |
| 0.89 | 0.06 | K07482 | transposase, IS30 family |
| 0.85 | -0.12 | K01923 | phosphoribosylaminoimidazole-succinocarboxamide synthase [EC:6.3.2.6] |
| 0.83 | 0.08 | K00525 | ribonucleoside-diphosphate reductase alpha chain [EC:1.17.4.1] |
| **Type of fatty acid: C16:0** | | **Percentage of variability explained: 94.1%** | |
| **VIP*** | **Coefficient** | **KEGG ID** | **FUNCTION** |
| 1.19 | 0.25 | K02956 | small subunit ribosomal protein S15 |
| 1.13 | 0.18 | K07496 | putative transposase |
| 1.09 | 0.24 | K04761 | LysR family transcriptional regulator, hydrogen peroxide-inducible genes activator |
| 1.08 | 0.18 | K02334 | DNA polymerase bacteriophage-type [EC:2.7.7.7] |
| 1.03 | 0.13 | K01679 | fumarate hydratase, class II [EC:4.2.1.2] |
| 1.03 | 0.20 | K05795 | tellurium resistance protein TerD |
| 0.91 | 0.06 | K07030 | uncharacterized protein |
| 0.88 | 0.06 | K00383 | glutathione reductase (NADPH) [EC:1.8.1.7] |
| **Type of fatty acid: ALNA** | | **Percentage of variability explained: 86.7%** | |
| **VIP*** | **Coefficient** | **KEGG ID** | **FUNCTION** |
| 1.39 | -0.29 | K02334 | DNA polymerase bacteriophage-type [EC:2.7.7.7] |
| 1.20 | -0.23 | K09687 | antibiotic transport system ATP-binding protein |
| 1.19 | 0.23 | K03596 | GTP-binding protein LepA |
| 1.09 | -0.13 | K01679 | fumarate hydratase, class II [EC:4.2.1.2] |
| 1.02 | -0.24 | K03569 | rod shape-determining protein MreB and related proteins |
| 0.87 | 0.00 | K00383 | glutathione reductase (NADPH) [EC:1.8.1.7] |
| 0.77 | -0.04 | K01961 | acetyl-CoA carboxylase, biotin carboxylase subunit [EC:6.4.1.2 6.3.4.14] |
| 0.77 | -0.10 | K03325 | arsenite transporter, ACR3 family |
| 0.76 | -0.01 | K07482 | transposase, IS30 family |
| 0.71 | -0.05 | K01126 | glycerophosphoryl diester phosphodiesterase [EC:3.1.4.46] |
| **Type of fatty acid: MUFA** | | **Percentage of variability explained: 85.7%** | |
| **VIP*** | **Coefficient** | **KEGG ID** | **FUNCTION** |
| 1.21 | 0.24 | K03070 | preprotein translocase subunit SecA |
| 1.20 | 0.25 | K03596 | GTP-binding protein LepA |
| 1.16 | 0.18 | K01875 | seryl-tRNA synthetase [EC:6.1.1.11] |
| 1.08 | -0.25 | K03569 | rod shape-determining protein MreB and related proteins |
| 0.93 | -0.05 | K00383 | glutathione reductase (NADPH) [EC:1.8.1.7] |
| 0.93 | -0.05 | K06148 | ATP-binding cassette, subfamily C, bacterial |
| 0.91 | -0.11 | K01126 | glycerophosphoryl diester phosphodiesterase [EC:3.1.4.46] |
| 0.90 | -0.04 | K07482 | transposase, IS30 family |
| 0.80 | -0.05 | K03778 | D-lactate dehydrogenase [EC:1.1.1.28] |
| **Type of fatty acid: PUFA** | | **Percentage of variability explained: 89.1%** | |
| **VIP*** | **Coefficient** | **KEGG ID** | **FUNCTION** |
| 1.26 | 0.23 | K06020 | energy-dependent translational throttle protein EttA |
| 1.17 | 0.17 | K01682 | aconitate hydratase 2 [EC:4.2.1.3] |
| 1.15 | -0.18 | K01625 | 2-dehydro-3-deoxyphosphogluconate aldolase / 4-hydroxy-2-oxoglutarate aldolase [EC:4.1.2.14 4.1.3.16] |
| 1.07 | -0.34 | K03698 | CMP-binding protein |
| 1.04 | 0.07 | K07080 | #N/A |
| 0.92 | 0.13 | K01523 | phosphoribosyl-ATP pyrophosphohydrolase [EC:3.6.1.31] |
| 0.87 | -0.15 | K09922 | hypothetical protein |
| 0.80 | 0.14 | K01876 | aspartyl-tRNA synthetase [EC:6.1.1.12] |
| **Type of fatty acid: n-3** | | **Percentage of variability explained: 81.9%** | |
| **VIP*** | **Coefficient** | **KEGG ID** | **FUNCTION** |
| 1.20 | -0.37 | K03569 | rod shape-determining protein MreB and related proteins |
| 1.14 | -0.19 | K02334 | DNA polymerase bacteriophage-type [EC:2.7.7.7] |
| 1.09 | 0.21 | K00919 | 4-diphosphocytidyl-2-C-methyl-D-erythritol kinase [EC:2.7.1.148] |
| 1.05 | 0.00 | K01679 | fumarate hydratase, class II [EC:4.2.1.2] |
| 1.05 | 0.05 | K03596 | GTP-binding protein LepA |
| 1.04 | -0.09 | K01875 | seryl-tRNA synthetase [EC:6.1.1.11] |
| 1.01 | -0.20 | K00100 | butanol dehydrogenase |
| 0.82 | -0.21 | K03325 | arsenite transporter, ACR3 family |
| 0.81 | -0.12 | K00088 | IMP dehydrogenase [EC:1.1.1.205] |

* Variable importance for projection (VIP)
